# Comparative evaluation of methods for the prediction of protein–ligand binding sites

**DOI:** 10.1186/s13321-024-00923-z

**Published:** 2024-11-11

**Authors:** Javier S. Utgés, Geoffrey J. Barton

**Affiliations:** Division of Computational Biology, School of Life Sciences, University of Dundee, Dow Street, Dundee, DD1 5EH Scotland, UK

**Keywords:** Ligand binding site prediction, Binding pocket, Benchmark, Reference dataset, Machine learning, Drug discovery

## Abstract

The accurate identification of protein–ligand binding sites is of critical importance in understanding and modulating protein function. Accordingly, ligand binding site prediction has remained a research focus for over three decades with over 50 methods developed and a change of paradigm from geometry-based to machine learning. In this work, we collate 13 ligand binding site predictors, spanning 30 years, focusing on the latest machine learning-based methods such as VN-EGNN, IF-SitePred, GrASP, PUResNet, and DeepPocket and compare them to the established P2Rank, PRANK and fpocket and earlier methods like PocketFinder, Ligsite and Surfnet. We benchmark the methods against the human subset of our new curated reference dataset, LIGYSIS. LIGYSIS is a comprehensive protein–ligand complex dataset comprising 30,000 proteins with bound ligands which aggregates biologically relevant unique protein–ligand interfaces across biological units of multiple structures from the same protein. LIGYSIS is an improvement for testing methods over earlier datasets like sc-PDB, PDBbind, binding MOAD, COACH420 and HOLO4K which either include 1:1 protein–ligand complexes or consider asymmetric units. Re-scoring of fpocket predictions by PRANK and DeepPocket display the highest recall (60%) whilst IF-SitePred presents the lowest recall (39%). We demonstrate the detrimental effect that redundant prediction of binding sites has on performance as well as the beneficial impact of stronger pocket scoring schemes, with improvements up to 14% in recall (IF-SitePred) and 30% in precision (Surfnet). Finally, we propose top-*N*+2 recall as the universal benchmark metric for ligand binding site prediction and urge authors to share not only the source code of their methods, but also of their benchmark.

**Scientific contributions**

This study conducts the largest benchmark of ligand binding site prediction methods to date, comparing 13 original methods and 15 variants using 10 informative metrics. The LIGYSIS dataset is introduced, which aggregates biologically relevant protein–ligand interfaces across multiple structures of the same protein. The study highlights the detrimental effect of redundant binding site prediction and demonstrates significant improvement in recall and precision through stronger scoring schemes. Finally, top-*N*+2 recall is proposed as a universal benchmark metric for ligand binding site prediction, with a recommendation for open-source sharing of both methods and benchmarks.

**Supplementary Information:**

The online version contains supplementary material available at 10.1186/s13321-024-00923-z.

## Introduction

Identifying where ligands can bind to proteins is of critical importance in understanding and modulating protein function. While X-ray crystallography remains the gold-standard to identify and characterise binding sites [1–5], over the last three decades, significant effort has been made to develop computational methods that predict binding sites from an apo three-dimensional protein structure [6].

Prediction methods exploit a variety of different techniques to suggest binding sites. Geometry-based techniques like fpocket [7], Ligsite [8] and Surfnet [9] identify cavities by analysing the geometry of the molecular surface of a protein and usually rely on the use of a grid, gaps, spheres, or tessellation [7–14]. Energy-based methods such as PocketFinder [15] rely on the calculation of interaction energies between the protein and chemical group or probe to identify cavities [15–20]. Conservation-based methods make use of sequence evolutionary conservation information to find patterns in multiple sequence alignments and identify conserved key residues for ligand site identification [21–23]. Template-based methods rely on structural information from homologues and the assumption that structurally conserved proteins might bind ligands at a similar location [24–29]. Combined approaches or meta-predictors combine multiple methods, or the use of multiple types of data to infer ligand binding sites, e.g., geometric features with sequence conservation [30–36]. Finally, machine learning methods utilise a wide range of machine learning techniques including random forest, as well as deep, graph, residual, or convolutional neural networks [37–57]. Machine learning methods comprise the bulk of the methods reviewed in this analysis and are exemplified by PRANK [37], P2Rank [38, 40], DeepPocket [48], PUResNet [44, 57], GrASP [54], IF-SitePred [55] and VN-EGNN [56]. Open source, peer-reviewed and easy to install methods were prioritised (Supplementary Table 1). This set of methods represents the most complete and relevant set of ligand binding site prediction tools benchmarked to date and is representative of the state-of-the-art within the field.

Table 1 summarises the methods evaluated in this work, which were executed with their standard settings. VN-EGNN [56] combines virtual nodes with equivariant graph neural networks. Virtual nodes, represented by ESM-2 embeddings [58] are passed through a series of message-passing layers until they reach their final coordinates, which represent the centroid of predicted pockets. Pocket residues are not reported. IF-SitePred [55] represents protein residues with ESM-IF1 embeddings [59] and employs 40 different LightGBM models [60] to classify residues as ligand-binding if all forty models return a *p* > 0.5. It later utilises PyMOL [61] to place a series of cloud points which are clustered using DBSCAN [62] and a threshold of 1.7 Å. Pocket centroids are obtained by averaging the clustered points’ coordinates, scored and ranked based on the number of cloud points. Like VN-EGNN, no pocket residues are defined. GrASP [54] employs graph attention networks to perform semantic segmentation on all surface protein atoms, represented by 17 atom, residue and bond-level features, scoring which are likely part of a binding site. Atoms with a score > 0.3 are clustered into binding sites using average linkage and a threshold of 15 Å. Pocket scores are calculated as the sum of squares of binding site atom scores. PUResNet [44] combines deep residual and convolutional neural networks to predict ligand binding sites using an 18-element vector of atom-level features and one-hot encoding to represent grid voxels. Voxels with a score > 0.34 are clustered into binding sites using DBSCAN and a threshold of 5.5 Å [57]. Pockets are represented by their residues, but neither pocket centroid, nor score or ranking are reported. Similarly to PUResNet, DeepPocket [48] exploits convolutional neural networks on grid voxels represented by 14 atom-level features to re-score (DeepPocket_RESC_) and additionally extract new pocket shapes (DeepPocket_SEG_) from fpocket candidates. P2Rank [40] relies on solvent accessible surface (SAS) points placed over the protein surface, represented by 35 atom and residue-level features, and a random forest classifier to score them based on their likelihood of binding a ligand. SAS points with a score > 0.35 are clustered into sites using single linkage and a threshold of 3 Å. P2Rank_CONS_ [63] works in the same manner but considers an extra feature: amino acid conservation as measured by Jensen–Shannon divergence [64]. Both report residue and pocket level scores, as well as pocket centroids and rank. PRANK [37] uses the P2Rank algorithm to score/re-score pockets predicted by other methods. In this work, fpocket predictions are re-scored with PRANK (fpocket_PRANK_). PocketFinder [15] uses the Lennard–Jones [65] transformation on a 1 Å grid surrounding the protein surface to predict protein cavities. PocketFinder does not report pocket centroid, score or rank. Finally, geometry-based methods: fpocket [7], Ligsite [8] and Surfnet [9] rely on the geometry of the molecular surface to find cavities. fpocket is the only one of these three methods that reports pocket centroid, score, rank and residues.

**Table 1:** Summary of ligand binding site prediction methods analysed in this study

Method	Approach	Features	# Features	P centroid	P residues	P score	P ranking	R score	R threshold	Cluster	Algorithm	Threshold (Å)
VN-EGNN	EGNN + VN	ESM-2 embeddings	1280	**✓**	**✕**	**✓**	**✓**	**✕**	–	–	–	–
IF-SitePred	LightGBM	ESM-IF1 embeddings	512	**✓**	**✕**	**✓**	**✓**	**✕**	0.5 (ALL 40)	Cloud points	DBSCAN	1.7
GrASP	GAT-GNN	Atom, residue, bond…	17	**✓**	**✕**	**✓**	**✓**	**✓**	0.3	Atoms	Average	15
PUResNet	DRN + 3D-CNN	Atom + one-hot encoding	18	**✕**	**✓**	**✕**	**✕**	**✕**	0.34	Atoms	DBSCAN	5.5
DeepPocket	fpocket + 3D-CNN	Atom	14	**✓**	**✓**	**✓**	**✓**	**✕**	–	–	–	–
P2Rank_CONS_	Random Forest	Atom and residue	36	**✓**	**✓**	**✓**	**✓**	**✓**	0.35	SAS points	Single	3
P2Rank	Random Forest	Atom and residue	35	**✓**	**✓**	**✓**	**✓**	**✓**	0.35	SAS points	Single	3
fpocket_PRANK_	fpocket + Random Forest	Atom and residue	34	**✓**	**✓**	**✓**	**✓**	**✓**	–	–	–	–
fpocket	α-spheres	–	–	**✕**	**✓**	**✓**	**✓**	**✕**	–	α-spheres	Multiple	1.7
PocketFinder^+^	LJ potential	–	–	**✕**	**✕**	**✕**	**✕**	**✓**	–	–	–	–
Ligsite^+^	Cubic grid	–	–	**✕**	**✕**	**✕**	**✕**	**✓**	–	–	–	–
Surfnet^+^	Gap regions	–	–	**✕**	**✕**	**✕**	**✕**	**✓**	–	–	–	–

All these methods were used with their default settings. Check marks (**✓**) indicate that a method provides a given output and crosses (**✕**) the contrary. Dashes (–) indicate a field is not applicable for a given method, e.g., features for non-machine learning-based methods. Approach: the techniques applied by the method; Features/#Features: the features and their number if the method is machine learning-based; P centroid/P residues/P score/P ranking/R score: whether the method reports the pocket centroid, pocket residues, pocket score, pocket ranking and residue *ligandability* score. Information about their clustering strategies is also relevant: whether the method uses a residue ligandability threshold (R threshold), the instances they cluster (Cluster) to define the distinct pockets, the clustering algorithm used (Algorithm) and threshold employed (Threshold). For example, P2Rank uses a random forest classifier on SAS points represented by 35 atom and residue features. Points with a score > 0.35 are later clustered into binding sites using single linkage and a threshold of 3 Å. DeepPocket and fpocket_PRANK_ use fpocket predictions as a starting point and later employ different technologies to re-score or re-define pockets. EGNN + VN: equivariant graph neural network + virtual nodes; LightGBM: light gradient boosting machine; GAT: graph attention network; GNN: graph neural network; DRN: deep residual network; 3D-CNN: three-dimensional convolutional neural network; LJ potential: Lennard–Jones potential

In this work, we have gathered thirteen ligand binding site prediction tools, spanning three decades of research and compared them against our reference dataset LIGYSIS. LIGYSIS extends the work by Utgés et al*.* [5] that identifies human protein–ligand binding sites for biologically relevant ligands, defined by BioLiP [66], from protein structures determined by X-ray crystallography. We compared the thirteen methods to each other and to the LIGYSIS reference dataset according to a range of metrics including the number of ligand sites, their size, shape, proximity, overlap and redundancy. We have explored in detail and demonstrated the importance of a robust pocket scoring scheme and highlight how some of the methods can improve their performance significantly by re-scoring their predictions. This analysis identifies the strengths and weaknesses of prediction assessment metrics and leads to guidance for developing ligand binding site prediction tools or using these methods to understand protein function and in drug development. This work represents the first independent ligand site prediction benchmark for over a decade, since Chen et al. [67] and the largest to date in terms of dataset size (2775), methods compared (13 original + 15 variants) and metrics employed (> 10).

## Results

### The LIGYSIS dataset

The ligand binding site analysis, or LIGYSIS, dataset comprises protein–ligand complexes for 3448 human proteins. For each protein, biologically relevant protein–ligand interactions, in accordance with BioLiP [66], are considered across the PISA-defined [68] biological assemblies of the multiple entries deposited in the PDBe [69]. Ligands are clustered using their protein interaction fingerprint to identify ligand binding sites as described by Utgés et al*.* [5, 70] (see “Methods”). The full LIGYSIS dataset includes ≈ 30,000 proteins with known ligand-bound complexes. Here, we focus on the *human subset* of LIGYSIS as a manageable set to run all prediction methods on and refer to this as *LIGYSIS* for brevity.

**Fig. 1:**
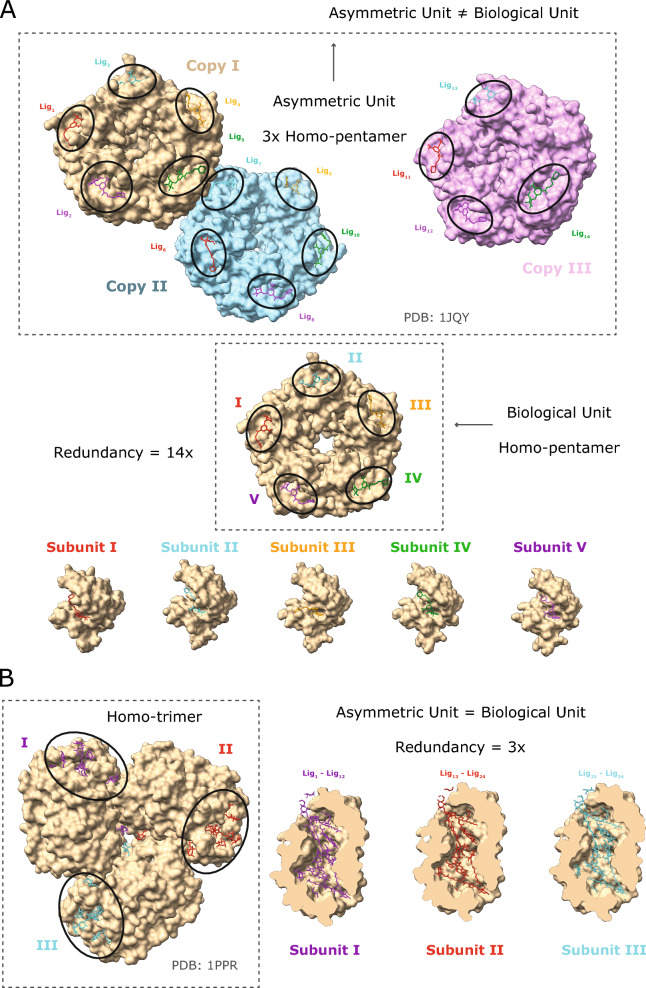
Redundancy in protein–ligand interfaces. Two examples of how the type of macromolecular assembly and difference between the asymmetric and biological unit of a protein–ligand complex results in redundant protein–ligand interfaces. **A** For PDB: 1QJY [72], the asymmetric unit comprises three copies of a homo-pentamer, whereas the biologically functional assembly is a single pentamer. A BMSC-0010 ligand molecule binds to each copy, except for one, of each of the three pentamers. This results in the same protein–ligand interface repeated 14 times, i.e., 14× redundancy; **B** for PDB: 1PPR [73] both the asymmetric and biological units are a homo-trimer. Different molecules of the same ligands are binding to the same interfaces across the three copies of the trimer, i.e., 3× redundancy. Dashed rectangles indicate the asymmetric/biological units

The LIGYSIS dataset differs from previous train and test sets for ligand binding sites by considering biological units, aggregating multiple structures of the same protein, and removing redundant protein–ligand interfaces. The asymmetric unit is the smallest portion of a crystal structure that can reproduce the complete unit cell through a series of symmetry operations. The asymmetric unit often does not correspond to the biological assembly or unit and relying on it can lead to artificial crystal contacts or redundant protein–ligand interfaces. The biological unit is the biologically relevant and functional macromolecular assembly for a given structure and might be formed by one, multiple copies or a portion of the asymmetric unit [71]. LIGYSIS consistently considers biological units, which is key in any analysis that delves into molecular interactions at residue or atomistic level. An example of this illustrated in Fig. 1A is PDB: 1JQY [72], present in the HOLO4K dataset, where the asymmetric unit is formed by three copies of a homo-pentamer, whereas the biological unit comprises a single pentamer. In the asymmetric unit of PDB: 1JQY 14 molecules of BMSC-0010 (A32) interact with 14 copies of *Escherichia coli* heat-labile enterotoxin B chain (P32890). This protein–ligand interface is the same repeated 14 times (Fig. 1A). Protein–ligand interface redundancy can also be an issue when the asymmetric unit equals the biological assembly (Fig. 1B). In PDB: 1PPR [73], also in HOLO4K, molecules of chlorophyll A (CLA), peridinin (PID) and digalactosyl diacyl glycerol (DGD) bind to the three copies of a peridinin-chlorophyll a-binding protein 1, chloroplastic, PCP, (P80484) trimer, resulting in a redundancy of 3×. To account for this, LIGYSIS considers unique non-redundant protein–ligand interfaces by retrieving the UniProt residue numbers of the residues the ligands interact with, so 1/14 interfaces are considered for PDB: 1JQY and 12/36 for PDB: 1PPR. Finally, unique ligand interactions are aggregated across different structures for the same protein and ligand sites defined.

**Fig. 2:**
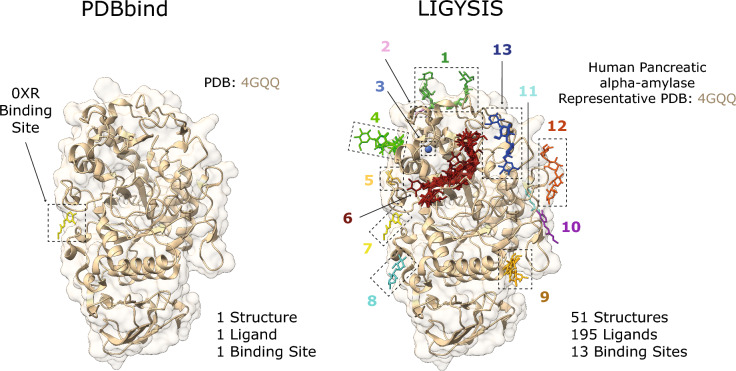
Comparison of PDBbind and LIGYSIS. PDBbind is comprised by complexes between a protein and the most biologically relevant ligand in a structure. For PDB: 4GQQ [74], this is ethyl caffeate (0XR). LIGYSIS considers all unique biologically relevant protein–ligand interactions across all the structures for a given protein. For human pancreatic alpha-amylase (P04746), which representative structure is PDB: 4GQQ, 13 ligand binding sites are defined from 195 ligands across 51 structures. LIGYSIS provides a better representation of the ligand-binding capabilities of a protein than a single protein–ligand complex and constitutes therefore a better benchmark for ligand binding site prediction tools

Figure 2 shows the comparison between PDB: 4GQQ [74], present in the PDBbind dataset, and the LIGYSIS entry for human pancreatic alpha-amylase (P04746), which representative structure is also PDB: 4GQQ. The entry in PDBbind represents a single protein–ligand complex, whereas LIGYSIS makes use of 51 structures, 195 ligands to define 13 different ligand binding sites. LIGYSIS aggregates all unique biologically relevant protein–ligand interactions for a protein in a non-redundant manner, thus representing the most complete and integrative protein–ligand binding dataset up to date. For this reason, we propose LIGYSIS as a new benchmark dataset for the prediction of ligand binding sites and use it in this work to evaluate a set of thirteen ligand binding site prediction and cavity identification tools.

### Comparison of datasets

**Fig. 3:**
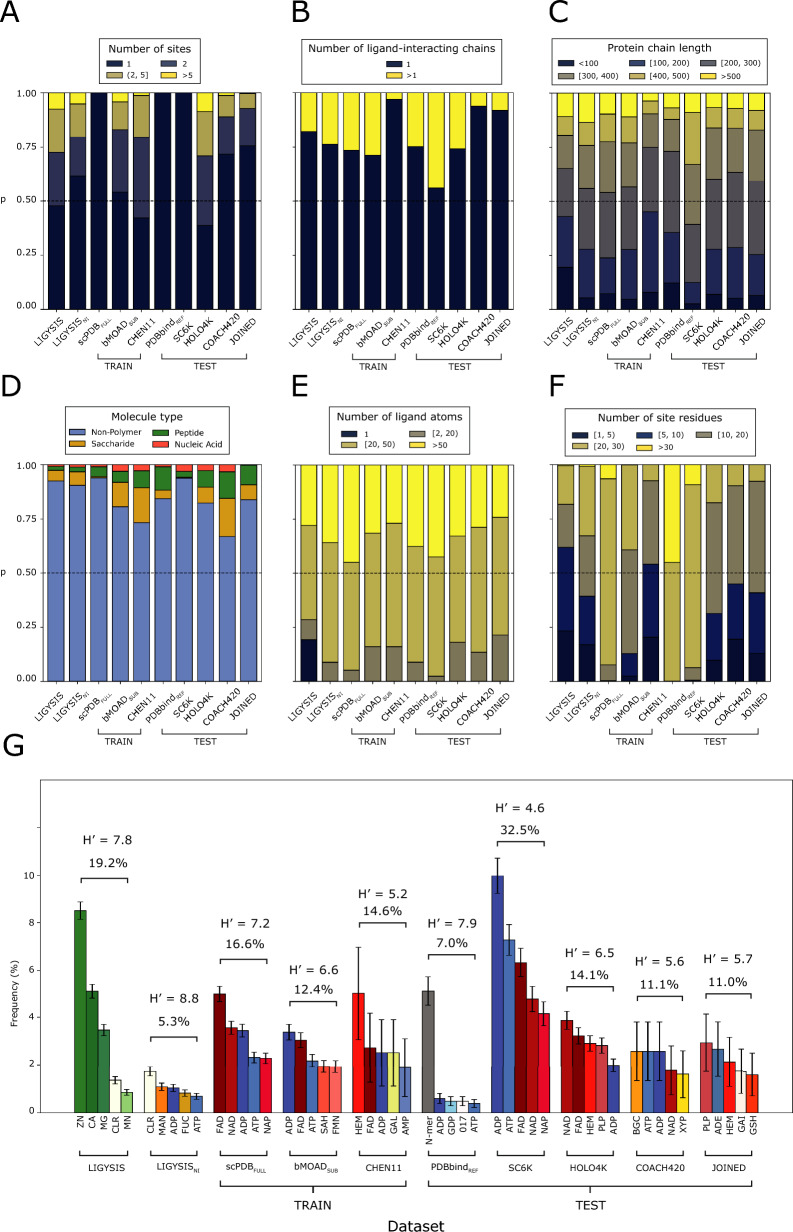
Comparison of datasets. **A**, **B**, **C**, **E** and **F** plot the frequencies of binned intervals of a discrete variable, coloured using the *cividis* palette. Interval ranges were selected to facilitate data interpretation. **A** Number of ligand binding sites per dataset entry; **B** number of ligand-interacting protein chains. This represents whether the ligand interacts with a single protein chain or more; **C** length of ligand-interacting protein chains (number of amino acids); **D** ligand molecule type frequency as described in the CCD; **E** number of ligand atoms; **F** binding site size, i.e., number of ligand-interacting protein residues. Dashed lines drawn at *p* = 0.5; **G** five most frequent ligands per dataset. Error bars represent 95% confidence interval of the proportion [75]. Ligands of similar type are coloured in shades of the same colour: greens for ions, reds for co-factors, blues for energy-carrier molecules, yellows for sugars, grey for peptides and white for other non-polymeric ligands. Above the bars, Shannon’s Entropy and the proportion of all ligands in each set covered by these top-5 can be found. Both are measures of ligand diversity within each dataset. A subset of LIGYSIS with no ions (NI), LIGYSIS_NI_, is included in this analysis, as most training and test datasets do not consider ions. *ADE* adenine, *CLR* cholesterol, *GAI* guanidine, *GSH* glutathione, *MAN* mannose, *FUC* fucose, *NAP* nicotinamide–adenine–dinucleotide phosphate, *SAH* *S*-adenosyl-l-homocysteine, *FMN* flavin mononucleotide, *GAL* galactose, *N-mer* protein peptides of *N* amino acids, *017* darunavir, *PLP* vitamin B6 phosphate, *BGC* glucose, *XYP* xylose

To assess the scope and limitations of the predictive methods surveyed in this work, their training and test sets were compared with LIGYSIS by number of sites per protein, number of interacting protein chains per ligand site, ligand size, ligand site size, and ligand composition. sc-PDB_FULL_ represents the full sc-PDB dataset used for training by DeepPocket, bMOAD_SUB_ the subset of binding MOAD used for training by IF-SitePred and PDBbind_REF_ the reference subset of PDBbind which VN-EGNN uses for testing. Table 2 summarises the size of the datasets, which methods employ them and their overlap with the LIGYSIS set. LIGYSIS differs from all other datasets since biologically relevant ions are considered, comprising ≈ 40% of the ligand sites. For additional reference, LIGYSIS_NI_, a subset of LIGYSIS without ions, is also included in this analysis. Figure 3A shows the number of binding sites per entry across datasets. sc-PDB_FULL_, PDBbind_REF_ and SC6K only consider the most relevant ligand for each entry. COACH420 and JOINED mostly present single-ligand entries (≈ 70%). bMOAD_SUB_ (46%) and CHEN11 (58%) present more similar distributions to LIGYSIS, where 54% of the protein chains present more than one binding site. This percentage decreases for LIGYSIS_NI_ (38%) as ion sites are removed. HOLO4K presents the highest proportion (62%) of multi-ligand entries. Both HOLO4K and COACH420 are based on asymmetric units, and not biological assemblies. For HOLO4K, 1811 (40%) of structures present different numbers of chains between the asymmetric and biological units. This is even more frequent in COACH420: 234 (56%). Moreover, multimeric complexes might present the same protein–ligand interface repeated across the copies of the complex (Fig. 1). Considering predictions of these interfaces as independent can lead to an overestimate of the performance of a predictor. Regarding the number of chains interacting with a given ligand (Fig. 3B), CHEN11, COACH420 and JOINED present the smaller fraction of multimeric protein–ligand interactions: 3, 6 and 8%, respectively, whereas SC6K presents the highest (44%). The rest of the methods range between 20 and 30%. There are no striking differences regarding the size of the interacting protein chains, represented by the number of residues (Fig. 3C).

**Table 2:** Summary statistics of the different datasets analysed in this study

Dataset	Type	# Structures	# Sites	# Ligands	Overlap (%)	Methods
LIGYSIS	NEW	3448	8244	**65,116**^**+**^	–	–
LIGYSIS_NI_	NEW	2275	4572	38,595	–	–
sc-PDB_FULL_	TRAIN	**17,594**^**+**^	**17,594**^**+**^	17,594	**801**^**−**^** (9.7)**	VN-EGNN, GrASP, PUResNet, DeepPocket
bMOAD_SUB_	TRAIN	5899	11,184	11,184	606 (7.6)	IF-SitePred
CHEN11	TRAIN	**244**^**−**^	**479**^**−**^	**479**^**−**^	**40**^**+**^** (0.5)**	PRANK, P2Rank
PDBbind_REF_	TEST	5316	5316	5316	310 (3.8)	VN-EGNN
SC6K	TEST	6147	6147	6147	259 (3.1)	DeepPocket
HOLO4K	TEST	4009	10,175	10,175	207 (2.5)	ALL*
COACH420	TEST	413	624	624	41 (0.5)	VN-EGNN, GrASP, DeepPocket, P2Rank, PUResNet
JOINED	TEST	557	752	752	110 (1.3)	PRANK

LIGYSIS is our reference dataset, LIGYSIS_NI_ is a subset with no ion (NI) ligand binding sites, sc-PDB_FULL_, bMOAD_SUB_ and CHEN11 constitute the training datasets, whereas PDBbind_REF_, SC6K, HOLO4K, COACH420 and JOINED represent test sets. # Structures, # Sites and # Ligands represent the number of PDB structures, ligand sites and total number of ligands for each dataset. Note that for LIGYSIS and LIGYSIS_NI_, 3448 and 2775, are the number of human structural segments considered, each represented by a single chain. For each segment, all biologically relevant ligand-binding structures were considered: *N* = 23,321 (LIGYSIS) and *N* = 19,012 (LIGYSIS_NI_). The number of ligands, or protein–ligand complexes, is not equal to the number of sites for LIGYSIS, as data from multiple structures of the same protein are aggregated into unique sites, i.e., a LIGYSIS site often includes multiple ligands. Overlap is the number of LIGYSIS binding sites represented by at least one protein–ligand complex for a given dataset. Percentage relative to LIGYSIS also reported. Methods represents the ligand site predictors that use these datasets for training or test. Only the original version of each dataset is considered in the analysis, e.g., HOLO4K is analysed, but not HOLO4K_Mlig_, nor HOLO4K_Mlig+_ HAP, or HAP-small. The same goes for Mlig, Mlig+ versions of COACH420, sc-PDB_SUB_ and sc-PDB_RICH_. ALL* represents all the methods compared in this work except for PRANK, fpocket, PocketFinder^+^, Ligsite^+^ and Surfnet^+^. For # Structures, # Sites and # Ligands, highest values are indicated with “^**+**^” bold superscript and lowest with “^**−**^”. This is the other way around for Overlap

Figure 3D represents the ligand type composition of the datasets. Non-polymer ligands dominate all datasets (> 66%), and the proportion of peptides and nucleic acids differ across datasets, with JOINED and LIGYSIS presenting fewer ligands of these types (0.9% and 1.6%). sc-PDB_FULL_ and SC6K are depleted in saccharides (< 1%). Figure 3E depicts the difference in the number of atoms of the ligands in each dataset. LIGYSIS is of course different, due to its ion ligand content, however, there is no difference between LIGYSIS_NI_ and the other datasets. Figure 3F conveys the difference in the number of ligand-interacting residues. LIGYSIS has the largest proportion of small sites, 1–10 residues (56%). This is directly related to the prominent ion component, and the frequency decreases when ions are removed (LIGYSIS_NI_: 36%). CHEN11, COACH420, JOINED and HOLO4K are more similar to LIGYSIS_NI_, whereas sc-PDB_FULL_, PDBbind_REF_ and SC6K are clearly different and present almost exclusively large sites, larger than 20 amino acids (> 90%). Figure 3G explores the ligand diversity on each dataset by showing the top-5 most frequent ligands per dataset, the proportion of the total number of ligands they represent, as well as Shannon’s entropy *H′*. Shannon’s entropy is a measure of diversity. Larger numbers indicate a more evenly spread distribution of a larger number of different molecules, whereas small numbers indicate higher frequency of a few ligands. Whilst four out of the top-5 ligands of LIGYSIS are ions (Zn^+2^, Ca^+2^, Mg^+2^, Mn^+2^) and represent 19.2% of all ligands, its diverse composition is comparable to that of PDBbind_REF_. Without ions, LIGYSIS_NI_ is the most diverse dataset with *H′* = 8.8 and its top-5 ligands only covering 5.3% of all ligands in the set. SC6K is the least diverse with its top-5 most frequent ligands covering 33% of all ligands. All datasets except for LIGYSIS, LIGYSIS_NI_, and PDBbind_REF_ are dominated by co-factor ligands, such as flavin-adenine dinucleotide (FAD), nicotinamide-adenine dinucleotide (NAD), and haem (HEM), or energy carrier molecules such as adenine tri-, di- and monophosphate (ATP, ADP, AMP). Short peptides (< 10 aas) are the most common ligands in PDBbind_REF_ (5%), and energy carriers represent < 2% of the top-5 ligands. Cholesterol, mannose and fucose are some of the most common ligands in LIGYSIS_NI_. For all further analysis, including characterisation of binding pockets and performance evaluation of the prediction methods, LIGYSIS, i.e., *including ions*, was utilised. This was done to have a larger benchmark set and to challenge the methods as they have not been trained on such sites.

### Characterisation of binding pockets

**Fig. 4:**
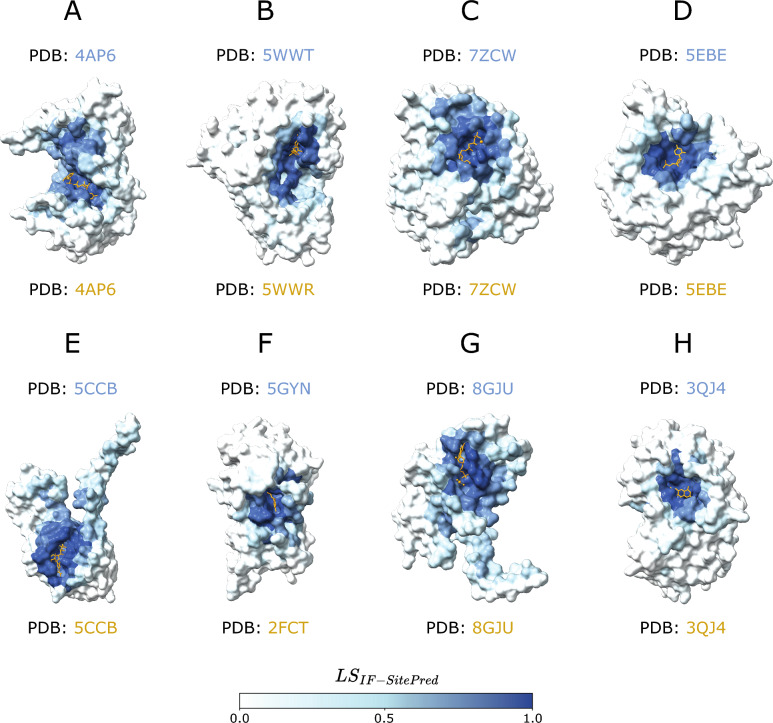
IF-SitePred “missed” predictions. Eight examples of human protein chains where IF-SitePred does not report any predicted ligand binding sites. Predictions are made on ligand-stripped chains. Ligand molecules, in orange, are superposed to illustrate how the ligandability scores recapitulate the observed binding site. These are protein representative chains and ligand molecules might not be observed in the same entry. **A** GDP-fucose protein *O*-fucosyltransferase 2, Q9Y2G5, with GDP-L-fucose (GFB) superimposed (PDB: 4AP6) [76]; **B** tRNA (cytosine(72)-C(5))-methyltransferase NSUN6, Q8TEA1, (PDB: 5WWT) [77] with superposed sinefungin (SFG) (PDB: 5WWR); **C** tubulin beta-2B chain, Q9BVA1, with phosphomethylphosphonic acid guanylate ester (G2P) (PDB: 7ZCW) [78]; **D** cyclic GMP-AMP phosphodiesterase SMPDL3A, Q92484, with cytidine-5'-monophosphate (C5P) (PDB: 5EBE) [79]; **E** tRNA (adenine(58)-*N*(1))-methyltransferase catalytic subunit, Q96FX7, with SAH (PDB: 5CCB) [80]; **F** chronophin, Q96GD0, (PDB: 5GYN) [81] with PLP (PDB: 2FCT) [82]. **G** Mitochondrial methylmalonic aciduria type A protein, Q8IVH4, with GDP (PDB: 8GJU) [83]; **H** renalase, Q5VYX0, (PDB: 3QJ4) with FAD [84]. Residues are coloured based on the ligandability score calculated by averaging the probabilities predicted by each of the 40 IF-SitePred prediction models. This is a score (Eq. 13) ranging 0–1 which is indicative of the likelihood of a given residue binding a ligand (see “Methods”). Clear pockets can be observed formed by residues with high ligandability scores (darker blue colour), which agree with the sites where ligands bind. These “missed” pockets contribute to the lower recall of IF-SitePred and strongly suggest that a more permissive ligand-binding residue selection threshold (currently *all* 40 models) or a different clustering scheme might be able to capture these predictions and increase the recall of this method

After removing representative chains with missing residue mappings to UniProt, the final LIGYSIS set which was employed for the benchmark of the methods comprises 2775 protein chains. All methods were executed with default parameters. P2Rank_CONS_ represents predictions by P2Rank with an extra feature of amino acid conservation and DeepPocket_SEG_ are the pockets extracted by the segmentation module of DeepPocket (see “Methods”). Not all methods predict pockets on all the chains. VN-EGNN, GrASP, fpocket, PocketFinder^+^, Ligsite^+^, and Surfnet^+^ predict in > 99% of the chains, P2Rank_CONS_ on 93%, followed by P2Rank on 86%, PUResNet and DeepPocket_SEG_ (85%), and finally IF-SitePred only predicts pockets on 75% of the chains. PUResNet, DeepPocket, P2Rank_CONS_ and P2Rank often don’t predict on smaller proteins (< 100 amino acids) as well as non-globular or elongated proteins, representing 60–80% of proteins with no predicted pockets. However, for IF-SitePred larger globular proteins represent ≈ 50% of all proteins where this method fails to predict a pocket (Supplementary Figure 1). Predicted residue ligandability scores for P2Rank_CONS_, P2Rank and IF-SitePred (which we derived in this work), were examined for the proteins with no predicted pockets. Figure 4 illustrates 8 examples of proteins where residues with IF-SitePred (Eq. 13) high ligandability scores cluster in space into clear binding sites that are not reported as predictions by this method. This suggests that IF-SitePred is too strict in selecting only those residues predicted as ligand-binding by all 40 models. The cloud point selection and clustering approach or threshold in this method may also play a role in this.

Table 3 summarises the ligand site characterisation analysis. fpocket predicts the most sites out of all the methods, with 57,859, followed by IF-SitePred (44,948), DeepPocket_SEG_ (21,718), VN-EGNN (13,582), P2Rank (12,412), P2Rank_CONS_ (10,180), Surfnet^+^ (9043), PocketFinder^+^ (8913), Ligsite^+^ (6903), GrASP (4694) and PUResNet, which predicts fewest sites (2621). LIGYSIS defines 6882 binding sites from experimental data. Relative to LIGYSIS, the prediction methods have ratios of predicted/defined sites ranging from 8.4 (fpocket) to 0.4 (PUResNet) with P2Rank_CONS_ in the middle, predicting 1.5 pockets per observed reference site. IF-SitePred, DeepPocket_SEG_ as well as P2Rank and fpocket predict more pockets on larger protein chains, whereas the rest of methods do not (Supplementary Note 1 and Supplementary Figure 2). This effect is most clear with fpocket, which predicts 350 pockets for chain A of PDB: 7SUD [85], a structure of the DNA-dependent protein kinase catalytic subunit, DNPK1, (P78527) with 3736 amino acid residues. In contrast, VN-EGNN, which initially places *K* = 8 virtual nodes, results in a maximum of 8 predicted pockets, regardless of protein chain size, and PUResNet predicts a single pocket in 90% of the protein chains.

**Table 3:** Ligand site characterisation summary

Method	Coverage (%)	# Total pockets	# Pockets per protein	$R_{g}$(Å)	MCD (Å)	MRO	Redundancy (%)
LIGYSIS (reference)	2775	6882	1, 1, 27	5.9	14.1	0	2.3
(**d**) VN-EGNN	2764 (99.6)	13,582 (×2.0)	1, 5, 7	5.9	1.1*	0.85*	66.8*
(**d**) IF-SitePred	2075 (74.8*)	44,948 (×6.5)	1, 20*, 129	5.9	3.4	0.55	49.5
(**d**) GrASP	2771 (99.9)	4694 (×0.7)	1, 1, 12	7.9	21.4	0	0.0
(**d**) PUResNet	2360 (85.1)	2621 (×0.4)	1, 1, 4	8.1	27.0	0	0.0
(**d**) DeepPocket_SEG_	2349 (84.7)	21,718 (×3.2)	1, 6, 196	7.7	4.6	0.4	31.1
(**d**) P2Rank_CONS_	2759 (92.9)	12,412(×1.8)	1, 3, 57	7.1	13.9	0.05	0.7
(**d**) P2Rank	2402 (86.6)	10,180 (×1.5)	1, 3, 85	7.1	13.8	0.05	0.6
(**d**) fpocket	2759 (99.4)	57,859 (×8.4*)	1, 17, 349*	6.3	9.7	0.15	0.7
(**d**) PocketFinder^+^	2775 (100)	8913 (×1.3)	1, 3, 23	8.6	18.7	0.05	0.0
(**d**) Ligsite^+^	2775 (100)	6903 (×1.0)	1, 2, 12	9.1*	16.7	0.09	0.0
(**d**) Surfnet^+^	2775 (100)	9043 (×1.3)	1, 3, 40	8.4	17.2	0.07	0.0

LIGYSIS is not a ligand binding site predictor, but a reference dataset curated from experimentally determined structures of biologically relevant protein–ligand complexes. These predictions result from the default prediction of the methods, indicated by (**d**) preceding method names. Coverage represents the number of protein chains for which the different methods return at least one prediction. Percentage is relative to LIGYSIS protein chains. VN-EGNN failed with an error for PDB: 6BCU chain: A [86]. The rest of the methods ran successfully for all protein chains; # total pockets and ratio of predicted pockets by reference pockets in parenthesis, e.g., for each LIGYSIS site, fpocket predicts on average 8.4 pockets; minimum, median and maximum number of pockets per protein; Median pocket radius of gyration *R*_*g*_ (Å); median minimum centroid distance (MCD) (Å) for all pockets. For proteins where multiple pockets are predicted, MCD represents the distance to the closest pocket centroid for each of the different predicted pockets within a protein. This is a measure of how close predicted pockets are to each other; maximum residue overlap (MRO). For a given pocket, MRO is the maximum residue overlap with other pockets’ residues within a protein. MRO is a measure of how similar, in terms of shared residues, the predicted pockets are (see “Methods” for detailed explanation). For example, the median overlap between VN-EGNN predicted pockets is 85%. Redundancy represents the percentage of predicted pockets that are redundant, i.e., the closest pocket centroid is within 5 Å, or overlap is at least 3/4 (≥ 75%) residues. This is the case for 67% of VN-EGNN pockets and 0% for GrASP pockets. Bold font and “*” indicate the most extreme values within each column

**Fig. 5:**
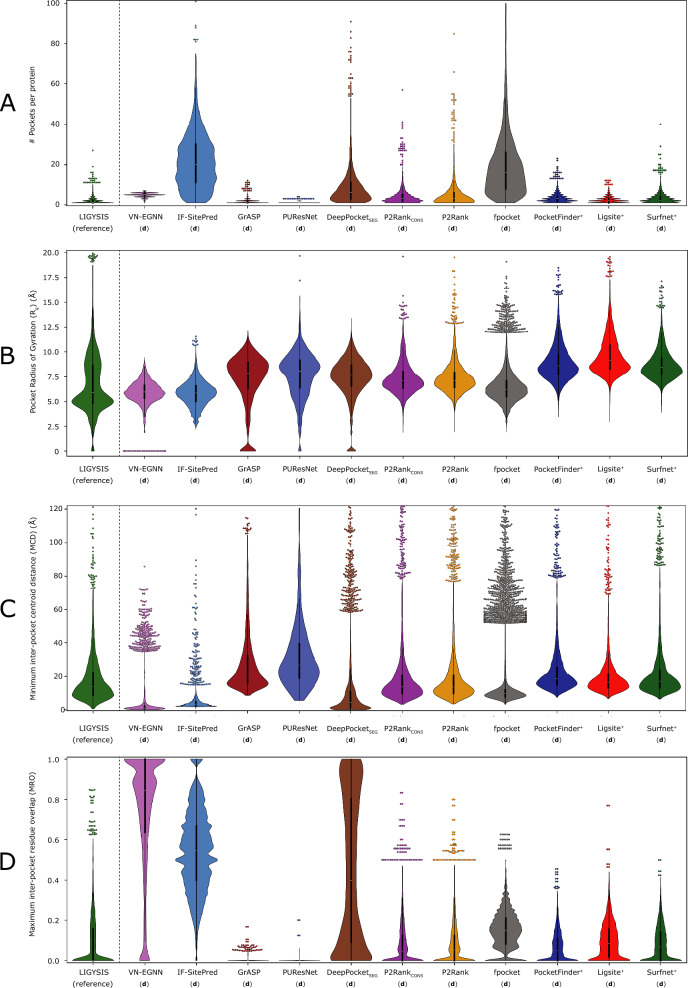
Binding pocket characterisation. Ligand binding site predictions by eleven methods with default parameters (**d**) are compared to the LIGYSIS reference dataset. A black dashed line separates LIGYSIS from the eleven ligand binding site predictors. Predictions by DeepPocket_RESC_ and fpocket_PRANK_ are not included in this analysis as they are re-scored and re-ranked fpocket predictions and their plotting would be redundant. Data points farther than four standard deviations (SD) from the mean are considered outliers. The limit of the *Y* axis is the maximum *non-outlier* value plus a buffer value. This way, only the most extreme outliers are hidden, which maximises visual interpretation of the data whilst minimising the number of data points not shown. Within the violin plots are box plots representing the underlying distribution. Line represents the median, box contains the interquartile range (IQR), and whiskers extend to 1.5 × IQR. **A** Number of pockets per protein; **B** pocket radius of gyration *R*_*g*_ (Å); **C** minimum inter-pocket centroid distance (MCD) (Å). This is a measure of how close predicted pockets are to each other within a protein; **D** Maximum inter-pocket residue overlap (MRO). Residue overlap was calculated as Jaccard Index. This is a measure of how much pockets overlap in terms of binding residues

Figure 5 represents how the eleven unique sets of ligand site predictions compare to each other as well to LIGYSIS, which *defines* ligand sites from experimentally determined biologically relevant protein–ligand complexes. There are eleven unique sets of predictions since DeepPocket_RESC_ and fpocket_PRANK_ do not predict, but re-score and re-rank fpocket predictions. However, DeepPocket_SEG_ predictions are different as new pocket shapes are extracted by the CNN segmentation module. Figure 5A shows how VN-EGNN, GrASP and PUResNet differ from the other methods with a maximum of 7, 12 and 4 predicted pockets, respectively. PocketFinder^+^, Ligsite^+^ and Surfnet^+^ present narrow distributions like LIGYSIS and with medians of 1–3 pockets per protein. P2Rank_CONS_ and P2Rank also present a median of 3 pockets per protein but display wider distributions as they can predict up to 60, and 80 pockets per protein, respectively. Overall, P2Rank_CONS_ predicts fewer pockets than P2Rank. DeepPocket_SEG_, fpocket, and IF-SitePred follow with a median of 6, 17, and 20 pockets. The difference in number of pockets between DeepPocket_SEG_ and DeepPocket_RESC_ or fpocket is because 60% of fpocket candidates are not extracted by the CNN segmentation module implemented in DeepPocket.

Figure 5B shows the distribution of pocket radius of gyration, *R*_*g*_. VN-EGNN and IF-SitePred differ from the rest of methods with narrow distributions and medians around 6 Å. These two methods do not report pocket residues. Instead, they were obtained using a distance threshold of 6 Å from the centroid, for VN-EGNN, and cloud points, for IF-SitePred. This is reflected by examining the percentage of pockets with *R*_*g*_ > 10 Å which is 0% and 0.1% for VN-EGNN and IF-SitePred. This is a striking difference compared to LIGYSIS and the other methods: 1.8% (fpocket), 4.8% (P2Rank), 5.7% (DeepPocket_SEG_), 6.4% (GrASP), 6.5% (P2Rank_CONS_), 11.6% (PUResNet), 12.6% (LIGYSIS), 16% (Surfnet^+^), 21.4% (PocketFinder^+^) and 33.5% (Ligsite^+^). The latter three predict the sites with largest median *R*_*g*_ ≈ 9 Å. VN-EGNN, GrASP, PUResNet and DeepPocket_SEG_ predict sites with *R*_*g*_ = 0 Å. This is rather infrequent (7.8% GrASP) and < 3% for the other three. These examples correspond to *singletons*, i.e., pockets formed by only one amino acid.

Figure 5C illustrates how close predicted sites are to each other within a protein chain. Pairwise distances between the centroids of all ligand site pairs for a protein are calculated, and for each site, the minimum distance is taken. Sites predicted by VN-EGNN, IF-SitePred and DeepPocket_SEG_ are very close to each other, with median distances $(\tilde{d})$ of 1.1, 3.4 and 4.6 Å, respectively. fpocket follows with $\tilde{d}$ = 9.7 Å. The rest of the methods, and LIGYSIS, present median distances ranging between 13 and 18 Å (LIGYSIS, P2Rank_CONS_, P2Rank, PocketFinder^+^, Ligsite^+^, Surfnet^+^), and finally GrASP ($\tilde{d}$ = 21.7 Å) and PUResNet ($\tilde{d}$ = 27 Å). Both versions of P2Rank present the most similar distribution to what is observed on LIGYSIS.

Figure 5D depicts the overlap existing between residues that form the predicted pockets within a protein. All pairwise overlaps, i.e., Jaccard Index, are calculated between pockets in a chain, and for each pocket, the maximum is taken. This is a measure of how much predicted pockets overlap. This is directly related to how close pockets are, and so VN-EGNN, IF-SitePred and DeepPocket_SEG_ present very high overlaps $\tilde{o}$ = 0.85, $\tilde{o}$ = 0.55 and $\tilde{o}$ = 0.40, respectively. fpocket follows with $\tilde{o}$ = 0.15, Ligsite^+^ ($\tilde{o}$ = 0.09), Surfnet^+^ ($\tilde{o}$ = 0.07), P2Rank_CONS_, P2Rank and PocketFinder^+^ ($\tilde{o}$ = 0.05), and finally LIGYSIS, GrASP and PUResNet with $\tilde{o}$ = 0.0. GrASP is the only out of the thirteen methods presented here that clusters atoms directly, and as a result, overlap between pockets is minimised. Other methods cluster cloud points (IF-SitePred), SAS points (P2Ranks) voxels (PUResNet, DeepPocket_SEG_), alpha spheres (fpocket), or grid points (PocketFinder^+^, Ligsite^+^, Surfnet^+^) but not residues, resulting consequently in higher overlapping.

Proximity in space between predicted sites as well as residue overlap are indicators of redundant ligand binding site prediction, i.e., duplicate predictions of a unique observed ligand site. This is the case for VN-EGNN, IF-SitePred and DeepPocket_SEG_. This phenomenon can negatively impact the precision and recall of these methods. Accordingly, correcting for redundancy should have a significant impact on the performance of these methods. In contrast, GrASP and PUResNet which predict a small number of pockets show low proximity and overlap of predicted sites and so redundancy is not an issue.

### Evaluation of predictive performance

#### Pocket level evaluation

The ideal ligand binding site predictor would have a high precision, i.e., most of the predictions it makes are correct, whilst maintaining a high recall, i.e., recapitulating most of the observed sites. Moreover, the ideal predictor returns predictions that are non-redundant, i.e., it does not predict the same pocket multiple times. Additionally, pockets are ranked in a systematic manner according to a strong and meaningful pocket scoring scheme which captures well the nature of existing ligand binding sites and therefore ranks the predicted pockets from more likely (high score, top) to least likely (low score, bottom). A good predictor would also perform well at the residue level. This means it is able of capturing the likelihood for a residue to bind a ligand. This can be done by means of a residue ligandability score, which additionally might highlight key residues, the more “ligandable” within a binding site. Ligand site prediction methods were benchmarked with these criteria in mind. The following results concern the original version of the predictors. The non-redundant and re-scored variants of the methods will be discussed in a later section.

**Fig. 6:**
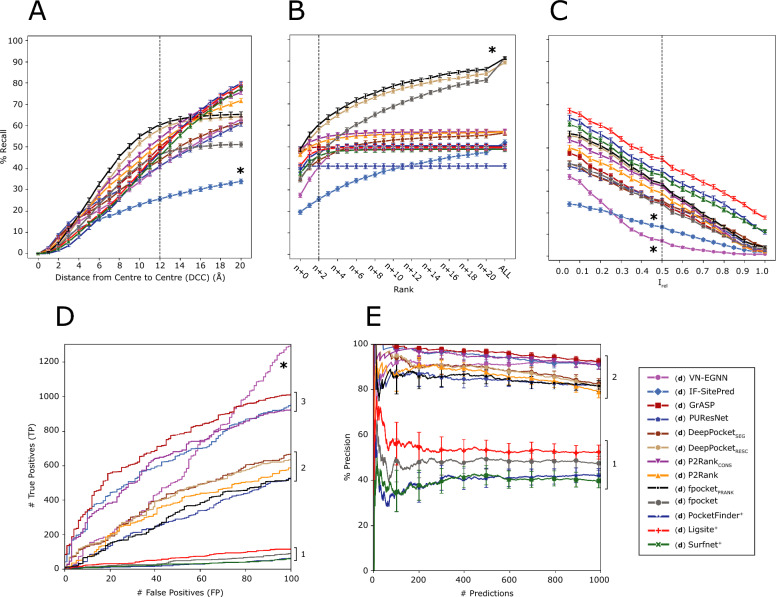
Ligand binding site prediction benchmark at the pocket level. These curves correspond to the default predictions of the thirteen methods, indicated by (**d**) preceding their names. **A** Recall, percentage of observed sites that are correctly predicted by a method within the top-*N*+2 predictions; **B** recall using DCC = 12 Å but considering increasing rank thresholds, i.e., top-*N*, *N*+1, *N*+2, etc. *ALL* represents the maximum recall of a method, obtained by considering all predictions, regardless of their rank or score; **C** recall curve of top-*N*+2 predictions using *I*_*rel*_ as a criterion; **D** ROC100 curve (cumulative TP against cumulative FP until 100 FP are reached); **E** precision curve for the top-1000 predictions of each method across the LIGYSIS dataset, *Precision*_*1K*_. Error bars represent 95% CI of the recall (**A**–**C**) and precision (**E**), which is 100 × proportion. Numbers at the right of the panels indicate groups or blocks of methods that perform similarly for each metric. Asterisks (*) indicate outlier methods, or methods that perform very differently than the rest

Figure 6A illustrates the recall curve for top-*N*+2 pockets for each method, where *N* is the number of observed sites for a target protein. Reported recall is obtained using DCC = 12 Å. Re-scored fpocket predictions by PRANK (fpocket_PRANK_) and DeepPocket (DeepPocket_RESC_) yield the highest recall with 60.4% and 58.1%, respectively, closely followed by, P2Rank_CONS_ (53.9%) and P2Rank (51.9%). The rest of the methods present recall < 50% with PUResNet, VN-EGNN and IF-SitePred with lowest recall of 41.1%, 40.9% and 25.7%, respectively (Table 4). Figure 6B, shows the recall curve considering different top-*N*+*X* predictions. Most methods reach a plateau by top-*N*+5, as they do not predict that many pockets. However, methods that predict more pockets per protein, such as IF-SitePred, or fpocket, fpocket_PRANK_, DeepPocket_SEG_ and DeepPocket_RESC_, which take fpocket predictions as a base, increase their recall as more predictions are considered. fpocket, fpocket_PRANK_ and DeepPocket_RESC_ reach a maximum recall of ≈ 90% when *all* predictions are considered, regardless of rank. Most other methods present a maximum recall of ≈ 50–60% and PUResNet presents the lowest maximum recall of 41%. Figure 6C illustrates the top-*N*+2 recall curve if residue overlap, *I*_*rel*_, was used instead of DCC as a criterion. In this case, Ligsite^+^, PocketFinder^+^, and Surfnet^+^ come on top with recall ≈ 45% at *I*_*rel*_ ≥ 0.5. This is explained by their prediction of massive cavities, that while often fully contain or overlap with the observed pocket, do not meet the DCC criterion, as their centroids are farther than 12 Å from the observed site.

**Table 4:** Pocket level evaluation summary table

Method	% Recall_top-*N*_	% Recall_top-*N*+2_	% Recall_max_	% Precision_1K_	# TP_100 FP_	% RRO	% RVO
(**d**) VN-EGNN	27.5 (#11)	40.9 (#12)	49.3 (#10)	**92.5**^**+**^** (#1)**	**1301**^**+**^** (#1)**	**32.8**^**−**^** (#12)**	**27.6**^**−**^** (#11)**
(**d**) IF-SitePred	**19.8**^**−**^** (#12)**	**25.7**^**−**^** (#13)**	52.1 (#6)	91.0 (#2)	961 (#3)	46.5 (#11)	40.4 (#9)
(**d**) GrASP	48.0 (#2)	49.9 (#5)	50.0 (#8)	**92.5**^**+**^** (#1)**	1017 (#2)	54.5 (#7)	59.8 (#6)
(**d**) PUResNet	40.6 (#6)	41.1 (#11)	**41.1**^**−**^** (#12)**	81.6 (#6)	534 (#8)	61.0 (#4)	63.9 (#4)
(**d**) DeepPocket_SEG_	35.4 (#10)	43.8 (#10)	56.5 (#5)	82.6 (#4)	670 (#5)	57.5 (#5)	60.3 (#5)
(**d**) DeepPocket_RESC_	46.6 (#4)	58.1 (#2)	89.3 (#2)	81.7 (#5)	637 (#6)	53.1 (#9)	38.2 (#10)
(**d**) P2Rank_CONS_	**48.8**^**+**^** (#1)**	53.9 (#3)	57.0 (#4)	90.7 (#3)	932 (#4)	56.4 (#6)	43.8 (#8)
(**d**) P2Rank	46.7 (#3)	51.9 (#4)	57.0 (#3)	79.2 (#7)	586 (#7)	54.4 (#8)	58.2 (#7)
(**d**) fpocket_PRANK_	**48.8**^**+**^** (#1)**	**60.4**^**+**^** (#1)**	**91.3**^**+**^** (#1)**	81.7 (#5)	526 (#9)	52.6 (#10)	38.2 (#10)
(**d**) fpocket	38.8 (#8)	46.5 (#8)	**91.3**^**+**^** (#1)**	47.3 (#9)	94 (#11)	52.6 (#10)	38.2 (#10)
(**d**) PocketFinder^+^	39.2 (#7)	47.8 (#7)	50.5 (#7)	42.0 (#10)	64 (#12)	72.3 (#2)	75.9 (#2)
(**d**) Ligsite^+^	41.3 (#5)	48.4 (#6)	49.7 (#9)	52.3 (#8)	115 (#10)	**77.6**^**+**^** (#1)**	**77.0**^**+**^** (#1)**
(**d**) Surfnet^+^	37.7 (#9)	45.8 (#9)	48.9 (#11)	**39.5**^**−**^** (#11)**	**61**^**−**^** (#13)**	71.7 (#3)	72.0 (#3)

These metrics correspond to the default modes of the thirteen methods covered in this work, indicated by (**d**) preceding the methods’ names. % Recall for each method considering top-*N*, *N*+2 and *all* predictions (max) without taking rank into consideration, i.e., maximum recall. Precision of the method for the top-1000 scored predictions. Number of TP reached for the first 100 FP (# TP_100FP_). Mean % relative residue overlap (RRO) for those sites correctly predicted and % relative volume overlap (RVO) only for correctly predicted sites that have a volume, i.e., are pockets or cavities, and not exposed sites, which don’t have a volume. These last two metrics represent the overlap in residues and volume relative to the observed site. See “Methods” section for definitions of RRO and RVO. Within each cell, the numbers following a dash (#) indicate the rank of each method according to the metric in the column. Bold font indicates the best (“^**+**^”) and worst (“^**−**^”) performing methods for each metric

Figure 6D represents the cumulative number of TP against FP when predictions across the proteins in the reference dataset are sorted by score. This shows how effective the scoring scheme of each method is in ranking their predictions to reflect the nature of ligand binding sites. At 100 FP the #TP fall into three different blocks and one outlier: Ligsite^+^, fpocket, Surfnet^+^ and PocketFinder^+^ are at the bottom with # TP ∈ (60, 120). Secondly, fpocket_PRANK_, DeepPocket_SEG_, DeepPocket_RESC_, P2Rank, and PUResNet follow with # TP ∈ (530, 670). Re-scoring fpocket predictions with PRANK or DeepPocket results in up to + 500 TP. GrASP, IF-SitePred and P2Rank_CONS_, present a high # TP ranging 900–1000 at 100 FP. Finally, VN-EGNN sits at the top with 1301 TP. However, this number is not representative, as the # TP are inflated due to the redundancy in the predictions of VN-EGNN. This is the same for IF-SitePred and DeepPocket_SEG_. Redundant correct predictions of the same pocket will count as multiple TPs, whereas they should only count as 1 TP. Newer methods, e.g., GrASP, P2Rank_CONS_, and VN-EGNN, and IF-SitePred, despite redundancy in their prediction for the latter two, are better at ranking their predicted pockets, presenting up to 1000 more TP for 100 FP than earlier methods. This means their scoring schemes are significantly better at capturing the essence of a ligand binding site. Including evolutionary conservation in P2Rank (P2Rank_CONS_) predictions results in an increase of + 346 TP relative to default P2Rank, indicating that the fewer predicted pockets, and their scores are a more faithful representation of the observed LIGYSIS dataset.

**Fig. 7:**
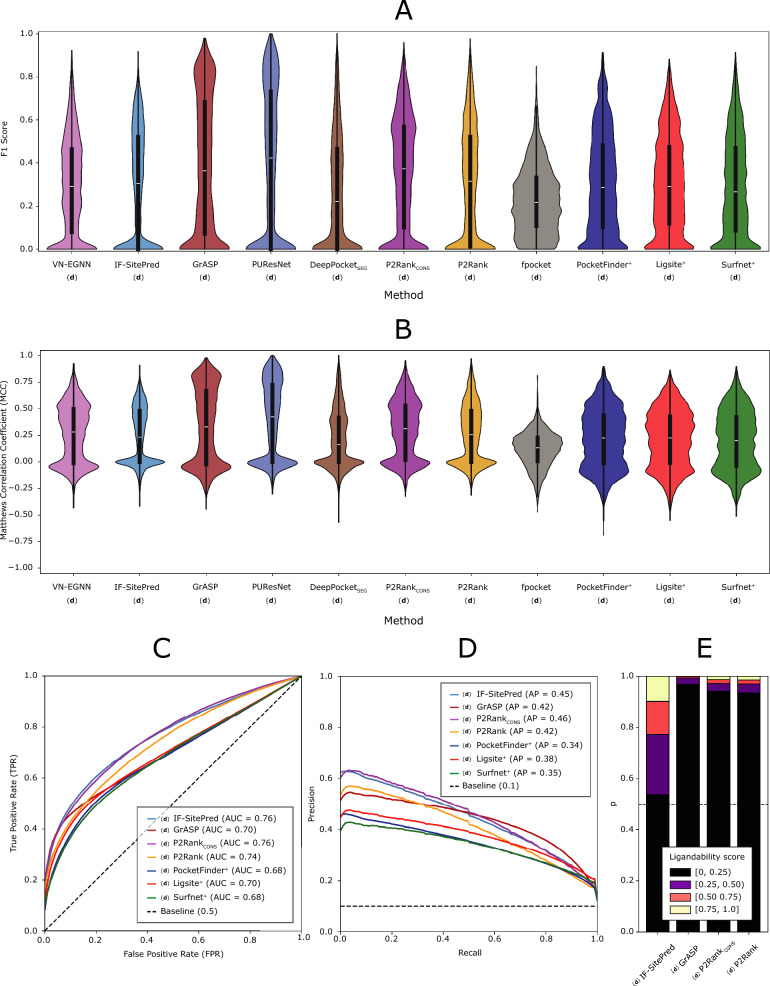
Ligand binding site prediction benchmark at the residue level. DeepPocket_RESC_ predictions are not included in the F1 and MCC analyses as these are re-scored and re-ranked fpocket predictions and the results would be the same as fpocket’s. **A** F1 score distributions; **B** MCC distributions. In both panels, each data point in the distribution corresponds to the score obtained from all residues in a protein chain; **C** mean ROC curve for methods that report a residue score. Dashed line represents the baseline, 1 FP for each TP, i.e., diagonal and AUC = 0.5; **D** mean PR curve. Dashed line represents the baseline, i.e., proportion of observed binding residues = 0.1; Mean ROC and PR curves are calculated by averaging the curves of the 2775 LIGYSIS protein chains. **E** Distribution of residue ligandability scores for IF-SitePred, GrASP and P2Ranks. PocketFinder^+^, Ligsite^+^ and Surfnet^+^ are not included as their scores do not range 0–1, and a small number of scores can reach values > 25. (**d**) indicates that these predictions originate from the original methods run with default parameters

Figure 6E provides insight into the precision of the methods by examining how this metric changes as more predictions are considered. In the same manner as for Fig. 7D, predictions across proteins in the LIGYSIS dataset are sorted and cumulative precision is plotted for the top-1000 scoring predictions. Methods group into two clear blocks. Newer (machine learning-based) methods VN-EGNN, GrASP, IF-SitePred, P2Rank_CONS_, DeepPocket_SEG_, fpocket_PRANK_, DeepPocket_RESC_ and PUResNet present a Precision_1K_ of 80–95%. Earlier (geometry/energy-based) methods Ligsite^+^, fpocket, PocketFinder^+^ and Surfnet^+^ present lower Precision_1K_ of 40–50%. DeepPocket_RESC_ and fpocket_PRANK_ take fpocket (geometry-based) predictions as a starting point and achieve much higher # TP_100FP_ (+ 500) as well as Precision_1K_ (+ 30%). This is further evidence that performance can be boosted with a solid scoring scheme and agrees with previous studies [37, 38, 40, 87].

Table 4 summarises these results and shows the mean relative residue overlap (RRO) and relative volume overlap (RVO) which measure how well predicted sites align with observed ones in shape. VN-EGNN and IF-SitePred present the smallest RRO and RVO, but it is important to note that these methods do not report pocket residues and so residues were taken within 6 Å of their centroid, or pocket spheres. PocketFinder^+^, Ligsite^+^, and Surfnet^+^ present the highest RRO and RVO. This is a consequence of the large size of their predicted cavities, that rather than overlap with the observed site, fully contain and are much larger than it. This might not be convenient in the context of pocket finding for drug discovery where more clearly defined drug-like sites might be of interest. GrASP, PUResNet and DeepPocket_SEG_ present high values of RRO ≈ 60% and RVO ≈ 60% whilst presenting a size distribution more like LIGYSIS (Fig. 5B) and provide the best representation of the observed sites in terms of shape similarity.

#### Residue level evaluation

Ligand binding site prediction tools can also be evaluated at the residue level. F1 Score and Matthews correlation coefficient (MCC) are utilised to do so. For each protein chain, F1 and MCC are calculated, distributions graphed and means reported (Table 5). Binary labels are employed to calculate these scores: 1 if the residue is found in a pocket, and 0 otherwise and compared to the ground truth, i.e., whether a residue binds a ligand in the LIGYSIS set. For VN-EGNN, IF-SitePred, PocketFinder^+^, Ligsite^+^, and Surfnet^+^, which do not report pocket residues (Table 1), pocket residues were obtained by taking those residues within 6 Å of the pocket centroid, cloud points or grid points, respectively. DeepPocket_RESC_ is not considered for this analysis as the predictions are re-scored and re-ranked fpocket predictions.

**Table 5:** Residue level evaluation summary table

Method	F1	MCC	AUC	AP
(**d**) VN-EGNN	0.29 (#8)	0.26 (#4)	–	–
(**d**) IF-SitePred	0.29 (#9)	0.24 (#6)	0.76 (#2)	0.45 (#2)
(**d**) GrASP	0.39 (#2)	0.34 (#2)	0.70 (#4)	0.42 (#3)
(**d**) PUResNet	**0.41**^**+**^** (#1)**	**0.39**^**+**^** (#1)**	–	–
(**d**) DeepPocket_SEG_	0.27 (#10)	0.21 (#9)	–	–
(**d**) P2Rank_CONS_	0.36 (#3)	0.30 (#3)	**0.76**^**+**^** (#1)**	**0.46**^**+**^** (#1)**
(**d**) P2Rank	0.31 (#4)	0.26 (#5)	0.74 (#3)	0.42 (#3)
(**d**) fpocket	**0.23**^**−**^** (#11)**	**0.12**^**−**^** (#11)**	–	–
(**d**) PocketFinder^+^	0.31 (#5)	0.22 (#7)	0.68 (#6)	**0.34**^**−**^** (#6)**
(**d**) Ligsite^+^	0.31 (#6)	0.21 (#8)	0.70 (#5)	0.38 (#4)
(**d**) Surfnet^+^	0.29 (#7)	0.20 (#10)	**0.68**^**−**^** (#7)**	0.35 (#5)

These results come from default predictions, indicated by (**d**). DeepPocket_RESC_ is not considered in this analysis as their predictions are fpocket’s but re-scored and re-ranked. Ligand binding site prediction benchmark at the residue level was calculated from 2775 protein chains in the LIGYSIS dataset. Mean F1 score, mean Matthews correlation coefficient (MCC), mean ROC area under the curve (AUC) and mean precision recall (PR) curve average precision (AP). Within each cell, the numbers following a dash (#) indicate the rank of each method according to the metric in the column. Bold font indicates the best (“^**+**^”) and worst (“^**−**^”) performing methods for each metric. Pocket binary labels (0, 1) were employed for the calculation of F1 and MCC and obtained from predicted pockets. Residue ligandability scores were employed to calculate ROC/AUC and PR/AP. Reported AUC and AP are means resulting from the average across the 2775 LIGYSIS protein chains. This was not possible for VN-EGNN, PUResNet, DeepPocket_SEG_ and fpocket as these methods do not provide such scores, indicated by a dash (–)

Figure 7A, B illustrate the distributions of the F1 score and MCC for each method on the 2775 protein chains of the final LIGYSIS set. Both metrics agree that PUResNet (F1 = 0.41, MCC = 0.39), GrASP (F1 = 0.39, MCC = 0.33) and P2Rank_CONS_ (F1 = 0.36, MCC = 0.30) are the top-3 performing methods in this task of binary classification into pocket (1) and non-pocket residues (0). fpocket presents the lowest F1 = 0.23 and MCC = 0.12 since it predicts many unobserved pockets (residues) that will count as FP here.

IF-SitePred does not report a residue ligandability score beyond a binary label (0, 1). Nevertheless, in this work, a score was computed by utilising the scores returned by the 40 prediction models of IF-SitePred. These scores range 0–1 and can be averaged as probabilities (Eq. 13). This will now be referenced as IF-SitePred ligandability score. For IF-SitePred, GrASP, P2Rank_CONS_, P2Rank, PocketFinder^+^, Ligsite^+^, and Surfnet^+^, which report a residue level score (beyond a binary label), ROC and PR curves were plotted (Fig. 7C) and mean area under the curve (AUC) and mean average precision (AP) reported. This was not possible for VN-EGNN, PUResNet, DeepPocket_SEG_, DeepPocket_RESC_ and fpocket as they do not report residue ligandability scores. See Supplementary Figure 10 for details on the variation in ROC and AUC across LIGYSIS protein chains. P2Rank_CONS_ and IF-SitePred, with the ligandability score calculated in this work (Eq. 13), present the highest mean AUC = 0.76, closely followed by P2Rank (AUC = 0.74). Surfnet^+^ presents the lowest mean AUC = 0.68. Figure 7D shows the mean PR curves, which agree with ROC AUC and highlight P2Rank_CONS_ as the method with the highest average precision = 0.46, followed by IF-SitePred (with Eq. 13 scoring) (AP = 0.45) and PocketFinder^+^ the lowest with (AP = 0.34). See Supplementary Figure 11 for details on the variation in PR and AP across LIGYSIS protein chains. Figure 7E shows IF-SitePred presenting a different residue ligandability score distribution to GrASP, P2Rank_CONS_, and P2Rank. The IF-SitePred ligandability score, resulting from averaging the scores from the 40 IF-SitePred models, is the most “generous” with ≈ 20% of the residues presenting a score > 0.5, in contrast with GrASP which residue scoring is very strict, *P*(*LS* ≥ 0.5) = 0.75%, and P2Ranks (≈ 3%). This difference, combined with the ROC and PR curves further supports the use of the IF-SitePred ligandability score proposed in this work to define the predicted binding sites in this method. It also suggests that GrASP might benefit from a less strict residue level scoring scheme. PocketFinder^+^, Ligsite^+^, and Surfnet^+^ were not included in this analysis as their scores do not range 0–1 and very high scores (> 25) can be obtained.

#### Improving current methods by redundancy removal and pocket re-scoring

**Fig. 8:**
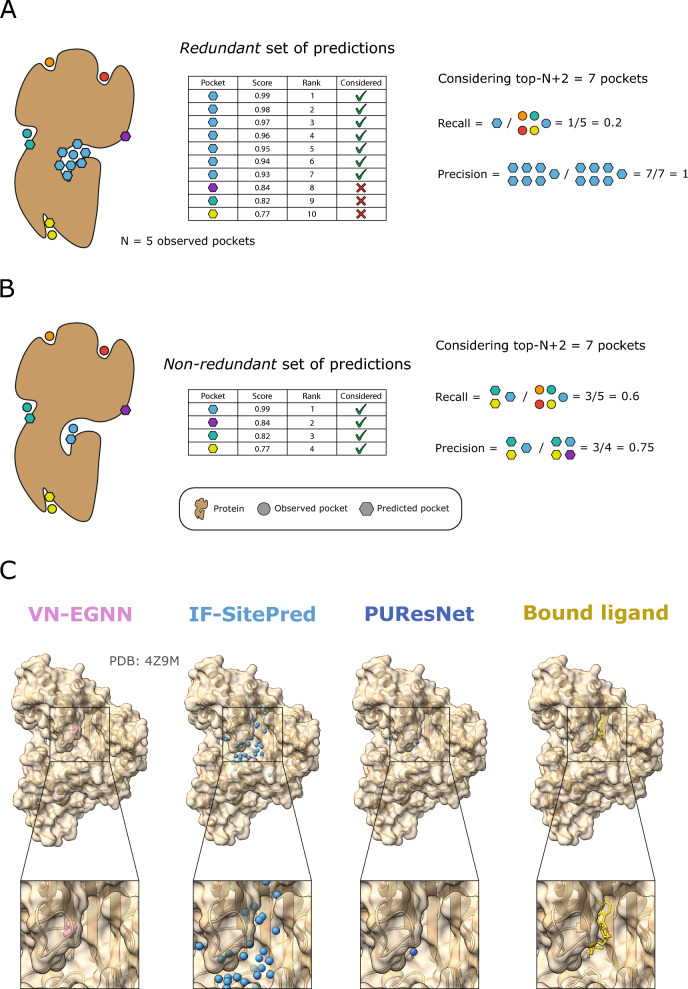
The issue of redundancy in ligand binding site prediction. **A** A set of predictions where 6/10 predictions are redundant, resulting in a low recall (1/5) and inflated precision (7/7) considering the top-*N*+2 predictions; **B** when redundancy is removed, only four predictions remain and recall increases to 3/5 (60%) and precision decreases to 3/4 (75%); **C** predictions by VN-EGNN, IF-SitePred and PUResNet, on chain D of PDB: 4Z9M [88], where ADP binds. For this ADP binding site, VN-EGNN reports 7 predictions, IF-SitePred 33, and PUResNet a single prediction. These three methods correctly predict this site, however, VN-EGNN and IF-SitePred report redundant pocket predictions, which centroids are very close in space (≤ 5 Å) and residues overlap considerably (≥ 0.75)

We define pocket prediction redundancy as the prediction of pockets with centroids very close in space (*d* ≤ 5 Å) or with overlapping residues (*JI* ≥ 0.75). This indicates multiple predictions of the same potential ligand binding site. Most ligand site prediction tools predict not only the location of the pocket by means of a centroid or pocket residues, but also a pocket confidence, and an associated rank among all the predicted pockets. Ligand site predictors tend to be evaluated by considering the top-*N*, or top-*N*+2 ranking pockets, where *N* is the number of observed sites for a given protein. Figure 8A, B illustrates how the redundant prediction of pockets can lead to an overestimation of precision and underestimation of recall (top-*N*+2). Figure 8C showcases human creatine kinase S-type, mitochondrial (PDB: 4Z9M) as an example of this phenomenon, where VN-EGNN and IF-SitePred redundantly predict the same pocket 7 and 33 times, whereas PUResNet returns a single prediction. All three methods correctly predict the site, just the difference is in the number of returned predictions.

Figure 5D shows that prediction redundancy is an issue particularly for VN-EGNN, IF-SitePred, and to a lesser extent DeepPocket_SEG_. To assess the effect that redundancy has on the performance of these methods, non-redundant subsets of predictions were obtained and labelled with the subscript “_NR_”. Redundancy filtering was carried out for each method and adjacent pockets (*d* ≤ 5 Å) *or* sharing more than ¾ of their residues (*JI* ≥ 0.75) were dropped, keeping always the higher scoring pocket. Redundancy (%) was calculated as the proportion of redundant pockets relative to the original total number of pockets. VN-EGNN presents the highest percentage of redundant pockets with 9066/13,582 (67%) redundant pockets, followed by IF-SitePred with 22,232/44,948 (49%), and DeepPocket_SEG_ with 6744/21,718 (31%). For other methods, redundancy was minimal (< 1%).

New ligand binding site prediction methods tend to score their pockets to rank them based on their likelihood of being a real binding site. A robust pocket scoring scheme is helpful to rank predicted pockets in a meaningful order, so pockets at the top are more likely to be real pockets. In other words, a pocket scoring scheme that captures the nature of ligand sites is key to maximising the benchmark performance and usability of these tools. IF-SitePred opts for a simple scheme, using the number of clustered cloud points as the pocket score. Other methods use more sophisticated approaches, such as taking the output of a machine learning model (VN-EGNN, DeepPocket), summing the model scores of atoms, residues or SAS points (GrASP, P2Rank_CONS_, P2Rank) or using multiple pocket-level features (fpocket). Finally, methods like PUResNet, PocketFinder^+^, Ligsite^+^ or Surfnet^+^ do not report a pocket score.

As detailed in “Methods”, re-scored and re-ranked variants of predictions by IF-SitePred were generated. Additionally, pockets were scored and ranked for PUResNet, PocketFinder^+^, Ligsite^+^ and Surfnet^+^. Three different approaches were investigated: (1) sorting by number of pocket amino acids “_AA_” (larger pockets ranking higher); (2) running PRANK to score pockets [37] “_PRANK_”; (3) re-calculating a score with the sum of square of cloud points (IF-SitePred_RESC_) or grid points “_SS_” for PocketFinder^+^, Ligsite^+^ or Surfnet^+^, in a similar manner as done by Krivák et al*.* [37], and later Smith et al*.* [54]. Refer to Supplementary Note 2 and Supplementary Figure 3 for the effect that redundancy and different pocket scoring strategies have on pocket ranking.

**Fig. 9:**
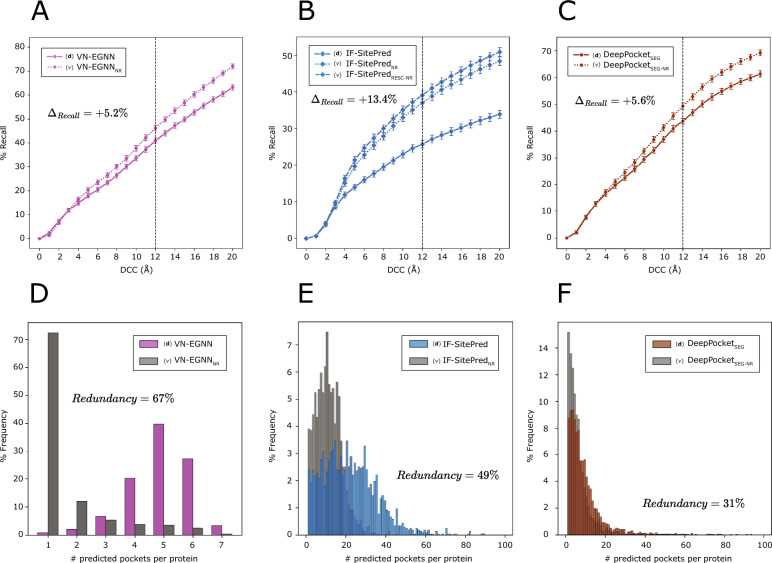
Improvement on the current methods. Solid lines represent top-*N*+2 recall, i.e., proportion of observed ligand binding sites that are correctly identified by each default version for VN-EGNN (**A**), IF-SitePred (**B**) and DeepPocket_SEG_ (**C**). Dashed/dotted lines represent the non-redundant and re-scored (for IF-SitePred) variants of predictions by these methods generated in this work. Removing redundancy increases significantly the recall of VN-EGNN, IF-SitePred and DeepPocket_SEG_ by > 5%. Vertical black dashed lines indicate the threshold used as reference in this work: DCC = 12 Å. Comparison of number of predicted pockets per protein between standard and non-redundant predictions for VN-EGNN (**D**), IF-SitePred (**E**) and DeepPocket_SEG_ (**F**)

In this section, we explore the effect that redundancy removal and pocket scoring/re-scoring have on % Recall (top-*N*+2), % Precision_1K_, and # TP_100FP_. Figure 9 illustrates how removing redundant predictions and re-ranking those remaining resulted in a significant (*p* ≤ 0.05) increase in recall of + 5.2% for VN-EGNN (Fig. 9A), + 13.4% for IF-SitePred (Fig. 9B) and + 5.6% for DeepPocket_SEG_ (Fig. 9C). Re-scoring IF-SitePred predictions using Eq. 13 resulted in a further increase of + 2.1%. After removing redundancy, the number of unique pockets by each method reduces drastically for VN-EGNN (− 67%), IF-SitePred (− 49%) and DeepPocket_SEG_ (− 31%) (Fig. 9D–F), thus limiting the recall of these methods. Re-scoring and re-ranking of PUResNet (0.0%), PocketFinder^+^ (1.1%), Ligsite^+^ (0.6%), and Surfnet^+^ (1.6%) predictions did not have a significant effect (*p* > 0.05). For more details, see Supplementary Note 3, and Supplementary Figures 4, 5.

Supplementary Note 4 and Supplementary Figure 6 illustrate the powerful effect that removing redundancy, and a more robust pocket scoring scheme have when ranking predictions across multiple proteins, resulting in increases of up to + 600 TP by 100 FP. Moreover, whilst re-scoring fpocket predictions also results in an increase in # TP, DeepPocket_RESC_, DeepPocket_SEG-NR_ and fpocket_PRANK_, all starting from fpocket candidates, achieve no more than ≈ 600 TP, indicating that the maximum possible increase in recall achieved by pocket re-scoring is limited by the starting predictions, which are the same for these three methods. Finally, despite benefitting from a simple scoring of their pockets by taking the number of amino acids, PocketFinder^+^, Ligsite^+^ and Surfnet^+^ present the lowest # TP_100FP_. This is potentially because their purpose is the detection of cavities and not the prediction of ligand sites. Despite being similar tasks, they are not the same. This is reflected in the number of cavities they report and their size, which explain the low number of # TP_100FP_. Moreover, these methods are not machine learning-based and were not trained to learn from and recapitulate observed ligand binding sites.

Redundancy removal for VN-EGNN, IF-SitePred and DeepPocket_SEG_ does not have a significant effect (*p* ≥ 0.05) on the precision of these methods. However, (re-)scoring predictions of PUResNet, fpocket, PocketFinder^+^, Ligsite^+^, and Surfnet^+^ increased precision on the top-1000 predictions by + 11.7%, + 34.4%, 23.3%, 16.5% and 29.1%, respectively, further proving the importance of the employed pocket scoring scheme (Supplementary Note 5 and Supplementary Figure 7). Supplementary Figure 8 and Supplementary Table 2 summarise the results of this analysis.

## Discussion

In this work we have carried out the most complete comparative analysis of ligand binding site prediction methods to date, spanning three decades of methods development. Firstly, predictions from the thirteen methods as well as observed sites from our reference dataset, LIGYSIS, were compared in terms of number of proteins methods predict on, number of predicted sites per protein, their size, distance and overlap between the sites. This analysis provides insight into how the different methods work and hints at potential limitations or room for improvement, e.g., the prediction of a fixed number of sites per protein, or considerable proximity and overlap between the predictions. Secondly, predictions from thirteen canonical ligand site prediction methods, as well as fifteen method variants first introduced in this work are objectively evaluated using the LIGYSIS set. This evaluation considers predictions at the residue level by F1 score, MCC, ROC/AUC and PR/AP, as well as at the pocket level by Recall considering top-*N*, *N*+2 and *all* predictions, Precision_1K_, # TP_100 FP_, RRO, RVO and redundancy. This is the first independent ligand site prediction benchmark for over 10 years, since Chen et al. [67] and the largest to date both in terms of reference dataset size (2775 chains), number of methods compared (13 original + 15 variants) and metrics employed (> 10).

We have shown how redundancy in prediction, i.e., predicting multiple times the same observed site, can underestimate the recall, and overestimate the precision of the methods, therefore providing a misleading assessment of the methods’ performance. Redundancy removal and subsequent pocket re-ranking can yield a significant increase in recall. A robust pocket scoring scheme can have a strong impact in performance, both in the recall and precision of the methods and emphasis should be put into this area. Even if a single site is predicted per protein, a pocket score can be highly useful when ranking pockets across different proteins, e.g., when having a list of potential drug targets and deciding which protein might be best to target therapeutically.

Recall (% of sites that are correctly predicted) is more informative than precision (% of predictions that are correct), particularly, recall considering top-*N*+2 ranked predictions. In most cases, not all the existing binding pockets are observed with a ligand bound. In other words, the reference data are incomplete, with 33–50% of existing sites yet to be observed with ligands bound in a structure, as conjectured by Krivák and Hoksza [40]. Considering only the top-*N* predicted pockets assumes that there are exactly *N* real pockets for a given protein, which might not be the case. A method could predict a *real* pocket that is yet to be observed and rank it before other predicted and observed pockets. By considering the top-*N*+2 pockets, we are controlling to some extent for this noise in the reference data and obtaining a more accurate representation of the performance of a method. In a context of discovery where the true ligand binding sites of the target are unknown, it is more useful to have multiple predictions that might or might not correspond to real sites (lower precision), rather than a single or few predictions that are very precise but are missing other likely sites (lower recall). Most methods do well in predicting the most obvious (orthosteric) site. This site, however, might not be available for therapeutic targeting and it is convenient to have other predicted sites that could modulate function acting as allosteric sites. Precision, though a metric that provides valuable insight, and covered in this work, must always be contextualised with recall. Our results show that the most precise methods do not correspond to higher recalling methods. A method predicting the most obvious site, that could be identified by eye, might be 90% precise, but present a lower recall, e.g., 30%. This said, methods predicting fewer pockets with higher precision might prove more advantageous when users aim to study a particular region of interest in a protein, a few high-priority sites are needed for experimental validation, or false predictions are costly in a downstream analysis.

Some methods define “success rate” as the precision of the top-1 or top-3 scoring predictions, which is not a very representative performance assessment metric. For this reason, we encourage method developers not only to share the code of their approach, but also of the benchmarking analysis. Furthermore, the definition of *success rate* must be standardised as recall, as some methods use recall, whereas others use precision, both under the same term of success rate. This can be confusing when comparing the results from different analyses. Moreover, due to the inherent noise in the reference data, i.e., not all existing pockets are observed, recall considering top-*N*+2 is more informative than taking top-1, top-3 or top-*N* predictions. In any case, success rate must be clearly defined, so readers can fully understand the implications of the metric employed on a benchmark.

It is clear from our results that a DCC threshold of 4 Å is too conservative, and a more flexible DCC threshold of 10–12 Å should be used for comparable performance with DCA = 4 Å. According to our results and our reference set, predictions with DCC 4–12 Å overlap or are adjacent to observed sites and should be considered as correct predictions. The reason for this is the inherent noise in the ground truth, observed data or reference dataset, i.e., a ligand binding to a cavity might not be representative of all ligands that could bind to it. For most proteins, not all existing ligand sites are characterised, and as different ligands can bind to the same region, it is unrealistic to use such a small DCC threshold. Our results show several examples of predictions of an observed cavity where DCC > 4 Å.

fpocket_PRANK_ (60%) and DeepPocket_RESC_ (58%) present the highest top-*N*+2 recall of the methods reviewed in this work. P2Rank_CONS_ and P2Rank follow closely with 54% and 52% recall, then GrASP (50%), DeepPocket_SEG-NR_, Ligsite^+^_AA_ and PocketFinder^+^_AA_ with 49%, Surfnet^+^_AA_ (47%), VN-EGNN_NR_ (46%), PUResNet_PRANK_ (41%) and IF-SitePred_RESC-NR_ (39%). fpocket is the method that predicts the most pockets per protein, reaching a maximum recall between 80 and 90% (considering all pockets regardless of the rank). P2Rank_CONS_ comes second with a maximum recall of 50–60%. The rest of the methods range 40–55%. This indicates that whilst there are still some pockets un-predicted by fpocket (10–20%), the maximum recall of this method is 20–30% higher than any other method. However, considering top-*N*+2 pockets, fpocket only recalls 47% of the observed pockets. fpocket_PRANK_ and DeepPocket_RESC_ gain > 10% in recall by simply re-scoring those predictions. This difference also applies to # TP_100FP_, where PRANK re-scoring results in + 432 TP, and Precision_1K_, with an increase of + 34.4%. This highlights the paramount importance of a robust scoring scheme, which captures well the nature of binding sites and places those with a higher probability of being real binding sites at the top of the ranking. Newer methods like VN-EGNN, IF-SitePred, GrASP and PUResNet are the most precise methods, however because of redundancy in predictions (VN-EGNN, IF-SitePred), or low number of predicted pockets per protein (VN-EGNN, GrASP and PUResNet) are limited in their recall. Their high precision indicates that their models learn and capture well the nature of ligand binding sites and so they represent a great venue to pursue in the field of ligand binding site prediction. Whilst removing redundancy post-prediction has a significant improvement in performance (VN-EGNN_NR_ and IF-SitePred_NR_), approaching this issue before prediction would be more beneficial. For VN-EGNN, which predicts a maximum of 8 sites, ensuring these 8 (or more) predictions are non-redundant is more desirable than removing redundant predictions ending up with 1/8 predictions. The same applies to IF-SitePred, where non-overlapping starting predictions are more convenient than dealing with redundancy post-prediction.

The usefulness of residue-level metrics as F1 score or MCC is limited, as methods that precisely and correctly predict the clearest sites, such as PUResNet (high precision, low recall) perform better on these metrics, whilst methods that predict more pockets (lower precision, higher recall) such as fpocket and re-scored versions, will obtain worse results. Pocket-level metrics, particularly recall, are more representative of the ability to predict ligand binding sites.

Whilst datasets like PDBbind, binding MOAD or the brand new PLINDER [89] (will) prove extremely useful to train, validate and test deep learning models tackling problems such as rigid body docking [90], flexible pocket docking [91], or pocket-conditioned ligand generation [92], they might not be ideal as a test set for ligand binding site prediction. LIGYSIS analyses all unique, biologically relevant protein–ligand interfaces, including ions, across the biological assembly from multiple experimentally determined structures of a given protein. It then clusters these ligands based on their interactions with the protein, resulting in the observed binding sites. Beyond considering biological assemblies and unique protein–ligand interfaces, the greatest innovation in LIGYSIS is leveraging the extensive structural data on the PDBe-KB to aggregate ligand-binding interactions across different structures of the same protein, thus better capturing the ligand-binding capabilities than just taking a single structure of a protein–ligand complex. In doing so, LIGYSIS represents the most complete and non-redundant protein–ligand complex dataset to date. The benchmark is performed on LIGYSIS, which includes ion binding sites. When these are removed, all methods, except fpocket, experience an increase in (top-*N*+2) recall of 5–10% but the overall ranking of methods does not change (see Supplementary Figure 9). Due to its integrative approach, features, diversity and size (covering > 30% of PDB and > 20% of BioLiP) LIGYSIS is the most inclusive and representative dataset of protein–ligand interactions.

Aggregating protein–ligand interactions across structures of the same protein is likely to be beneficial not only for testing, but also when training these methods. Most current methods train on datasets where a protein is represented by a single structure interacting with a single ligand. For example, in 100% of sc-PDB and 50% of entries for binding MOAD training sets. Methods consider as ligand binding, and therefore TP, those residues within a certain distance of the ligand and TN all other residues. In doing so, residues of the same protein that bind ligands on other structures, but not the one present on these sets, will be incorrectly labelled as “non-ligand-binding” (FN). This mislabelling of residues could lead to a lower prediction performance. This issue is to a certain extent approached by P2Rank and GrASP, which enriched their training datasets by including ligands from other chains, or homologous structures. This noise in the training dataset might be more prevalent for DeepPocket, PUResNet and VN-EGNN, which seem to rely fully on 1:1 interactions. The usage of LIGYSIS, or any other data set that aggregates ligand interactions across structures, might alleviate this issue and hints at potential room for improvement in the field of ligand binding site prediction.

## Conclusions

The conclusions resulting from our analysis are as follows:

- Ligand binding site prediction methods differ significantly in the number of predicted sites, their size, proximity and overlap, which offers insight into how the methods work.
- Redundancy in ligand binding site prediction leads to an underestimate of recall and an overestimate of precision. The removal of such redundancy and subsequent re-ranking of the remaining pockets results in a drastic increase in recall.
- A robust pocket scoring scheme is crucial for the correct ranking and prioritisation of predicted sites in downstream analysis, e.g., docking, simulation. Additionally, it has a significant positive effect on both precision and recall.
- Recall is a more informative measure of the performance of a ligand site prediction tool, rather than precision and so it must be reported. Precision, though a useful metric, should always be contextualised with recall.
- All authors of ligand site prediction tools should use top-*N*+2 recall as “success rate” for consistency. Benchmarking code should also be shared by the authors for the sake of reproducibility.
- Pocket-level metrics (recall, precision) are a more adequate representation of the capabilities of ligand site prediction methods than residue-level metrics (F1, MCC).
- A DCC threshold of 4 Å is too conservative, and to obtain comparable results between DCA and DCC recall, a threshold of DCC of 10–12 Å should be employed.
- Re-scoring of fpocket predictions, as fpocket_PRANK_ or DeepPocket_RESC_ present the highest (top-*N*+2) recall (60%) among the methods reviewed in this analysis.
- Methods that systematically predict a low number of pockets, e.g., VN-EGNN, GrASP or PUResNet, are very precise (> 90%), however their recall is low, and might not be as useful in a discovery context.
- IF-SitePred benefits significantly from pocket re-scoring, and suggests that protein embeddings, which aren’t directly dependent of structure, represent great promise in the field of ligand site prediction.
- The use of duplicated protein–ligand interfaces in asymmetric units results in an overestimate of both precision and recall when benchmarking ligand site predictors. Only unique protein–ligand interfaces in biological units should be considered for a more accurate benchmark of the performance of these methods.
- LIGYSIS aggregates non-redundant biologically relevant protein–ligand interactions across multiple structures for a protein and sets a new test set standard for the benchmark of ligand binding site prediction tools.
- This work objectively evaluates the performance of 13 canonical ligand binding site prediction methods and 15 non-redundant and scoring variants using 10 different metrics. This analysis represents the largest benchmark of ligand binding site prediction to date.

## Methods

### Derivation of the LIGYSIS dataset

There are 20,423 human reviewed proteins in UniProt [93]. Only 7640 (37.4%) of these proteins present experimentally determined three-dimensional structures deposited in the Protein Data Bank (PDB) [94–96]. 5455 (71.4%) proteins present at least one ligand-binding structure. Only biologically relevant ligands, as defined by BioLiP [66] were considered, yielding a set of 3513 proteins including 4037 structural segments, as defined on the PDBe-KB. These segments represent a UniProt sequence region with one or more structurally overlapping chains [97]. A protein might be represented by multiple segments which usually correspond to different domains. Protein chains mapping to a given UniProt ID were obtained from the PDBe aggregated API endpoint: https://www.ebi.ac.uk/pdbe/graph-api/uniprot/superposition/ [98]. Transformation matrices to superpose protein chains for a structural segment were downloaded from the PDBe FTP site at http://ftp.ebi.ac.uk/pub/databases/pdbe-kb/superposition [99] and used to structurally align a total of 64,498 protein chains across 33,715 structures for the 4037 segments (see Supplementary Figures 12 and 13 for superposition examples and variation across chains for ligand binding residues).

**Fig. 10:**
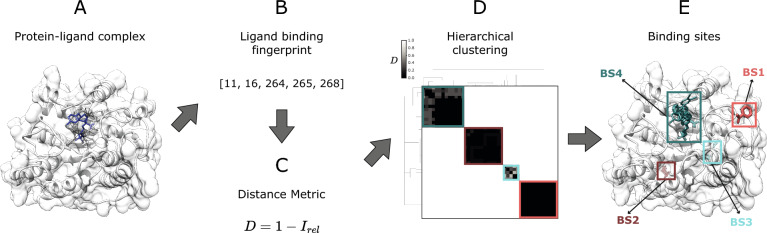
LIGYSIS ligand binding site definition algorithm. For a given protein–ligand interaction complex (**A**), a ligand binding fingerprint (**B**) is obtained as the set of unique UniProt sequence residue numbers interacting with the ligand as defined by pdbe-arpeggio; **C** fingerprints from different ligands binding to the same protein can be compared and a distance calculated; **D** this distance can be used to perform hierarchical clustering, which groups the different ligands in distinct clusters binding to the same region of the protein or binding site (**E**)

Preferred biological assemblies, as defined by PISA [68], were downloaded from PDBe via ProIntVar [100]. Protein–ligand contacts were determined with pdbe-arpeggio [101]. Figure 10 illustrates the ligand site definition approach used to obtain our reference dataset: LIGYSIS. For a pair of ligands, *L*_*A*_, *L*_*B*_, fingerprints *A*, *B* are defined as sets containing the UniProt residue numbers of the amino acids interacting with each ligand. PDB residues were cross-referenced to UniProt using the SIFTS mapping present in the mmCIF files, located under the _atom_site.pdbx_sifts_xref_db fields [102, 103]. Relative intersection, *I*_*rel*_, (Eq. 1) is a similarity metric that quantifies how similar these fingerprints are [5]. Subtracting *I*_*rel*_ from 1 gives a distance, *D* (Eq. 2), which takes the value of 0 when *A* and *B* share all the binding residues and 1 when they share none. For a given protein segment, interacting with *M* biologically meaningful ligands across *N* chains, ligand fingerprints are clustered using average linkage with SciPy [104] and ligand sites obtained by cutting the tree at *D* = 0.5. This resulted in 8244 ligand binding sites, i.e., sets of UniProt residues. This method for the definition of ligand binding sites is therefore independent of structure superposition and superposition is merely used for visualisation purposes.

1 $$I_{rel} = \frac{A \cap B}{{A \cap B_{max} }}$$

2 $$D = 1 - I_{rel}$$

### Comparison of datasets

Training and test datasets were downloaded for all machine learning based methods reviewed in this work. Datasets were compared to our reference set, LIGYSIS, in terms of number of sites per protein, ligand-interacting chains, chain lengths, size of the sites (number of amino acids), ligand composition, size and diversity. Ligand diversity was quantified by Shannon’s Entropy [105] (Eq. 3) where *p*_*i*_ represents the proportion of each ligand *i* of the *R* ligands observed in the dataset. Ligand data was extracted from the Chemical Component Dictionary (CCD) [106]. An overlap (%) was calculated for each dataset as the proportion of LIGYSIS binding sites that were covered by at least one ligand in a dataset. A simplistic approach was adopted by calculating the intersection of ligand IDs between LIGYSIS and each dataset. Ligand IDs were defined as a string of PDB ID + “_” + ligand ID, e.g., “6GXT_GTP” corresponds to the guanosine-5′-triphosphate of the PDB entry with ID: 6GX7 [107]. LIGYSIS considers all BioLiP relevant ligands across structures for a protein and dismisses ligands that are a byproduct of crystallisation. Most predictors also tend to discard such ligands for training purposes. For this reason, despite its simplicity, the approach here employed captures well the overlap between training, test sets and LIGYSIS.

3 $$H^{\prime} = - \mathop \sum \limits_{i = 1}^{R} p_{i} \ln \;\left( {p_{i} } \right)$$

#### Training datasets

VN-EGNN trains on a subset [44] of the sc-PDB (v2017) [108–112] (sc-PDB_SUB_). sc-PDB is a comprehensive database of pharmacological ligand–protein complexes. The database is comprised by proteins in complex with buried, biologically relevant synthetic or natural ligands deposited in the PDB. sc-PDB contains unique non-repeating protein–ligand pairs, which means that only one ligand is considered per PDB structure entry. Smith et al*.* [54] enriched this dataset with 9000 extra ligands resulting in a version of sc-PDB which we call sc-PDB_RICH_, which GrASP trained on. Unfortunately, this dataset is not publicly accessible and therefore not considered in our analysis. DeepPocket used the full sc-PDB set to train on, sc-PDB_FULL_. IF-SitePred uses a sequence identity-filtered version of the non-redundant subset of the binding mother of all databases (MOAD) [113–116], which considers only protein family leaders. The binding MOAD, here referred to as bMOAD_SUB_, is a large collection of crystal structures with clearly identified biologically relevant ligands with binding data extracted from the literature. Finally, PRANK and P2Rank used the CHEN11 dataset to train, which aimed to cover all SCOP [117–119] families of ligand binding proteins in a non-redundant manner [67]. P2Rank utilised the JOINED dataset for validation. CHEN11 not only considers the ligands in each structure but is enriched with ligands binding to homologous structures. JOINED is a combined dataset formed by other smaller datasets: ASTEX [120], UB48 [30], DT198 [121] and MP210 [34], which represent diverse collections of protein–ligand complexes, including bound/unbound states, drug-target complexes and other ligand site predictor benchmark sets.

#### Test datasets

The majority of ligand binding site predictors published since 2018 have been using two datasets that were first presented by Krivák et al*.* [40]: COACH420 and HOLO4K, or subsets of them. COACH420 is comprised by a set of 420 single-chain structures binding a mix of drug-like molecules and naturally occurring ligands which is disjunct with the CHEN11 and JOINED datasets. COACH420 is a modified version of the original COACH test set [26, 27]. HOLO4K is a larger set, *N* ≈ 4000, based on the list by Schmidtke et al*.* [122], which includes a mix of single- and multi-chain complexes, also disjunct with P2Rank training (CHEN11) and validation (JOINED) datasets. PRANK employed the small datasets comprising the JOINED set for testing. VN-EGNN, DeepPocket and GrASP use the Mlig and Mlig+ subsets of the COACH and HOLO4K datasets, which comprise strictly biologically relevant ligands as defined by the binding MOAD. IF-SitePred is tested on the HOLO4K-AlphaFold2 Paired (HAP) and HAP-small sets. HAP is a subset of the HOLO4K dataset which presents high quality models in the AlphaFold database [123]. HAP-small is a smaller subset of HAP that only contains proteins with sequence identity lower than 25% to proteins in the P2Rank training set. VN-EGNN uses the refined version of PDBbind (v2020), referred here as PDBbind_REF_ [124–129], as a third test set. Like binding MOAD, the PDBbind database provides a comprehensive collection of experimentally measured binding affinity data for macromolecular complexes. Specifically, the refined set includes those protein–ligand complexes for which binding data was obtained with the literature and met certain experimental quality thresholds. Lastly, SC6K is a dataset presented by Aggarwal et al*.* [48] containing 6000 protein–ligand pairs from PDB entries submitted from 01/01/2018 to 28/02/2020.

### Protein chain alignment

For each protein chain, atomic coordinates are translated to be centred at the origin, *O* = (0, 0, 0), and rotated using a rotation matrix, *R*. The two principal components of the coordinate space *pc*_*1*_ and *pc*_*2*_ are obtained using principal component analysis (PCA) [130]. A third component, *pc*_*⊥*_, (Eq. 4) is obtained with the cross-product of the other two, to ensure orthogonality. A rotation matrix *P* is constructed from these vectors (Eq. 5). By placing the main component *pc*_*1*_ on the second row of *P*, we ensure the *Y* axis will be the major axis, representing the *height* of the protein chain. The second largest axis will be the *X* axis, representing the *width* of the protein, and lastly the *depth* will be represented by the smaller magnitude of the *Z* axis. The final rotation matrix *R* (Eq. 6) is obtained by multiplying *P* by the negative identity matrix *NI* (Eq. 7). This was done to maintain the left-handedness of the protein chains whilst ensuring a consistent alignment on the major axes.

4 $$pc_{ \bot } = pc_{1} \times pc_{2}$$

5 $$P = \left[ {\begin{array}{*{20}c} {pc_{2} } \\ {pc_{1} } \\ {pc_{ \bot } } \\ \end{array} } \right]$$

6 $$R = P \cdot NI$$

7 $$NI = - 1 \cdot I_{3} = - 1 \cdot \left[ {\begin{array}{*{20}c} 1 & 0 & 0 \\ 0 & 1 & 0 \\ 0 & 0 & 1 \\ \end{array} } \right] = \left[ {\begin{array}{*{20}c} { - 1} & 0 & 0 \\ 0 & { - 1} & 0 \\ 0 & 0 & { - 1} \\ \end{array} } \right]$$

### Protein chain characterisation

For a protein chain with *N* amino acid residues, the centre of mass, *CM*, is calculated by averaging the coordinates, *r*_*i*_, of all atoms (Eq. 8), and from it, the radius of gyration, *R*_*g*_, is derived (Eq. 9) [131]. As the protein chains are already aligned on the axis and centred on *O* = (0, 0, 0) the dimensions of the protein chain can be obtained as the magnitude of the PCA components or *eigenvectors*, i.e., the *eigenvalues*. The dimensions represent width, height, and depth for the *X*, *Y* and *Z* axes, respectively.

8 $$CM = \frac{1}{n} \mathop \sum \limits_{i = 1}^{n} r_{i} \to CM = O = \left( {0, 0, 0} \right)$$

9 $$R_{g} = \sqrt {\frac{1}{n}\mathop \sum \limits_{i = 1}^{n} \left( {r_{i} - CM} \right)^{2} } \to R_{g} = \sqrt {\frac{1}{n}\mathop \sum \limits_{i = 1}^{n} \left( {r_{i} - O} \right)^{2} } = \sqrt {\frac{1}{n}\mathop \sum \limits_{i = 1}^{n} r_{i}^{2} }$$

**Fig. 11:**
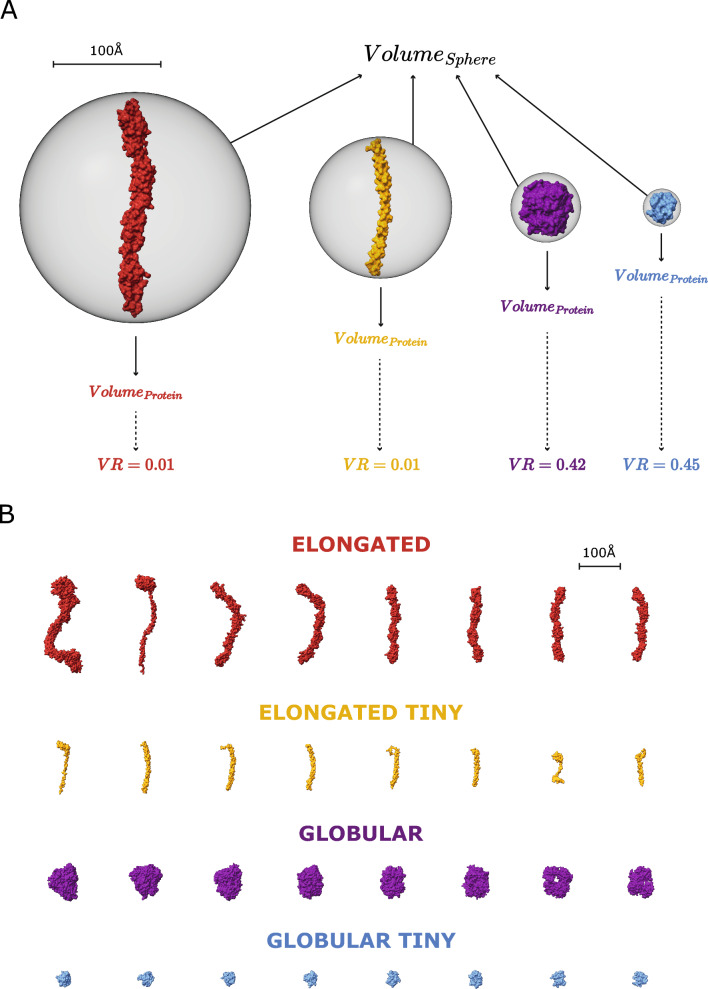
Protein chain shape and size classification approach. **A** The volume of the sphere enclosing the protein chain as well as the protein chain volumes are calculated, and their ratio obtained (VR). Globular proteins present more spherical shapes and therefore occupy a higher portion of the sphere volume, resulting in higher volume ratios. Non-globular, elongated or fibrous proteins on the other hand do not and present lower VRs. After extensive visual examination, a threshold was established at VR = 0.08, and so proteins classified in these two groups. Proteins were classified as “tiny” if their chain was ≤ 100 amino acids; **B** eight examples of each protein chain group to illustrate the outcome of the approach

Protein chain volumes were calculated using ProteinVolume [132]. A sphere enclosing the protein and centred on the protein centre of mass was obtained. The radius of this sphere is the maximum Euclidean distance between the protein atoms and the CM (Eq. 10). The volume of the sphere is calculated using Eq. 11. Proteins were classified into four different groups based on their shape and size. Protein chains with ≤ 100 amino acids were classified as “tiny”. Regarding the shape, protein chains were classified into “elongated” if their protein to sphere volume ratio ≤ 0.08 (VR) (Eq. 12), i.e., the protein volume contains no more than 8% of the sphere volume. This threshold was derived empirically by the visual inspection of all 3448 protein chains on the LIGYSIS set. Otherwise, proteins were considered globular (Fig. 11). In this manner, protein chains were classified into *globular* (*N* = 2104; 61%), *elongated* (*N* = 670; 19%), *elongated tiny* (*N* = 341; 10%) and *globular tiny* (*N* = 333; 10%).

10 $$R =\max\|r_i - CM\|$$

11 $$Volume_{Sphere} = \frac{4}{3}\pi R^{3}$$

12 $$VR = Volume_{Protein} /Volume_{Sphere}$$

### Ligand binding site prediction

For each segment in the LIGYSIS dataset, the representative chain as defined in the PDBe-KB was selected. Structures were cleaned using the *clean_pdb.py* script [133]. Eleven different ligand binding site prediction tools, with their default settings, were employed to predict on the 3448 representative chains: VN-EGNN [56], IF-SitePred [55], GrASP [54], PUResNet [44], DeepPocket [48], P2Rank [37, 40], PRANK [37], fpocket [7, 134], PocketFinder^+^ [15], Ligsite^+^ [8], and Surfnet^+^ [9]. Conservation scores were obtained from PrankWeb https://prankweb.cz/ and used for further prediction. This variant of P2Rank employing amino acid conservation is referred to as P2Rank_CONS_ [63, 135]. When running DeepPocket, the -*r* threshold was removed and so all fpocket candidates were passed to the CNN-based segmentation module for pocket shape estimation. fpocket predictions re-scored by DeepPocket are referred to as DeepPocket_RESC_, whereas pockets extracted by the segmentation module of DeepPocket are referred as to DeepPocket_SEG_. fpocket predictions were also re-scored with PRANK [37] (fpocket_PRANK_), as introduced in previous studies [37, 38, 40, 87]. Re-implementations by Capra et al*.* [33] were used for PocketFinder, Ligsite and Surfnet, indicated by the “^+^” superscript. VN-EGNN, IF-SitePred, PocketFinder^+^, Ligsite^+^ and Surfnet^+^ do not provide a list of residues for each pocket, but a list of centroids and their scores for the first two, and a list of grid points for each predicted pocket for the last three. For VN-EGNN, residues within 6 Å of the virtual nodes were considered pocket residues. For 429 predicted pockets (≈ 3%) no residues were found within this threshold. For IF-SitePred, residues within 6 Å of the clustered cloud points that resulted on a predicted pocket centroid were considered as pocket residues. Pocket residues were obtained in a similar manner for PocketFinder^+^, Ligsite^+^ and Surfnet^+^, by taking those residues within 6 Å of pocket grid points. In total, thirteen methods are considered on this analysis: VN-EGNN, IF-SitePred, GrASP, PUResNet, DeepPocket_RESC_, DeepPocket_SEG_, P2Rank_CONS_, P2Rank, fpocket_PRANK_, fpocket, PocketFinder^+^, Ligsite^+^ and Surfnet^+^.

Seven of the considered methods provide residue *ligandability* scores. P2Rank and P2Rank_CONS_ report calibrated probabilities of residues being ligand-binding. Similarly, GrASP predicts the likelihood for any given heavy atom to be part of a binding site. A residue-level score was obtained for GrASP predictions by taking the maximum score of the residue atoms. For IF-SitePred, a residue ligandability score *LS* can be obtained by averaging the 40 independently predicted probabilities of a residue being ligand-binding (Eq. 13). Though calculated in a different way, these three scores range 0–1 and represent the likelihood of a residue binding a ligand and can therefore be compared. PocketFinder^+^, Ligsite^+^, and Surfnet^+^ also provide residue scores which maximum value can be > 1.

13 $$LS = \frac{1}{40} \mathop \sum \limits_{i = 1}^{40} p_{i}$$

VN-EGNN, PUResNet, DeepPocket_RESC_, DeepPocket_SEG_, fpocket_PRANK_ and fpocket do not report residue-level scores. However, binary labels represent whether a residue is part of a pocket (1) or not (0), in the same manner as all other methods.

Throughout this work the terms “site” and “pocket” are used indistinctly. Across all figures, tables and legends, methods are sorted in chronological order.

### Binding site characterisation

**Fig. 12:**
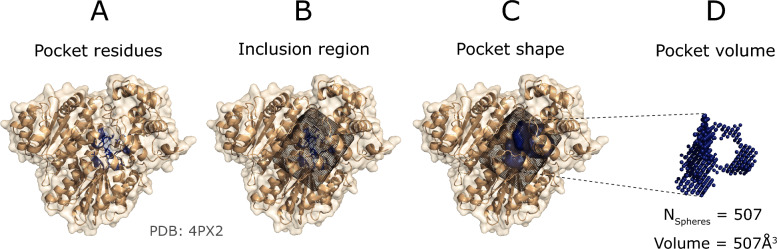
Pocket volume calculation algorithm. **A** PUResNet predicted pocket for PDB: 4PX2 [141]. Pocket residues are coloured in blue and have their side chains displayed; **B** an inclusion region is determined using the coordinates of the pocket residue atoms; **C** POVME 2.0 calculates the shape of the pocket defined by the residues and contained within the inclusion region; **D** The pocket shape is defined by a series of unit-volume (1 Å^3^) spheres. The volume of the pocket is calculated as the addition of the sphere volumes or the number of spheres within the pocket. Structure visualisation with PyMOL v2.5.2 [61]

Radius of gyration, *R*_*g*_, was calculated for pockets as it was done for whole protein chains (Eq. 9). Distance between pockets was calculated as the Euclidean distance between their centroids and overlap between pocket residues with the Jaccard Index (JI), or intersection over union (IOU) (Eq. 14) [136, 137]. POVME 2.0 was employed for pocket volume calculation [138–140]. A single inclusion region was used for each pocket. This region is defined by the smallest rectangular prism containing all pocket atoms. The prism is centred on the pocket centroid and its dimensions are determined by the distance between the two farthest atomic coordinates on each axis. No exclusion regions were used. Points outside the convex hull were deleted. A contiguous-points region was defined as a 5 Å-radius sphere on the pocket centroid (Fig. 12).

14 $$JI\left( {A, B} \right) = \frac{{\left| {A \cap B} \right|}}{{\left| {A \cup B} \right|}}$$

### Prediction evaluation

LIGYSIS binding sites consist of sets of UniProt residue numbers to which ligands bind across the multiple structures of a protein. The thirteen ligand binding site predictors benchmarked in this work predict only on the representative chains for each protein. These representative structures are defined in the PDBe-KB based on three criteria: data quality, sequence coverage and resolution [98]. Despite this, representative chains might still be missing some residues present in other structures. To compare LIGYSIS binding sites to predicted sites on the representative chains, UniProt sequence mappings are needed for each residue in the LIGYSIS-defined sites. For this reason, LIGYSIS entries with ligand-binding residues missing UniProt residue mappings on the protein’s representative chain were removed from our set, resulting in a set of 3048 human proteins, including 3448 segments. After predicting on these 3448 LIGYSIS chains, only chains where all residues across predicted sites presented UniProt residue mappings were kept. This resulted in a final set of 2775 protein chains which was employed to assess the performance of the methods. These filters are necessary to compare LIGYSIS-defined sites to predicted sites as UniProt residue overlap between defined and predicted sites is one of the criteria to evaluate the methods.

The performance of ligand binding site prediction methods can be evaluated at two different levels: *residue* level, and *pocket* level. Prediction at the *residue* level involves the discrimination of those residues that are likely to interact with a ligand, whereas the aim of *pocket*-level prediction is to define distinct regions on a protein, i.e., pockets where *a* ligand is likely to bind. This region can either be defined by a (pocket) centroid, a group of cloud/grid points, a set of pocket residues, or a combination of these. Some methods are *residue*-centric, and first predict at the residue-level, use a threshold to select high-probability ligand-binding residues, and then cluster them into pockets. Residue-centric methods include IF-SitePred, or GrASP. Other methods (*pocket*-centric) directly predict the location or shape of the pocket, without the need of predicting at the residue level first. Some of these methods can use their pocket-level prediction to report residue *ligandability* scores, e.g., P2Rank_CONS_, P2Rank, PocketFinder^+^, Ligsite^+^ or Surfnet^+^, and others, such as VN-EGNN, PUResNet, DeepPocket, or fpocket do not report residue ligandability scores.

Prior to evaluation, non-redundant sets of predictions were generated for VN-EGNN, IF-SitePred and DeepPocket_SEG_, as these methods generate a considerable proportion of *redundant* predictions. A predicted pocket $i$ is considered redundant if there exists a pocket *j* ≠ *i* so that the distance between their centroids *D*_*i,j*_ ≤ 5 Å or their residue overlap *JI*_*i,j*_ > 0.75, i.e., they share at least ¾ of their residues. Refer to Supplementary Figure 14 for the closest predicted sites for each method.

PUResNet, PocketFinder^+^, Ligsite^+^ and Surfnet^+^ do not score, nor explicitly rank their pockets, and so they were taken in the order given by their pocket ID. This means that when sorting across the dataset, the order of all pockets with the same rank is arbitrary. To obtain a score for these pockets, multiple strategies were employed. Firstly, a pocket score was obtained as the number of pocket amino acids, resulting in variants PUResNet_AA_, PocketFinder^+^_AA_, Ligsite^+^_AA_ and Surfnet^+^_AA_. Secondly, PRANK pocket scoring was employed, resulting in variants PUResNet_PRANK_, PocketFinder^+^_PRANK_, Ligsite^+^_PRANK_ and Surfnet^+^_PRANK_. IF-SitePred uses a simple pocket scoring scheme, which assigns to each centroid the number of clustered cloud points it results from. In this work, newly defined IF-SitePred pocket scores were calculated as the sum of squares (SS) of the ligandability scores (*LS*_*i*_), calculated with (Eq. 13), of the *K* residues on a site (Eq. 15) resulting in IF-SitePred_RESC_. For PocketFinder^+^, Ligsite^+^, and Surfnet^+^ the same was done but instead of residue scores, grid point scores (*GS*_*i*_) were used (Eq. 16). This resulted in further variants PocketFinder^+^_SS_, Ligsite^+^_SS_, and Surfnet^+^_SS_. This is the same approach introduced by Krivák, et al. [37] and later adopted by Smith et al*.* [54].

15 $$SS_{IF - SitePred} = \mathop \sum \limits_{i = 1}^{K} LS_{i}^{2}$$

16 $$SS_{{PocketFinder^{ + } , Ligsite^{ + } , Surfnet^{ + } }} = \mathop \sum \limits_{i = 1}^{K} GS_{i}^{2}$$

### Residue level predictions

GrASP, P2Rank_CONS_, P2Rank, PocketFinder^+^, Ligsite^+^ and Surfnet^+^ all offer residue ligandability scores. Additionally, a ligandability score was derived for IF-SitePred using Eq. 13. Prediction at the residue level is a binary classification problem: binding (1) or non-binding (0). Given a ligandability threshold *t*_*LS*_, a residue with a ligandability score *LS*_*i*_ is classified as “positive” if *LS*_*i*_ > *t*_*LS*_. Conversely, the residue is classed as “negative” if *LS*_*i*_ ≤ *t*_*LS*_. Further stratification results from comparing the predictions to the LIGYSIS reference dataset.

- True positive (TP): residue classified as *positive* that binds a ligand according to the reference.
- False positive (FP): residue classified as *positive* that does not bind a ligand in the reference.
- True Negative (TN): residue classified as *negative* that does not bind a ligand.
- False Negative (FN): residue classified as *negative* but is known to bind a ligand according to the reference.

With these four classes, true positive rate (TPR) (Eq. 17), false positive rate (FPR) (Eq. 18), precision (Eq. 19) and recall (Eq. 20) can be calculated and receiver operating characteristic (ROC), precision-recall (PR) curves plotted. ROC and PR curves were obtained for each of the LIGYSIS protein chains. Using these curves, mean ROC and PR curves, representative of the variation across proteins for these metrics were obtained by taking the mean TPR and FPR (ROC) and mean precision and recall (PR) at each score interval. Mean Area under the curve (AUC) and mean Average Precision (AP) were calculated by averaging the areas and precisions across curves. Baselines for these are 0.5 and the proportion of true binding residues (0.1), respectively. ROC and AUC can’t be calculated for VN-EGNN, PUResNet, DeepPocket, fpocket_PRANK_ and fpocket as these methods do not provide residue ligandability scores.

17 $$TPR = \frac{TP}{{TP + FN}}$$

18 $$FPR = \frac{FP}{{FP + TN}}$$

19 $$precision = \frac{TP}{{TP + FP}}$$

20 $$recall = \frac{TP}{{TP + FN}}$$

Pocket binary labels (0: no pocket residue; 1: pocket residue) can also determine TP, FP, TN and FN for each residue in a protein chain *P*_*i*_. VN-EGNN, IF-SitePred, PocketFinder^+^, Ligsite^+^ and Surfnet^+^ do not report pocket residues. For these methods, residues within 6 Å of the pocket centroid, cloud points and grid points (3×), respectively, were labelled as pocket residues (1). All other residues were labelled as non-binding (0). Across all residues in *P*_*i*_, an F1 score *F1*_*i*_ is computed, which combines precision and recall into a unified metric, capturing the accuracy and completeness of predictions at the residue level (Eq. 21). The Matthews Correlation Coefficient (MCC) [142] (Eq. 22) was also calculated. The mean F1 score and MCC across LIGYSIS proteins is reported for each method.

21 $$F1_{i} = \frac{{2 \times Precision_{i} \times Recall_{i} }}{{Precision_{i} + Recall_{i} }}$$

22 $$MCC = \frac{TP \times TN - FP \times FN}{{\sqrt {\left( {TP + FP} \right)\left( {TP + FN} \right)\left( {TN + FP} \right)\left( {TN + FN} \right)} }}$$

### Pocket level predictions

Ligand binding site prediction at the pocket level is a multi-instance prediction problem. There are no *negatives* predicted, only *positives*. A positive is a predicted pocket, which will be true (TP) or false (FP) depending on whether it is observed in the reference data. False negatives are those pockets observed in the reference data that are not predicted. They are the pockets the method fails to predict, and therefore, are not scored. A true negative would be a “non-pocket” that is not predicted. This can’t be quantified easily and even if it was, it would not be scored by the method, as it is not predicted. For this reason, in this context, neither TPR, nor FPR can be calculated. Consequently, ROC/AUC can’t be utilised to assess ligand binding site prediction at the pocket level. False negatives are known, but not scored, and therefore PR/AUC is not an option either. What can be calculated is the recall given a certain criterion. In this case, because of the nature of the LIGYSIS dataset, where defined sites result from the clustering of multiple ligands, the distance between the predicted pocket centroid to the observed binding site (DCC) was chosen as a criterion.

For each observed binding site in our reference dataset, the “best” prediction for each method is chosen. This is defined as the prediction with the minimum Euclidean distance to the observed pocket centroid or DCC. Once the observed-predicted pairs were obtained, only those with DCC ≤ 12 Å were considered as correct predictions. A threshold of 12 Å was chosen as 4 Å is too strict a threshold when using DCC (Supplementary Note 6 and Supplementary Figures 15–19). A threshold of 4 Å works well for the distance to closest ligand atom (DCA) but does not for DCC. The top-*N* and *N*+2 ranking predictions were considered to calculate success rate, or recall (Eq. 23), and maximum recall was calculated by considering *all* predictions, regardless of their score or rank. *N* represents the number of observed sites for a given protein.

23 $$Success\;rate \left( {recall} \right) = \frac{{observed\;sites\;with\;predicted\;site\;DCC \le 12\,{\mathring{\text{A}}}}}{observed\;sites}$$

Additionally, instead of conventional ROC, ROC100 [143, 144] can be used to measure the predictive performance of the methods. To do this, for each method, *all* predictions across dataset proteins were ranked based on the pocket score and cumulative true positives were plotted against cumulative false positives until 100 false positives were reached. In a similar way, a precision curve can be calculated by taking the top-*N*, in this case *N* = 1000, predictions, which measures how precision changes as more predictions with lower scores are considered. This is indicative of how informative pocket scores are.

Precision and recall are key measures for evaluating the performance of ligand binding site prediction methods. However, these indicators are calculated and interpreted slightly differently depending on the context a prediction is analysed, i.e., pocket vs residue level, as well as the metric employed, e.g., F1 score, MCC, ROC or PR curves. At the residue level, the prediction is a binary classification task, where each residue is classified as binding (1) or non-binding (0). Here, precision reflects the proportion of residues predicted as binding that are true, i.e., observed in the reference data. Recall measures the proportion of true binding residues that are correctly identified. For the calculation of F1 and MCC, a residue is labelled “positive” or “negative” depending on whether it is part of a predicted pocket. However, for ROC and PR curves, the positive and negative labels are derived based on a ligandability threshold, *t*_*LS*_. Prediction at the pocket level represents a multi-instance prediction task. Precision indicates the proportion of predicted pockets that are observed in the reference data whilst recall represents the proportion of true binding pockets that are correctly predicted. It is important to keep this in mind to correctly interpret precision and recall across different contexts.

**Fig. 13:**
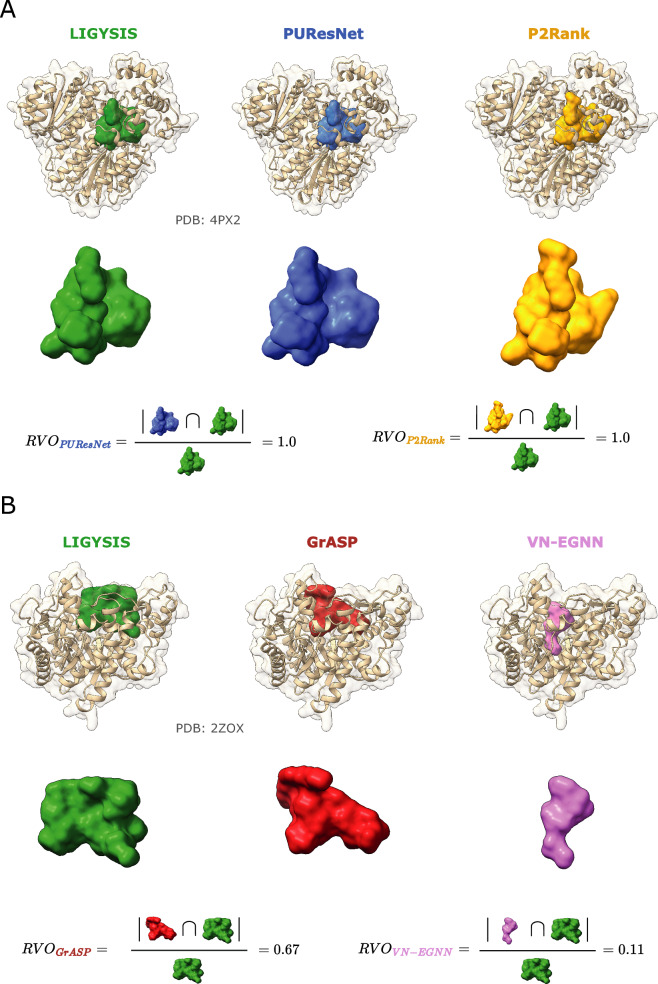
Relative volume overlap (RVO) calculation. **A** Example of two very accurate predictions by PUResNet and P2Rank on PDB: 4PX2 [141]. Pocket volumes are calculated with POVME 2.0 [139] and represented by coloured surfaces. These volumes result from the addition of unit-volume spheres on a grid. To obtain the RVO, the intersection of these spheres between predicted and observed site is divided by the number of observed pocket spheres. Both predictions cover the entirety of the observed pocket volume; **B** GrASP and VN-EGNN predictions of a site on PDB: 2ZOX [145]. The volumes of these predicted sites overlap less with the observed site: RVO = 0.67 for GrASP and RVO = 0.11 for VN-EGNN

To measure the similarity in shape and residue membership between the predicted and observed pockets, relative residue overlap (RRO) and relative volume overlap (RVO) were employed. For an observed-predicted pocket pair, RRO represents the proportion of observed ligand-binding residues (*R*_*o*_) that are covered by the predicted pocket residues (*R*_*p*_) (Eq. 24). The POVME output was utilised for the calculation of RVO (Fig. 13). POVME defines the volume of a pocket as a series of equidistantly spaced spheres of unit volume. As predictions by the different methods were on the same coordinate reference, these pocket volume spheres were already aligned, and the volume overlap was calculated simply as the proportion of spheres in the observed pocket (*V*_*o*_) that overlap with the predicted pocket spheres (*V*_*p*_) (Eq. 25).

24 $$RRO = \frac{{\left| {R_{p} \cap R_{o} } \right|}}{{R_{o} }}$$

25 $$RVO = \frac{{\left| {V_{p} \cap V_{o} } \right|}}{{V_{o} }}$$

### Statistics and reproducibility

VN-EGNN was installed and run locally from https://github.com/ml-jku/vnegnn. Likewise, for IF-SitePred: https://github.com/annacarbery/binding-sites. GrASP was obtained from https://github.com/tiwarylab/GrASP and predictions generated using their Google Colab Notebook. PUResNet predictions were obtained through the PUResNet v2.0 web server: https://nsclbio.jbnu.ac.kr/tools/jmol. DeepPocket was installed and executed locally: https://github.com/devalab/DeepPocket. P2Rank v2.4.2 was used to run all predictions as well as PRANK scoring: https://github.com/rdk/p2rank. fpocket v4.0 was installed via Conda: https://anaconda.org/conda-forge/fpocket. For PocketFinder, Ligsite and Surfnet, the ConCavity v0.1 “^+^” re-implementations were employed: https://compbio.cs.princeton.edu/concavity/.

Other recent methods including RefinePocket [53], EquiPocket [52], GLPocket [51], SiteRadar [50], NodeCoder [49], RecurPocket [47], PointSite [46], DeepSurf [45], Kalasanty [43], BiteNet [42] GRaSP [41], or DeepSite [39] were not included in this analysis due to technical reasons. Peer-reviewed open-source methods with publicly accessible code, clear installation instructions, well defined dependencies, accessible command line interfaces and trained machine learning models were prioritised in this work. This set of thirteen methods, counting _RESC_ and _SEG_ variants of DeepPocket is representative of the state-of-the-art within the field.

ChimeraX v1.7.1 [146] was used for structural visualisation in all figures unless otherwise stated, in which case PyMOL v2.5.2 was employed [61].

## Supplementary Information

Supplementary Material 1.

## Data Availability

The main results tables and files necessary to replicate the analysis described in this paper can be found here: 10.5281/zenodo.13121414 [147].

## Abbreviations

0XR: Ethyl caffeate
017: Darunavir
3D: Three-dimensional
A32: BMSC-0010
AA: Amino acid
ADE: Adenine
ADP: Adenosine-5′-diphosphate
AP: Average precision
ATP: Adenosine-5′-triphosphate
AUC: Area under the curve
BGC: Glucose
C5P: Cytidine-5'-monophosphate
CI: Confidence interval
CLA: Chlorophyll A
CLR: Cholesterol
CM: Centre of mass
CNN: Convolutional neural network
DBSCAN: Density-based spatial clustering of applications with noise
DCA: Distance to closest ligand atom
DCC: Distance centroid to centroid
DGD: Digalactosyl diacyl glycerol
DNN: Deep neural network
DRN: Deep residual network
EGNN: Equivariant graph neural network
FAD: Flavin-adenine dinucleotide
FMN: Flavin mononucleotide
FN: False negative
FP: False positive
FUC: Fucose
G2P: Phosphomethylphosphonic acid guanylate ester
GAI: Guanidine
GAL: Galactose
GAT: Graph attention network
GFB: GDP-L-fucose
GNN: Graph neural network
GSH: Glutathione
HAP: HOLO4K-AlphaFold2 paired
HEM: Haem
IOU: Intersection over union
IQR: Interquartile range
I_rel_: Relative intersection
JI: Jaccard Index
LightGBM: Light gradient boosting machine
LJ: Lennard–Jones
MAN: Mannose
MCC: Matthews correlation coefficient
MCD: Minimum centroid distance
MRO: Maximum residue overlap
NAD: Nicotinamide-adenine-dinucleotide
NAP: Nicotinamide-adenine-dinucleotide phosphate
NI: No-ions
N-mer: Protein peptide of N amino acids
NR: Non-redundant
PCA: Principal component analysis
PID: Peridinin
PLP: Vitamin B6 phosphate
PR: Precision recall curve
R_g_: Radius of gyration
ROC: Receiver operating characteristic
RRO: Relative residue overlap
RVO: Relative volume overlap
RSA: Relative solvent accessibility
SD: Standard deviation
SAH: *S*-Adenosyl-l-homocysteine
SAS: Solvent accessible surface
SFG: Sinefungin
SS: Sum of squares
TN: True negative
TP: True positive
VN: Virtual node
VR: Volume ratio
XYP: Xylose

## Code availability

Software developed to carry out this analysis is found in our GitHub repository: https://github.com/bartongroup/LBS-comparison [148].
