# Supplementary material for: Comparative evaluation of methods for the prediction of protein–ligand binding sites
Source: J Cheminform. 2024 Nov 11;16:126. doi: 10.1186/s13321-024-00923-z (PMC11552181; doi:10.1186/s13321-024-00923-z)
Supplement: Supplementary file 1 — Supplementary Material 1. [file 13321_2024_923_MOESM1_ESM.pdf]

Comparative evaluation of methods for the prediction of protein-ligand binding sites

Supplementary Information

Javier S. Utgés and Geoffrey J. Barton*

Division of Computational Biology, School of Life Sciences,

University of Dundee, Dow Street, Dundee, DD1 5EH, Scotland, UK

*Correspondence to: gjbarton@dundee.ac.uk

Method	Source	Review	Install	Docs	Model	Included
**VN-EGNN**	✓	✓	✓	✓	✓	✓
**IF-SitePred**	✓	✓	✓	✓	✓	✓
**GrASP**	✓	✓	✓	✓	✓	✓
RefinePocket	✓	✓	?	×	✓	×
EquiPocket	✓	×	?	×	✓	×
GLPocket	✓	✓	?	×	✓	×
SiteRadar	×	✓	×	×	×	×
NodeCoder	✓	×	?	✓	×	×
**DeepPocket**	✓	✓	✓	✓	✓	✓
RecurPocket	✓	×	?	×	✓	×
PointSite	✓	✓	×	✓	✓	×
DeepSurf	✓	✓	×	✓	✓	×
**PUResNet**	✓	✓	✓	✓	✓	✓
Kalasanty	✓	✓	×	✓	✓	×
BiteNet	×	✓	×	✓	×	×
GRaSP	✓	✓	✓	×	✓	×
**P2Rank**	✓	✓	✓	✓	✓	✓
**PRANK**	✓	✓	✓	✓	✓	✓
DeepSite	×	✓	×	×	×	×

Supplementary Table 1. Selection criteria for the nineteen machine learning-based methods considered in this study. These are the criteria employed to select machine learning-based

methods for this benchmark. Source: whether the method is open source and code is publicly accessible; Review: whether the method has been published after peer-review; Install: whether installation of the method was successful; Docs: whether the method is sufficiently documented to install it and run it on an example input; Model: whether the method provides pre-trained model weights; Included: whether the method was included in this analysis. Check marks(✓) indicate meeting the requirement and crosses (×) the opposite. Question marks (?) indicate uncertainty. Installation was not attempted for some methods as they already did not meet other requirements. Methods in bold font are the ones included in this work.

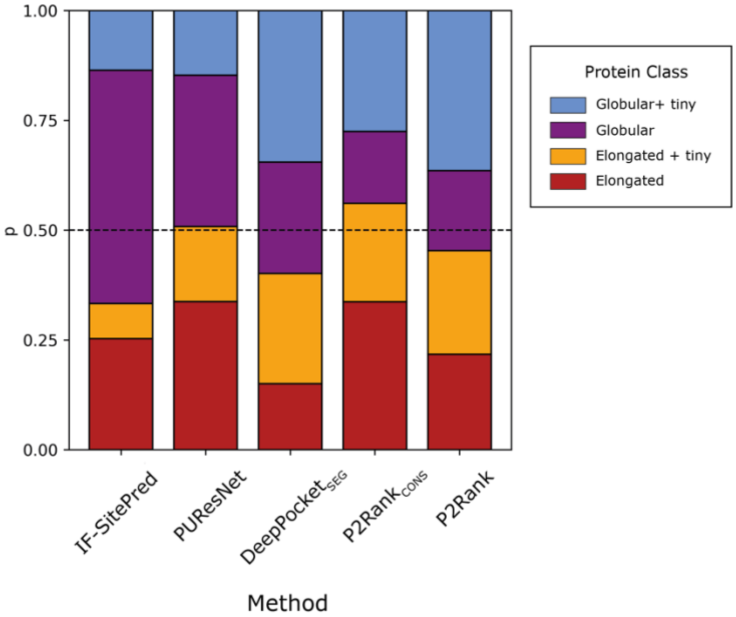

Supplementary Figure 1. Where methods do not predict any sites. IF-SitePred does not predict any ligand binding sites on 700 of the 2,775 protein chains in the LIGYSIS set (25%), PUResNet on 415 (15%), DeepPocket$_{SEG}$ (426; 15%), P2Rank$_{CONS}$ (196; 7%) and P2Rank (373; 13%). All methods struggle to predict on elongated proteins, regardless of their size, as well as on tiny globular proteins. Globular proteins comprise the most common group amongst the proteins with no predictions for IF-SitePred (53%).

Supplementary Note 1: Number of Pockets vs Protein Size

IF-SitePred [1], DeepPocket_{SEG} [2], P2Rank_{CONS} [3, 4], P2Rank [5] and fpocket [6, 7] predict more pockets as protein chain size, i.e., number of amino acids, increases, with fpocket being the clearest example. DeepPocket_{SEG} follows, as it takes as candidates fpocket predicted pockets. P2Rank_{CONS}, which is P2Rank passing evolutionary conservation scores as an extra feature predicts fewer pockets than P2Rank. Lastly, LIGYSIS does not follow this trend. It is true that in principle, the larger a protein is, the larger the solvent accessible surface area is, and more pockets are possible. However, in the observed data, the number of ligand sites per protein will depend mostly on the number of experimentally determined structures of a protein. Regardless of how big the protein is, if there are only a few structures, this will most likely lead to a small number of defined ligand binding sites.

VN-EGNN [8], GrASP [9], PUResNet [10, 11], PocketFinder^{+} [12], Ligsite^{+} [13] and Surfnet^{+} [14] do not predict more sites as protein chain size increases, and the number of predicted pockets stays constant regardless of the protein chain size. VN-EGNN places K = 8 virtual nodes, by default, which are passed to the equivariant graph neural network (E-GNN). Eventually these virtual nodes converge on the location of the predicted binding pocket centroids. As a result, a maximum of N = 8 binding pockets can be predicted by VN-EGNN with default parameters. This is not an argument on VN-EGNN command line interface but can be changed in the source code. Despite this, our results show a maximum of N = 7 pockets. This means that one of the 8 virtual nodes always converges in the same location of another pocket, resulting in a maximum of 7 and not 8 pockets. In the case of GrASP, the distribution of the number of predicted pockets per chain is narrow, with a maximum of 12. This might be due to the conservative per-residue ligandability scores of GrASP, or their clustering strategy. PUResNet predicts a single pocket in 90% of the cases and a maximum of 4. Ligsite^{+} and Surfnet^{+} (geometry-based) and PocketFinder^{+} (energy-based) are implementations of the original methods by Capra *et al.*, 2009 [15]. They all use a grid of points which are then scored and clustered to define the pockets. They systematically predict fewer pockets than the methods above-mentioned with a median of 2-3 pockets per protein.

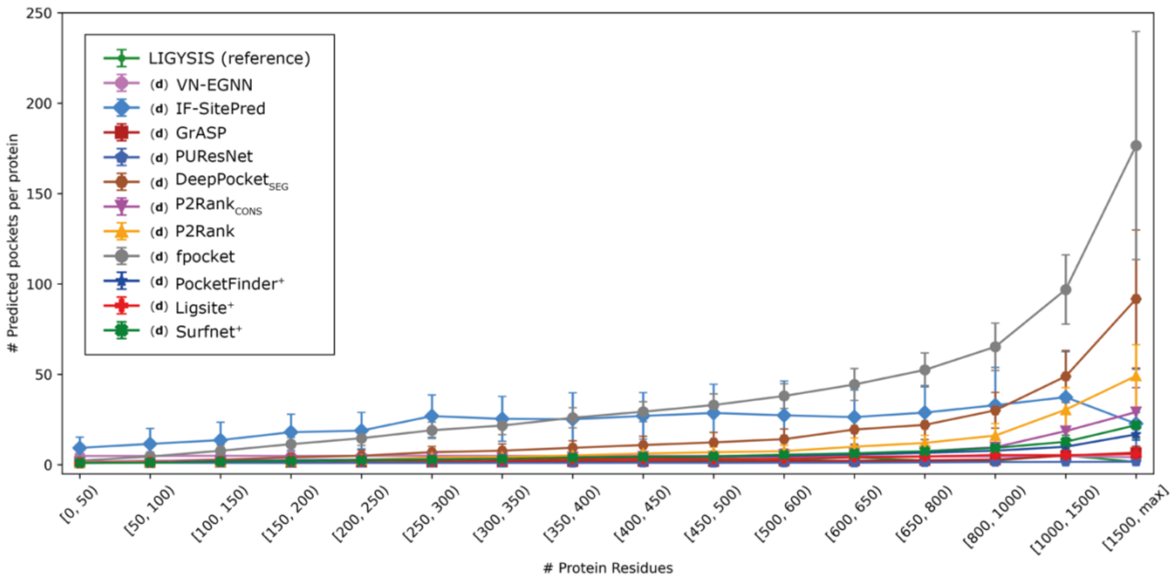

Supplementary Figure 2. Protein chain length vs number of pockets. Number of defined (LIGYSIS) and predicted sites against protein chain size, i.e., number of amino acid residues. Number of residues has been discretised into intervals of 50 until 650, and larger intervals until the maximum, ≈3,800. Error bars represent one standard deviation (SD). (**d**) preceding method names indicate that these are predictions by default methods and not variants.

Supplementary Note 2: Pocket Scoring, Ranking and Redundancy

Predictions by VN-EGNN, IF-SitePred, and $\text{DeepPocket}_{\text{SEG}}$ are highly redundant, i.e., >50% of their predicted pockets are within 5Å or present *JI* ≥ 0.75 with another predicted pocket. This is particularly problematic form VN-EGNN (67%) and IF-SitePred (50%), and less so for $\text{DeepPocket}_{\text{SEG}}$ (31%). Ligand binding site predictors are usually evaluated by taking the top-N or N+*2* predictions, where N is the number of known pockets for a protein. The redundant prediction of pockets will result in a sub-optimal ranking. Redundancy in prediction can often result in an overestimate of the precision and an underestimate of the recall.

For this reason, non-redundant "$_{\text{NR}}$" variants of these methods were generated. Moreover, methods like PUResNet, PocketFinder^{+}, Ligsite^{+} or Surfnet^{+} do not report pocket scores, and therefore their predictions are, to the best of our knowledge, not ranked in a systematic manner. To explore the effect this has on pocket ranking and performance "$_{\text{PRANK}}$", "$_{\text{AA}}$", "$_{\text{RESC}}$" and "$_{\text{SS}}$" re-scoring variants were generated.

Because of K = 8 virtual nodes are used in the default VN-EGNN implementation, a maximum of N = 8 predicted pockets are possible. However, only seven are observed in our dataset, as in all cases at least one virtual node gets clustered with another, resulting in 7 "unique" predictions. Supplementary Figure 3A illustrates the issue of redundancy in pocket predictions and how it affects the scoring and ranking of the pockets. A prediction of the same pocket is reported multiple times as distinct virtual nodes, or pocket centroids, which are very close to each other, and present very similar scores. This is why there is no apparent difference in the distribution of scores across the ranks for VN-EGNN, unlike all other methods. After removing redundancy, this is no longer the case (Supplementary Figure 3B).

IF-SitePred predictions are also highly redundant, however, these predictions, despite being close to each other, will present different scores (number of points), that is why higher ranks (1, 2, 3...) present higher scores (Supplementary Figure 3C). The redundancy removal can be identified in Supplementary Figure 3D as the scatter plot is less crowded and the maximum rank across the dataset is 60, as opposed to 120. Supplementary Figure 3E shows the non-redundant set of re-scored IF-SitePred

predictions. This score distribution is wider, i.e., scores take values from a larger distribution of values, which might yield a more relevant scoring of pockets.

There is no clear difference between Supplementary Figure 3G-H, meaning that using PRANK to score PUResNet predictions does not alter the ranking of the predictions made within a protein. This makes sense, as only 10% of proteins present >1 predicted pocket. This new score, however, could help in the ranking of pockets across the dataset, and not just within a protein.

The distribution of scores does not change when removing the redundancy from DeepPocket$_{SEG}$ predictions (Supplementary Figure 3I-J), but the maximum rank goes from 200 to 140 indicating the decrease in total predictions. The score distributions of fpocket$_{PRANK}$ (Supplementary Figure 3N) and fpocket (Supplementary Figure 3O) are completely different which means the ranking of pockets, and therefore recall and precision might differ considerably between these two scoring schemes of the same predictions.

The score distributions of "$_{AA}$", "$_{SS}$" and "$_{PRANK}$" variants of PUResNet, PocketFinder^{+}, Ligsite^{+} and Surfnet^{+} are similar, suggesting that the number of pocket amino acids might dictate the order in which these pockets are reported Supplementary Figure 3P-X.

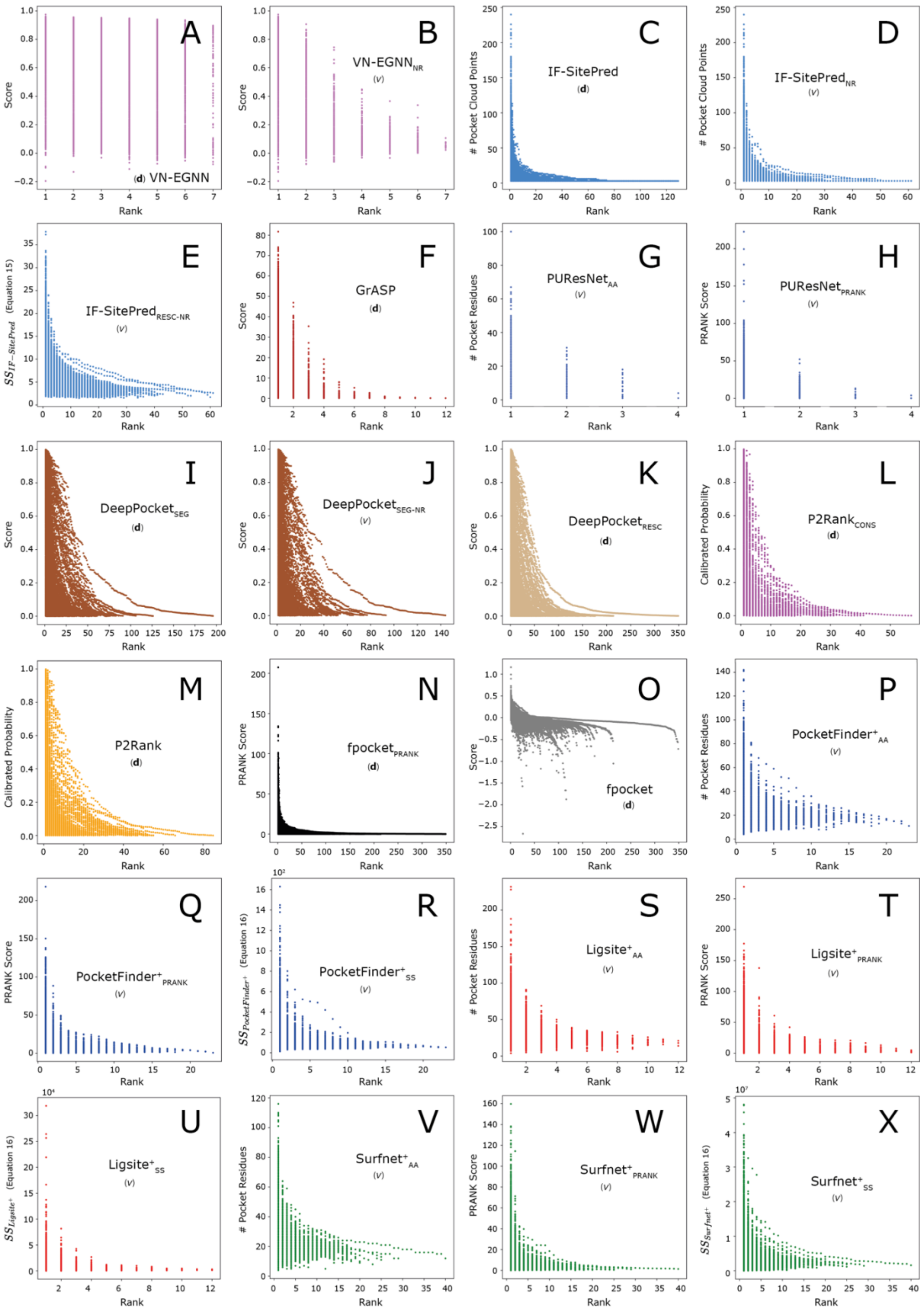

Supplementary Figure 3. Pocket score vs pocket ranking. (A) VN-EGNN reported pocket scores; **(B)** Non-redundant VN-EGNN predictions (VN-EGNN$_{NR}$); **(C)** Default IF-SitePred predictions are ranked based on the number of pocket cloud points; **(D)** Non-redundant variant

of IF-SitePred (IF-SitePred$_{NR}$); **(E)** Re-scored non-redundant IF-SitePred predictions (IF-SitePred$_{RESC\text{-}NR}$). Score is calculated as sum of squares of residue ligandability scores (Equation 15); **(F)** GrASP; **(G)** PUResNet does not score its pockets. PUResNet$_{AA}$. This variant uses the number of pocket amino acids as a score; **(H)** PRANK scored PUResNet pockets; **(I)** DeepPocket$_{SEG}$; **(J)** Non-redundant DeepPocket$_{SEG}$ predictions (DeepPocket$_{SEG\text{-}NR}$); **(K)** DeepPocket$_{RESC}$; **(L)** P2Rank$_{CONS}$; **(M)** P2Rank; **(N)** fpocket$_{PRANK}$; **(O)** fpocket. This distribution differs massively from the re-scored fpocket$_{PRANK}$ one; **(P)** PocketFinder^{+} does not report pocket scores, so the number of pocket residues is displayed for the PocketFinder$^{+}_{AA}$ variant; **(Q)** PocketFinder$^{+}_{PRANK}$; **(R)** PocketFinder$^{+}_{SS}$. This variant uses the pocket grid points' scores to calculate a pocket score by summing the squared scores (Equation 16); **(S)** Just like PocketFinder^{+}, Ligsite^{+} does not score pockets, Y-axis is number of pocket residues (Ligsite$^{+}_{AA}$); **(T)** Ligsite$^{+}_{PRANK}$; **(U)** Ligsite$^{+}_{SS}$; **(V)** Surfnet$^{+}_{AA}$; **(W)** Surfnet$^{+}_{PRANK}$; **(X)** Surfnet$^{+}_{SS}$. (**d**) and (*v*) indicate whether methods are default, or a variant generated in this work.

Supplementary Note 3: Recall Curves for Scoring and Ranking Variants

Supplementary Note 2 and Figure 3 demonstrate how removing redundancy from predictions can have a drastic effect in the ranking of the predictions, with VN-EGNN being the clearest example. We wanted to explore how redundancy removal affects recall as well as the effect that different pocket scoring schemes might have on recall for those methods that do not report a pocket score: PUResNet, PocketFinder^{+}, Ligsite^{+} and Surfnet^{+}.

Supplementary Figure 4A shows a significant +5.2% increase in recall when removing redundancy for VN-EGNN predictions (Recall = 46.1%), which corresponds to 346 extra predictions that fall within the top-N+2 after removing redundancy. An even stronger improvement can be observed for IF-SitePred (Supplementary Figure 4C), where a combination of redundancy removal and pocket re-scoring (Equation 13) results in a significant increase of +13.4% (Recall = 39.1%), corresponding to 901 added predictions falling in the considered top-N+2 predictions. Most of this change is due to the redundancy removal, as can be seen by the higher recall of IF-SitePred$_{NR}$. Scoring of PUResNet predictions using the number of pocket amino acids (PUResNet$_{AA}$) nor PRANK (PUResNet$_{PRANK}$) had no effect on the recall. This was expected as PUResNet predicts a single pocket in 90% of the cases, and therefore, there is no strong need for a score to sort predictions within a protein (Supplementary Figure 4G-I). Just like VN-EGNN and IF-SitePred, the recall of DeepPocket$_{SEG}$ benefits from redundancy removal, increasing by +5.7% (Supplementary Figure 4J) with a final recall = 49.4% (+377 pockets within ranking threshold).

For PocketFinder^{+}, Ligsite^{+} and Surfnet^{+}, none of the variants had a significant improvement in recall (Supplementary Figure 5). This is expected as these predict only a few non-redundant sites per protein, medians ranging 1-3 pockets per protein.

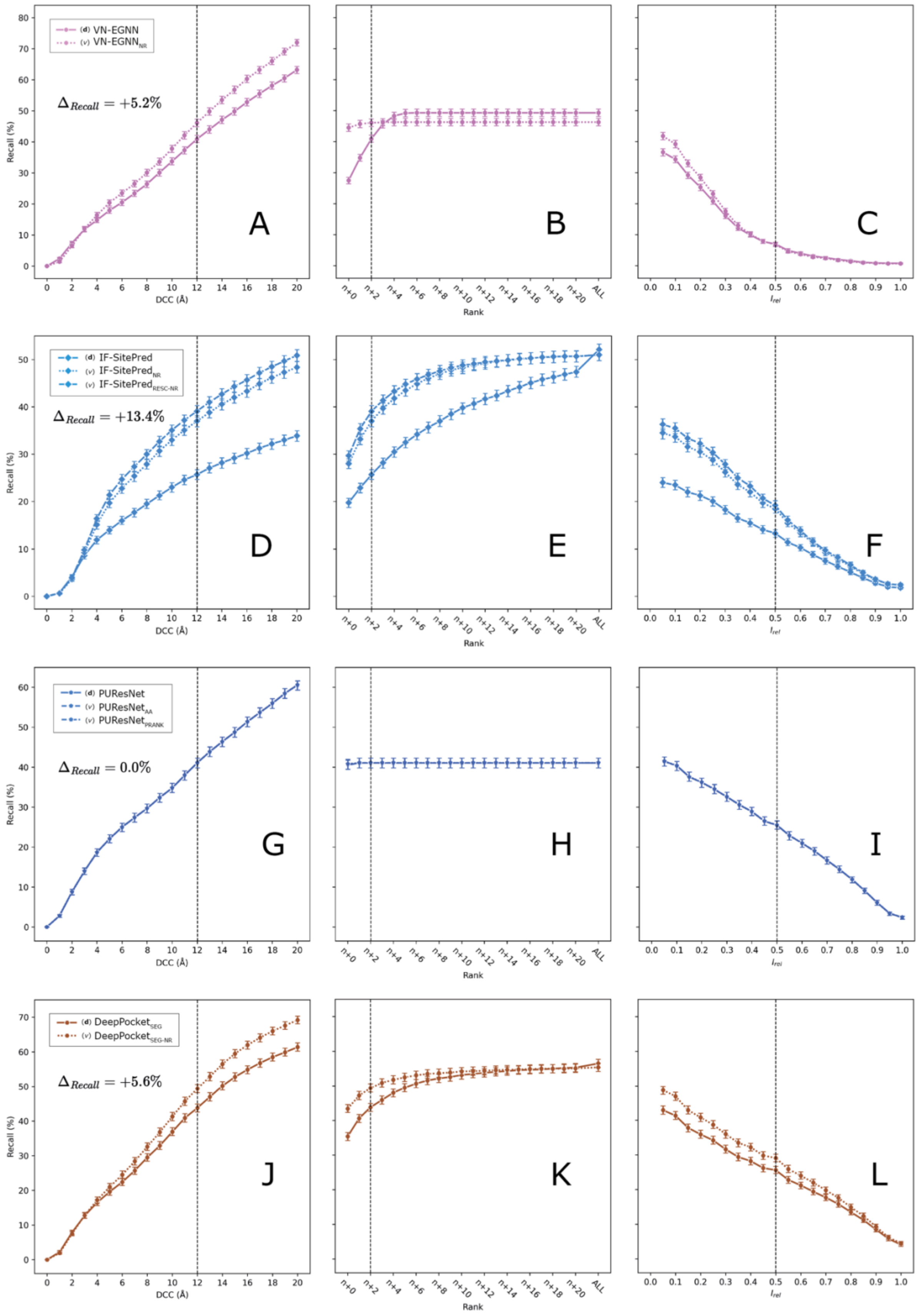

Supplementary Figure 4. Recall curves for method variants (I). Recall curves for different scoring and ranking variants of four machine-learning based methods that are either redundant:

VN-EGNN, IF-SitePred and $DeepPocket_{SEG}$, or do not score their pockets: PUResNet. Recall represents the proportion of observed ligand binding sites that are correctly identified by each method. For each method, three panels are shown to illustrate how recall changes as DCC, rank and I_{rel} threshold vary. In this last one I_{rel} is the criterion used to classify predictions. Dashed lines indicate the thresholds used as reference in this work: DCC = 12Å, rank = top-N+2, and I_{rel} = 0.5 (50% of residue overlap). For VN-EGNN and NR variant, recall vs DCC **(A)**, rank **(B)** and I_{rel} **(C)**; For IF-SitePred and variants, recall vs DCC **(D)**, rank **(E)** and I_{rel} **(F)**; For PUResNet and PRANK variant, recall vs DCC **(G)**, rank **(H)** and I_{rel} **(I)**. Rescoring pockets with PRANK has no effect on the recall; For $DeepPocket_{SEG}$ and NR variant, recall vs DCC **(J)**, rank **(K)** and I_{rel} **(L)**. Error bars show 95% CI of recall (proportion). (**d**) and (*v*) preceding method names indicate whether methods are default or variants, respectively. (**d**) and (*v*) indicate whether methods are default, or a variant generated in this work.

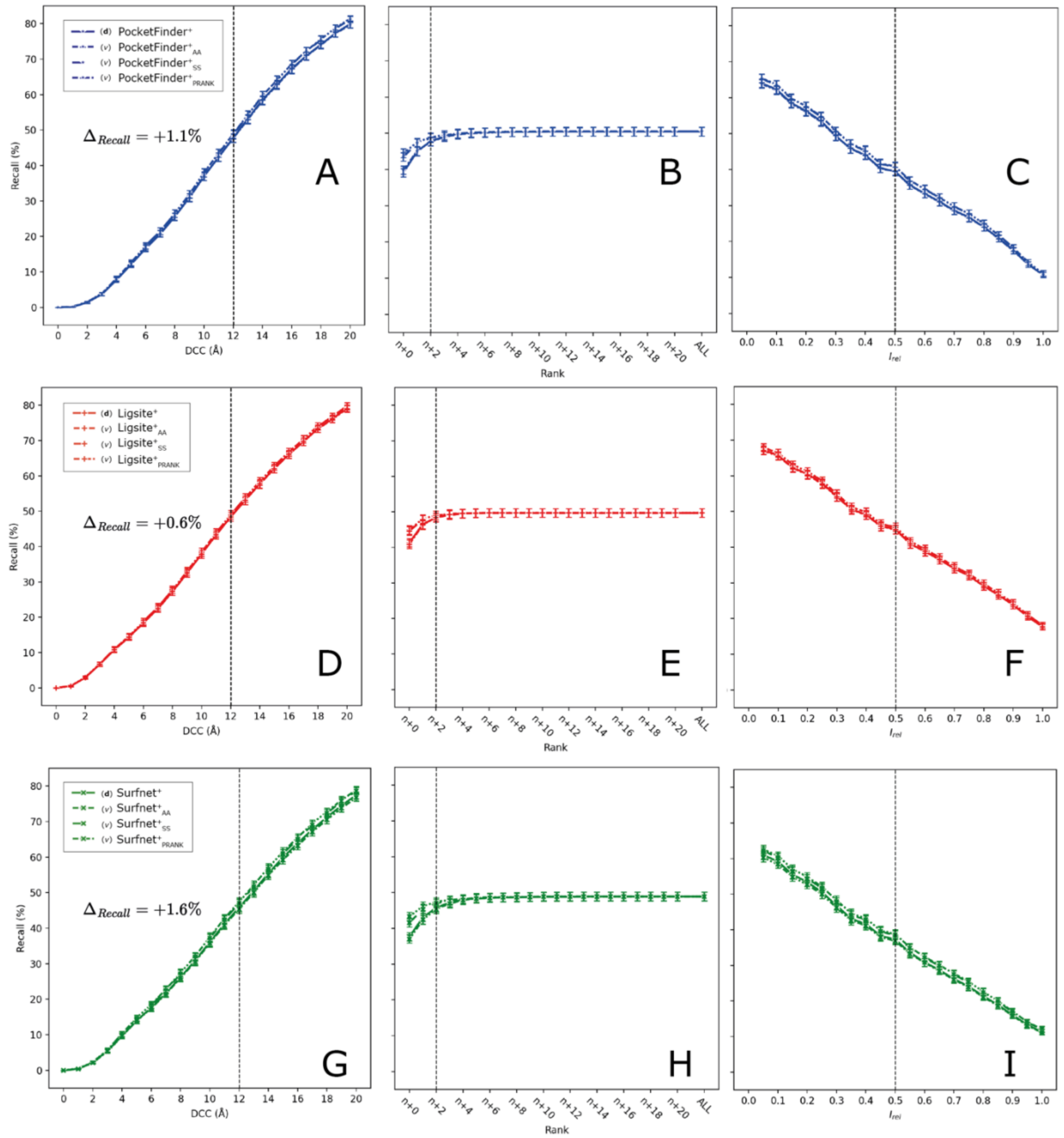

Supplementary Figure 5. Recall curves for method variants (II). Recall curves for different scoring and ranking variants for PocketFinder+, Ligsite+, and Surfnet+. For PocketFinder+ and variants, recall vs DCC **(A)**, rank **(B)** and I_{rel} **(C)**; For Ligsite+ and variants, recall vs DCC **(D)**, rank **(E)** and I_{rel} **(F)**; For Surfnet+ and variants, recall vs DCC **(G)**, rank **(H)** and I_{rel} **(I)**. Error bars show 95% CI of recall (proportion). (**d**) and (*v*) indicate whether methods are default, or a variant generated in this work.

Supplementary Note 4: ROC100 Curves for Scoring and Ranking Variants

There are no negative predictions, either True (TN) or False (FN) in the context of ligand binding site prediction at the pocket level and accordingly, standard ROC/AUC curves cannot be obtained. Only positives are predicted (sites). FN can be obtained by examining the observed pockets that are not predicted, but there are not scores for them. ROC100 curves provide an alternative to observe the relationship between True Positives (TP) and False Positives (FP). Predictions for each method across the whole test dataset, LIGYSIS, are sorted based on the pocket scores, and cumulative TP and FPs are counted until a certain number of FP is reached, in this case, 100. This visualisation provides insight into how well high-scoring predictions match the ground truth. A higher number of TP at FP = 100 indicates that the high scoring pockets recapitulate well the ground truth, whereas a low number indicates that the high scoring pockets do not match with the observed data, given the used thresholds of DCC ≤ 12Å. It is important to understand that the FP in this context might not always be incorrect predictions, but might be binding sites that are not considered in our ground truth dataset, that is comprised by biologically relevant protein-ligand interactions as defined in BioLiP [16], or relevant sites that simply have not been experimentally determined yet. It is also important to contextualise this metric with success rate, or recall (top-N+2), i.e., how many of the observed sites are predicted by each method given the above-mentioned threshold, as well as a rank threshold: top-N+2. A method might present a high number of TP within the first 100 FP yet have a low recall overall. Supplementary Figure 6 explores how ROC100 changes for the non-redundant "$_{NR}$" and re-scored "$_{AA}$", "$_{SS}$" and "$_{PRANK}$" sets of VN-EGNN, IF-SitePred, PUResNet, DeepPocket$_{SEG}$, PocketFinder^{+}, Ligsite^{+} and Surfnet^{+}.

Supplementary Figure 6A illustrates how redundancy can be misleading and overestimate the performance of VN-EGNN. Removing redundancy results in $\Delta_{TP} = -273$ (TP = 1,028). This is because redundant predictions by VN-EGNN are very close in space and present very similar scores (Supplementary Figure 3A). Because of this, in the redundant default set of predictions, multiple TP counts are being added for predictions of the same observed pocket. Even with redundancy removed, VN-EGNN reached 1,028

TP for the first 100FP, indicating that the non-redundant higher scoring pockets recapitulate well the observed data.

There is no difference between IF-SitePred and IF-SitePred$_{NR}$ (curves overlap completely), which indicates that despite the redundancy in predictions by this method, its scoring scheme can sort sites in a meaningful manner. Let us consider multiple proteins with redundant predictions for IF-SitePred. The scoring scheme allows for the top-1 site of each of these proteins to rank above any of the other redundant predictions of the other proteins. The re-scored and non-redundant set of IF-SitePred predictions results in a Δ_{TP} = +285 (TP = 1,246), indicating that IF-SitePred could benefit from a more sophisticated scoring scheme, rather than the number of cloud points per binding site (Supplementary Figure 6B).

Supplementary Figure 6C shows how important it is to score pocket predictions. PUResNet does not score its predictions. For this reason, within a protein, pockets have been ranked based on the order they are reported. When sorting across the whole dataset, pockets with the same ID or rank where randomly shuffled. A massive increase in TP can be observed when simply sorting by the number of pocket residues and using PRANK to score this pockets provides an even larger increment in TP (Δ_{TP} = +563) (TP = 1,097). An application of this could be running PUResNet on a list of potential drug target proteins. It would add great value to be able to rank the predictions among the targets to decide on a target.

The curve does not change much for DeepPocket$_{SEG}$, (Δ_{TP} = −27) (TP = 643), indicating that despite the segmentation module of DeepPocket might result in overlapping pockets, their scoring scheme is robust. It is important to consider that the pocket score results from re-scoring the fpocket candidates, which are not redundant. The redundancy in DeepPocket$_{SEG}$ is therefore unrelated to their scoring scheme. These results suggest that there is a big difference between fpocket candidates and extracted DeepPocket pockets, and it might not be appropriate to consider the score of the former for the latter (Supplementary Figure 6D).

For the last three methods, PocketFinder^{+}, Ligsite^{+} and Surfnet^{+}, the results agree in that simply using the number of pocket amino acids results in the maximum TP for 100 FP: Δ_{TP} = +114 (TP = 178) (Supplementary Figure 6E), Δ_{TP} = +44 (TP = 159) (Supplementary Figure 6F) and Δ_{TP} = +247 (TP = 308) (Supplementary Figure 6G). This is surprising, as sum

of squares “$_{SS}$” and PRANK scoring schemes have worked better for other methods. This result might be related to the fact that pockets predicted by these three methods tend to be larger than those predicted by other methods.

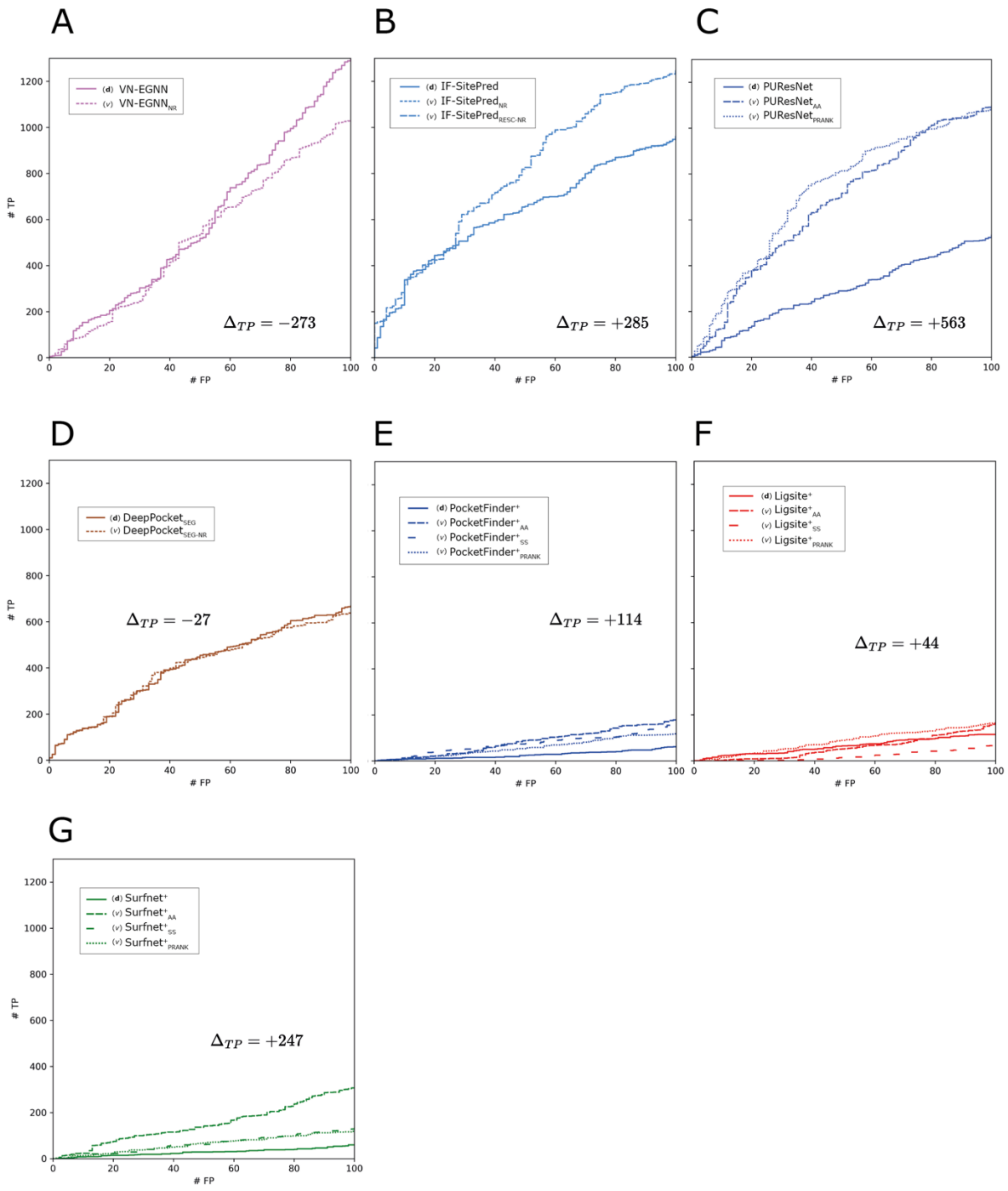

Supplementary Figure 6. ROC100 curves for different scoring and ranking variants. For each method, predicted pockets across the whole dataset, i.e., all LIGYSIS proteins, are ranked by their score. This way, pockets with the highest scores will be at the top of the list, whereas pockets with the lowest scores will be at the bottom. Note that this ranking will not correspond to ranking

pockets across the dataset by their rank, as a pocket ranked #2, #3 or lower could have a higher score than a pocket #1 of another protein. Each method has a colour assigned, and each scoring variant its own line style. **(A)** VN-EGNN$_{NR}$ presents 273 fewer true positives (TP) when 100 false positives (FP) are reached compared to VN-EGNN. This is because redundant predictions for the same site are being counted as multiple TPs; **(B)** IF-SitePred$_{RESC\text{-}NR}$ reaches +285 TPs than default IF-SitePred; **(C)** Using PRANK to score and sort (unscored) PUResNet predictions increases the number of TPs for 100 FPs by 563; **(D)** 27 fewer TP for DeepPocket$_{SEG\text{-}NR}$, again a consequence of removing redundant predictions; **(E)** Using the number of pocket amino acids (PocketFinder$^{+}_{AA}$) increase TPs by +114; **(F)** Ligsite$^{+}_{AA}$ adds 44 TPs; **(G)** Likewise, with the ranking of Surfnet$^{+}_{AA}$, +247 are gained relative to unscored Surfnet^{+} predictions. (**d**) and (*v*) indicate whether methods are default, or a variant generated in this work.

Supplementary Note 5: Precision curves for scoring and ranking variants

For the same reason as why ROC/AUC curves cannot be calcualted for ligand binding site prediciton (at the pocket level), precision-recall (PR)/AUC curves cannot be either, as False Negatives (FN) are not predicted, and therefore not scored. Nevertheless, precision, as the ratio of TP/TP+FP, can be measured. For this, as it was done for ROC100, all predictions for a method were sorted by pocket score, and precision calculated as more predictions are considered.

Supplementary Figure 7 portrays the precision curve for the top-1,000 predictions for the non-redundant and re-scored variants for VN-EGNN, IF-SitePred, PUResNet, $\text{DeepPocket}_{\text{SEG}}$, PocketFinder^{+}, Ligsite^{+} and Surfnet^{+}.

There is no significant ($p > 0.05$) change in precision between VN-EGNN and $\text{VN-EGNN}_{\text{NR}}$ within the first 1,000 predictions, precision = 91.5% (Supplementary Figure 7A). The same can be said for IF-SitePred with a precision = 94.3% (Supplementary Figure 7B). Using PRANK to score PUResNet pockets results in a significant +11.7% increase in precision of the top-1,000 predictions (precision = 93.3%) (Supplementary Figure 7C). $\text{DeepPocket}_{\text{SEG-NR}}$, as the other redundant methods, does not experience a significant change in precision as redundancy is removed (precision = 81.6%) (Supplementary Figure 7D). For PocketFinder^{+}, Ligsite^{+} and Surfnet^{+}, using the number of pocket amino acids results, "$_{\text{AA}}$", in a precision increase of +23.3% (precision = 65.3%), (Supplementary Figure 7E), +16.5% (precision = 68.8%) (Supplementary Figure 7F) and 29.1% (precision = 68.8%) (Supplementary Figure 7G), respectively.

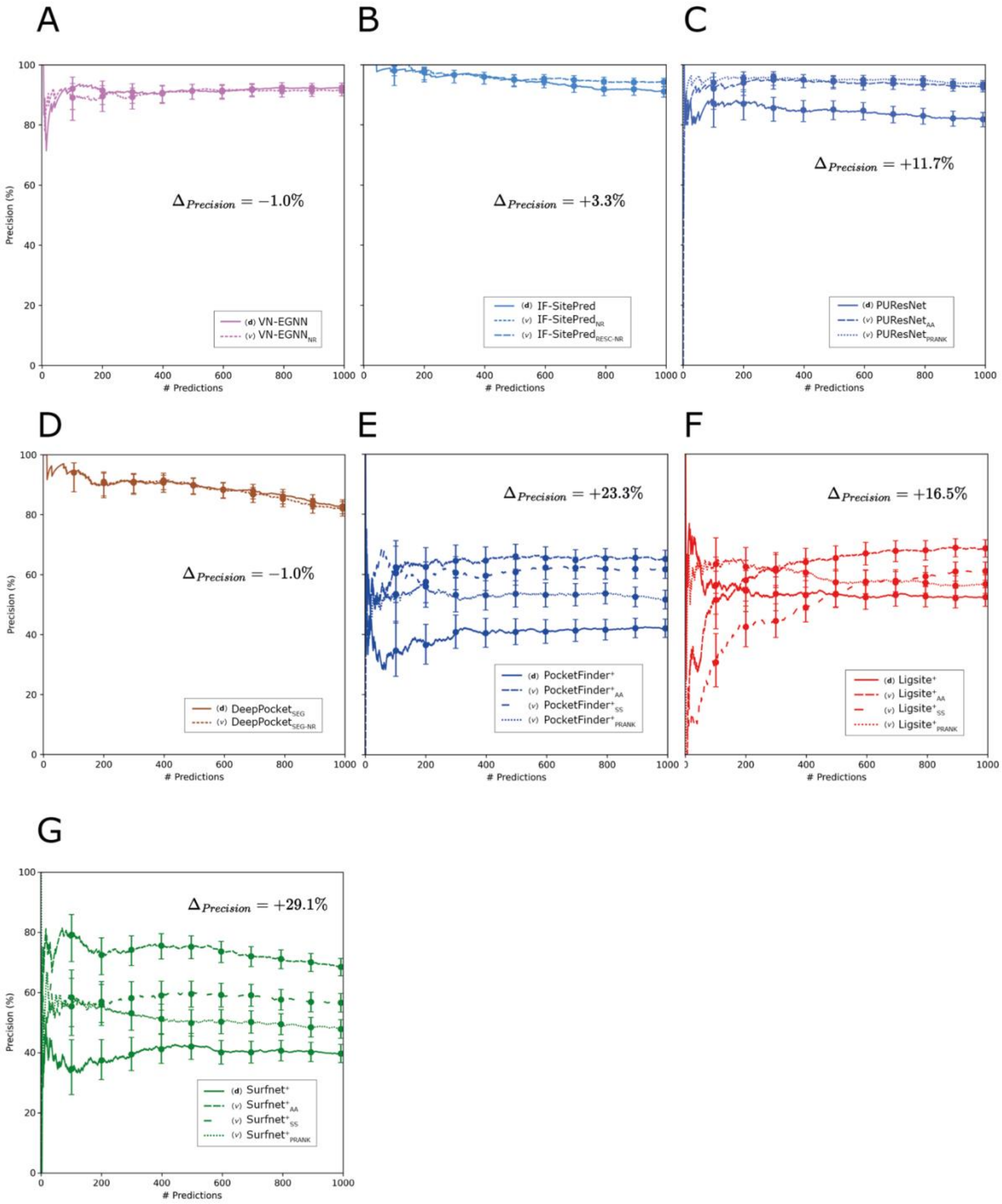

Supplementary Figure 7. Precision (%) for the top-scoring 1,000 predictions, Precision$_{1K}$. For each method, predicted pockets across the whole LIGYSIS set, are ranked by their score. This way, pockets with the highest scores will be at the top of the list, whereas pockets with the lowest scores will be at the bottom. Each method has a colour assigned, and each scoring variant its own line style. $\Delta_{Precision}$ indicates the difference in precision between the selected method variant and the default one. **(A)** VN-EGNN; **(B)** IF-SitePred; **(C)** PUResNet; **(D)** DeepPocket$_{SEG}$; **(E)** PocketFinder^{+}; **(F)** Ligsite^{+}; **(G)** Surfnet^{+}. Error bars indicate 95% CI of the precision (proportion)

and are displayed every 100 predictions. (**d**) and (*v*) indicate whether methods are default or a variant generated in this work.

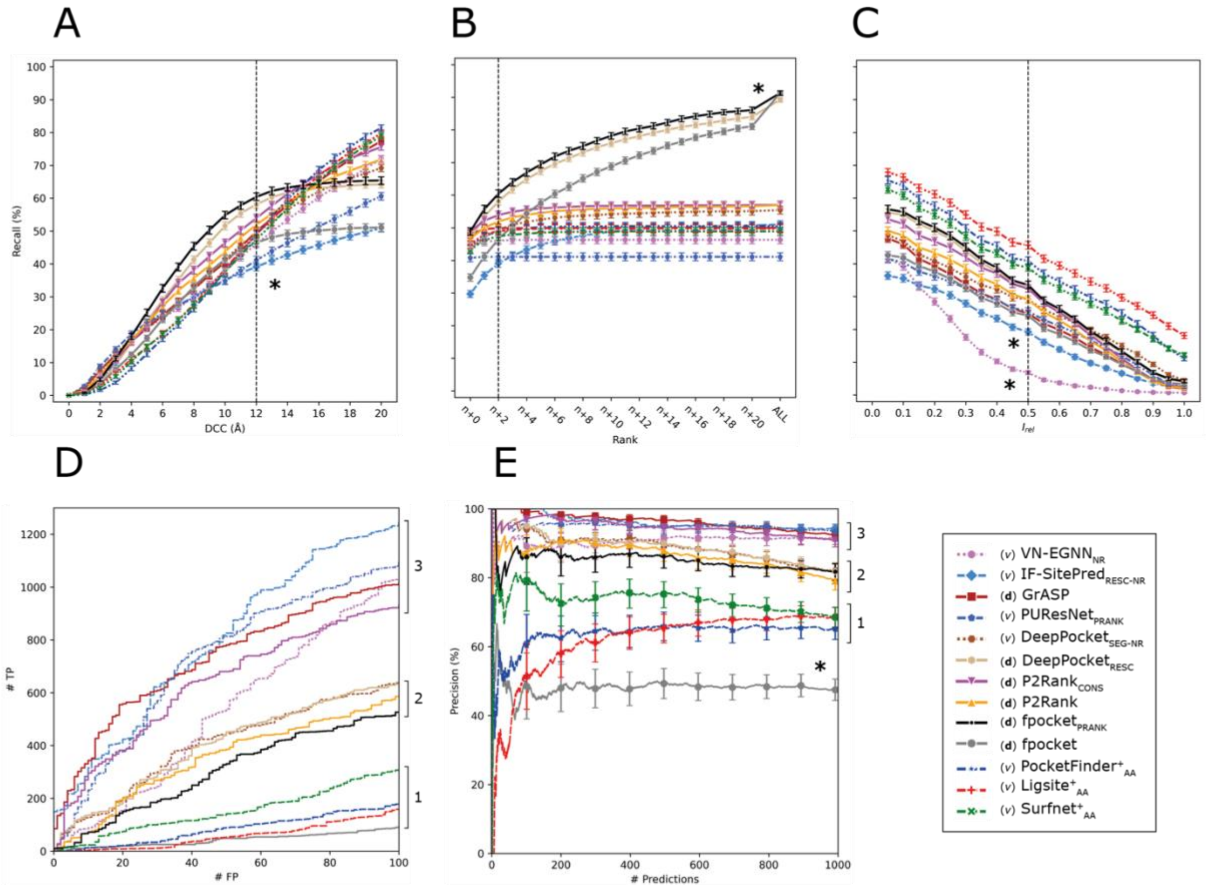

Supplementary Figure 8. Ligand binding site prediction benchmark at the pocket level. For methods with scoring or non-redundant variants, only the top-performing, i.e., highest top-N+2 recall, variant of each method is drawn on this figure, e.g., VN-EGNN$_{NR}$, IF-SitePred$_{RESC-NR}$, or DeepPocket$_{SEG-NR}$, indicated by a (*v*) next to their name. Default modes are represented for all other methods, indicated by (**d**). **(A)** Recall, percentage of observed sites that are correctly predicted by a method according to a DCC threshold and ranking within the top-N+2 predictions. Reported recall on Table 4 corresponds to DCC = 12Å; **(B)** Recall using DCC = 12Å but considering increasing rank thresholds. *ALL* represents the maximum recall of a method, obtained by considering all predictions, regardless of their rank or score; **(C)** Recall curve considering top-N+2 pockets and using I_{rel} as a criterion; **(D)** ROC100 curve (cumulative TP against cumulative FP until 100 FP are reached); **(E)** Precision curve for the top-1,000 predictions of each method across the LIGYSIS dataset. Error bars represent 95% CI of the recall (A-C) and precision (E), which are 100 × proportion. Numbers at the right of the panels indicate groups or blocks of methods that perform similarly for each metric. Asterisks (*) indicate outlier methods, or methods that perform very differently than the rest.

Method	% Recall$_{top-N}$	% Recall$_{top-N+2}$	% Recall$_{max}$	% Precision$_{1K}$	# TP$_{100\ FP}$	% RRO	% RVO
(*v*) VN-EGNN$_{NR}$	44.5 (#7)	46.1 (#11)	46.3 (#11)	91.5 (#4)	1028 (#3)	**31.6^{-} (#11)**	**26.7^{-} (#11)**
(*v*) IF-SitePred$_{RESC-NR}$	**29.7^{-} (#12)**	**39.1^{-} (#13)**	51.0 (#6)	**94.3^{+} (#1)**	**1246^{+} (#1)**	49.3 (#10)	43.7 (#9)
(**d**) GrASP	48.0 (#2)	49.9 (#5)	50.0 (#8)	92.5 (#3)	1017 (#4)	54.5 (#7)	59.8 (#6)
(*v*) PUResNet$_{PRANK}$	40.8 #10)	41.1 (#12)	**41.1^{-} (#12)**	93.3 (#2)	1094 (#2)	61.0 (#4)	63.9 (#4)
(*v*) DeepPocket$_{SEG-NR}$	43.4 (#8)	49.4 (#6)	55.4 (#5)	81.6 (#7)	643 (#6)	58.4 (#5)	61.3 (#5)
(**d**) DeepPocket$_{RESC}$	46.6 (#4)	58.1 (#2)	89.3 (#2)	81.7 (#6)	637 (#7)	52.6 (#9)	38.2 (#10)
(**d**) P2Rank$_{CONS}$	**48.8^{+} (#1)**	53.9 (#3)	57.0 (#3)	90.7 (#5)	932 (#5)	56.4 (#6)	43.8 (#8)
(**d**) P2Rank	46.7 (#3)	51.9 (#4)	57.0 (#4)	79.2 (#8)	586 (#8)	54.4 (#8)	58.2 (#7)
(**d**) fpocket$_{PRANK}$	**48.8^{+} (#1)**	**60.4^{+} (#1)**	**91.3^{+} (#1)**	81.7 (#6)	526 (#9)	52.6 (#9)	38.2 (#10)
(**d**) fpocket	38.8 (#11)	46.5 (#10)	**91.3^{+} (#1)**	**47.3^{-} (#12)**	**94^{-} (#13)**	52.6 (#9)	38.2 (#10)
(*v*) PocketFinder$^{+}_{AA}$	44.5 (#6)	48.9 (#8)	50.5 (#7)	65.3 (#11)	178 (#11)	72.3 (#2)	75.9 (#2)
(*v*) Ligsite$^{+}_{AA}$	44.9 (#5)	49.0 (#7)	49.7 (#9)	68.8 (#9)	159 (#12)	**77.6^{+} (#1)**	**77.0^{+} (#1)**
(*v*) Surfnet$^{+}_{AA}$	43.3 (#9)	47.4 (#9)	48.9 (#10)	68.6 (#10)	308 (#10)	71.7 (#3)	72.0 (#3)

Supplementary Table 2. Summary table of ligand binding site prediction benchmark at the pocket level. Only the top-performing, i.e., highest (top-N+2) recall, variant of each method appear on this table, e.g., VN-EGNN$_{NR}$, IF-SitePred$_{RESC-NR}$ or DeepPocket$_{SEG-NR}$, instead of their default modes. Recall (%) considering top-N, N+2 and *all* predictions without taking rank into consideration, i.e., maximum recall. Precision (%) of the method for the top-1,000 scored predictions. Number of TP reached for the first 100 FP. Mean relative residue overlap (RRO) for those sites correctly predicted and mean relative volume overlap (RVO) only for correctly predicted sites that have a volume, i.e., are pockets or cavities, and not exposed sites, which don't have a volume. These last two metrics are also percentages and represent the overlap in residues and volume relative to the observed site. Within each cell, the numbers following a dash (#) indicate the rank of each method according to the metric in the column. The best and worst performing methods are indicated with bold font and "+" and "−" respectively. (**d**) and (*v*) in the first column indicate whether these are default or a method variant.

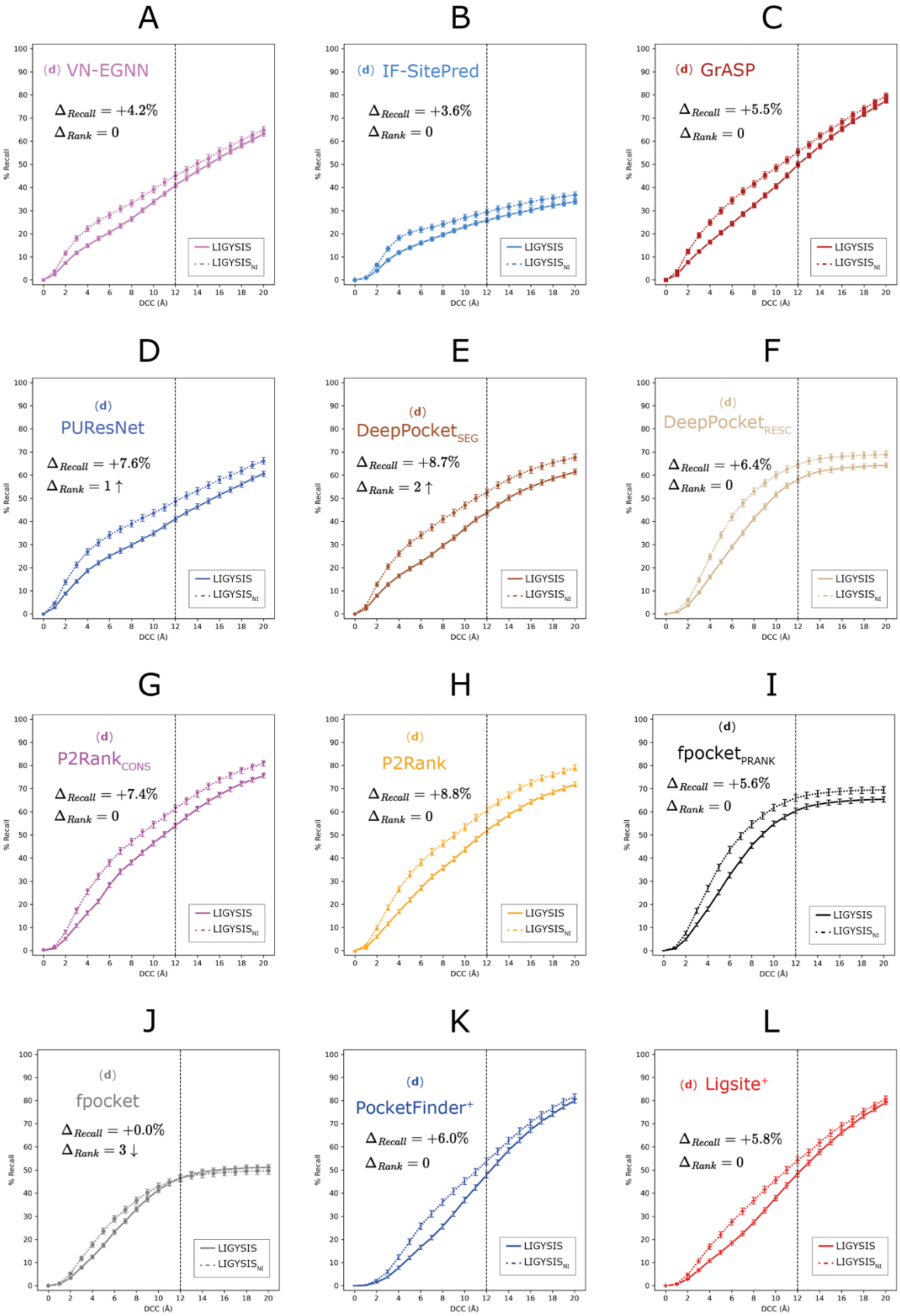
A
(d) VN-EGNN
$\Delta_{Recall} = +4.2\%$
$\Delta_{Rank} = 0$
B
(d) IF-SitePred
$\Delta_{Recall} = +3.6\%$
$\Delta_{Rank} = 0$
C
(d) GrASP
$\Delta_{Recall} = +5.5\%$
$\Delta_{Rank} = 0$
D
(d) PUResNet
$\Delta_{Recall} = +7.6\%$
$\Delta_{Rank} = 1 \uparrow$
E
(d) $DeepPocket_{SEG}$
$\Delta_{Recall} = +8.7\%$
$\Delta_{Rank} = 2 \uparrow$
F
(d) $DeepPocket_{RESC}$
$\Delta_{Recall} = +6.4\%$
$\Delta_{Rank} = 0$
G
(d) $P2Rank_{CONS}$
$\Delta_{Recall} = +7.4\%$
$\Delta_{Rank} = 0$
H
(d) P2Rank
$\Delta_{Recall} = +8.8\%$
$\Delta_{Rank} = 0$
I
(d) $fpocket_{PRANK}$
$\Delta_{Recall} = +5.6\%$
$\Delta_{Rank} = 0$
J
(d) fpocket
$\Delta_{Recall} = +0.0\%$
$\Delta_{Rank} = 3 \downarrow$
K
(d) PocketFinder⁺
$\Delta_{Recall} = +6.0\%$
$\Delta_{Rank} = 0$
L
(d) Ligsite⁺
$\Delta_{Recall} = +5.8\%$
$\Delta_{Rank} = 0$
LIGYSIS
$LIGYSIS_{NI}$
% Recall
DCC (Å)

M

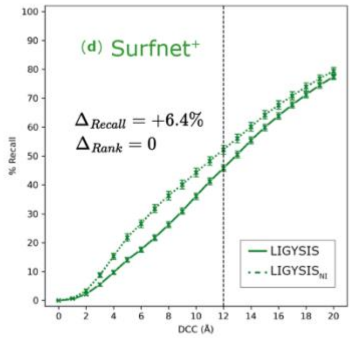

Supplementary Figure 9. Change in % Recall when benchmarking on the subset of LIGYSIS with no ions, LIGYSIS$_{NI}$. % Recall is calculated considering top-N+2 pockets at DCC = 12Å and default method variants on a set of LIGYSIS binding sites containing at least one non-ion ligand, N = 4,141/6,882 (60%). Solid lines indicate recall curve on LIGYSIS and dashed lines for LIGYSIS$_{NI}$. The relative change in % Recall and Rank are indicated by Δ_{Recall} and Δ_{Rank}. These changes are relative to LIGYSIS metrics. All methods except for fpocket present an increase in % Recall when removing ion binding sites. This is expected ad none of the methods are trained on ion sites. However, ion sited were kept on the main benchmark to challenge and test the limits of the methods. fpocket does not improve recall at this DCC threshold, but it does at more stringent thresholds. Most methods rank the same except fpocket that goes down three positions, PUResNet that climbs one position and DeepPocket$_{SEG}$ that climbs two. **(A)** VN-EGNN; **(B)** IF-SitePred; **(C)** GrASP; **(D)** PUResNet; **(E)** DeepPocket$_{SEG}$; **(F)** DeepPocket$_{RESC}$; **(G)** P2Rank$_{CONS}$; **(H)** P2Rank; **(I)** fpocket$_{PRANK}$; **(J)** fpocket; **(K)** PocketFinder^{+}; **(L)** Ligsite^{+}; **(M)** Surfnet^{+}. These results originate from default methods, indicated by (**d**) preceding method names.

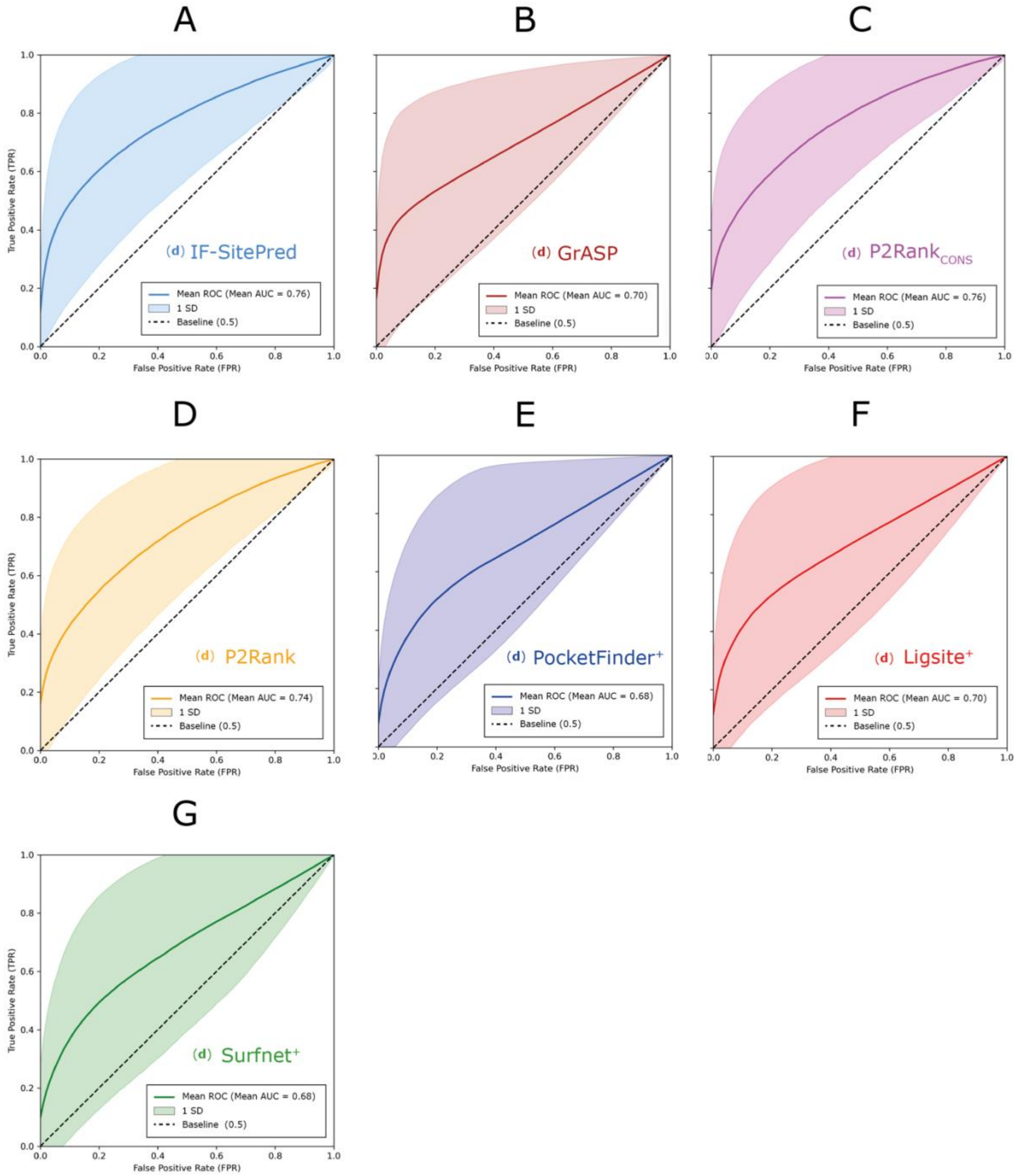

Supplementary Figure 10. Variation in ROC curve and AUC across LIGYSIS proteins. For each of the methods that report or for which residue ligandability scores were calculated, a ROC curve was calculated for each of the 2,775 protein chains in the LIGYSIS set and AUC calculated. The curves plotted here represent the mean ROC curve for each method. These are obtained by averaging the TPR for each FPR interval across proteins. Shaded area represents one standard deviation (1 SD) from the mean ROC curve. Reported AUC is the mean AUC calculated by averaging the AUC for the 2,775 ROC curves obtained. Baseline AUC is random chance (AUC = 0.5). **(A)** IF-SitePred; **(B)** GrASP; **(C)** P2Rank$_{CONS}$; **(D)** P2Rank; **(E)** PocketFinder^{+}; **(F)** Ligsite^{+}; **(G)** Surfnet^{+}. These results originate from default methods, indicated by (**d**) preceding method names.

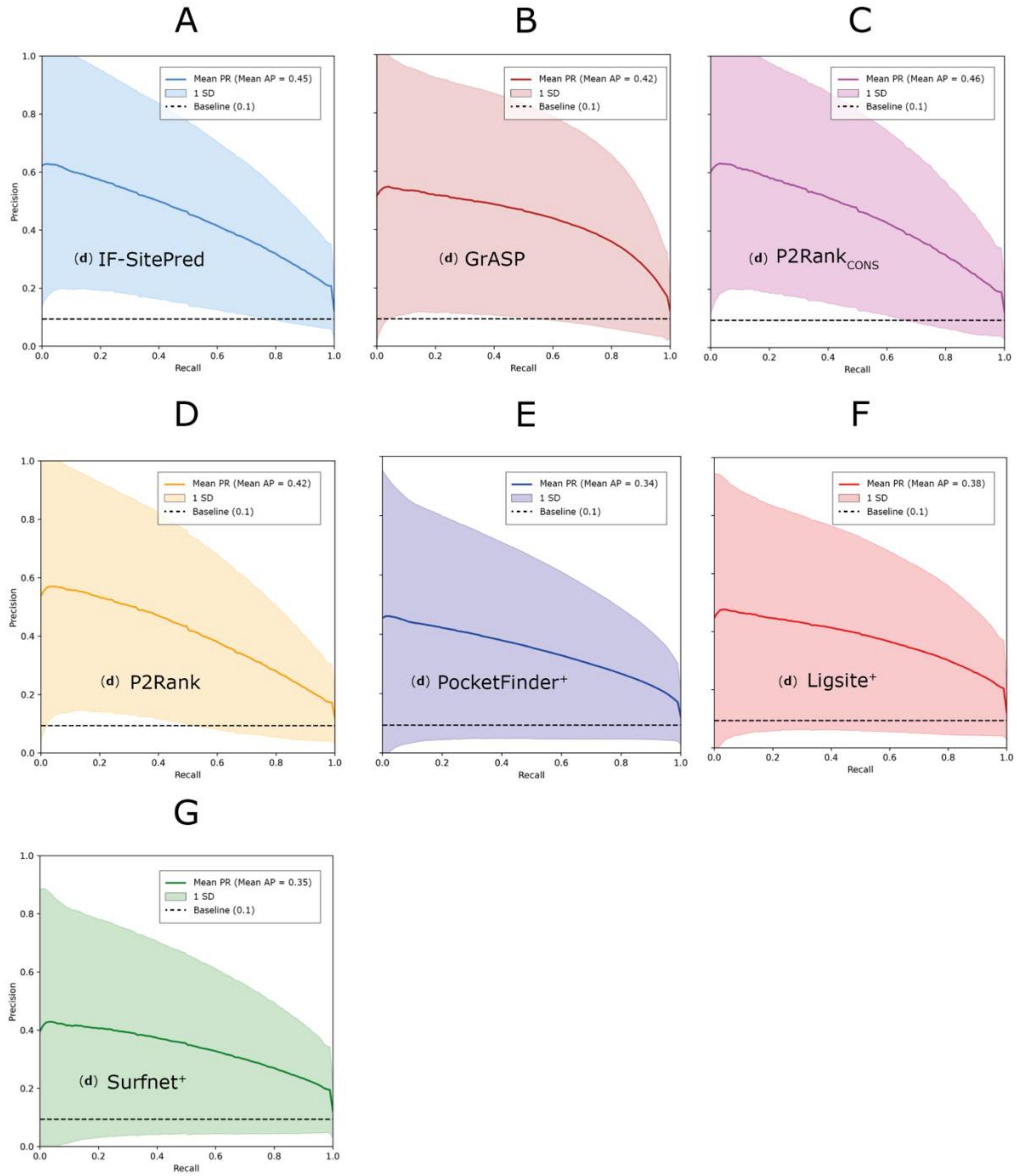

Supplementary Figure 11. Variation in PR curve and AP across LIGYSIS proteins. For each of the methods that report or for which residue ligandability scores were calculated, a precision-recall (PR) curve was calculated for each of the 2,775 protein chains in the LIGYSIS set and average precision (AP) calculated. The curves plotted here represent the mean PR curve for each method. These are obtained by averaging the precision for each recall interval across proteins. Shaded area represents one standard deviation (1 SD) from the mean PR curve. Reported AP is the mean AP calculated by averaging the AP for the 2,775 PR curves obtained. Baseline AP is the proportion of true positives, i.e., observed ligand-binding residues (AP = 0.1). **(A)** IF-SitePred; **(B)** GrASP; **(C)** P2Rank$_{CONS}$; **(D)** P2Rank; **(E)** PocketFinder^{+}; **(F)** Ligsite^{+}; **(G)** Surfnet^{+}. These results originate from default methods, indicated by (**d**) preceding method names.

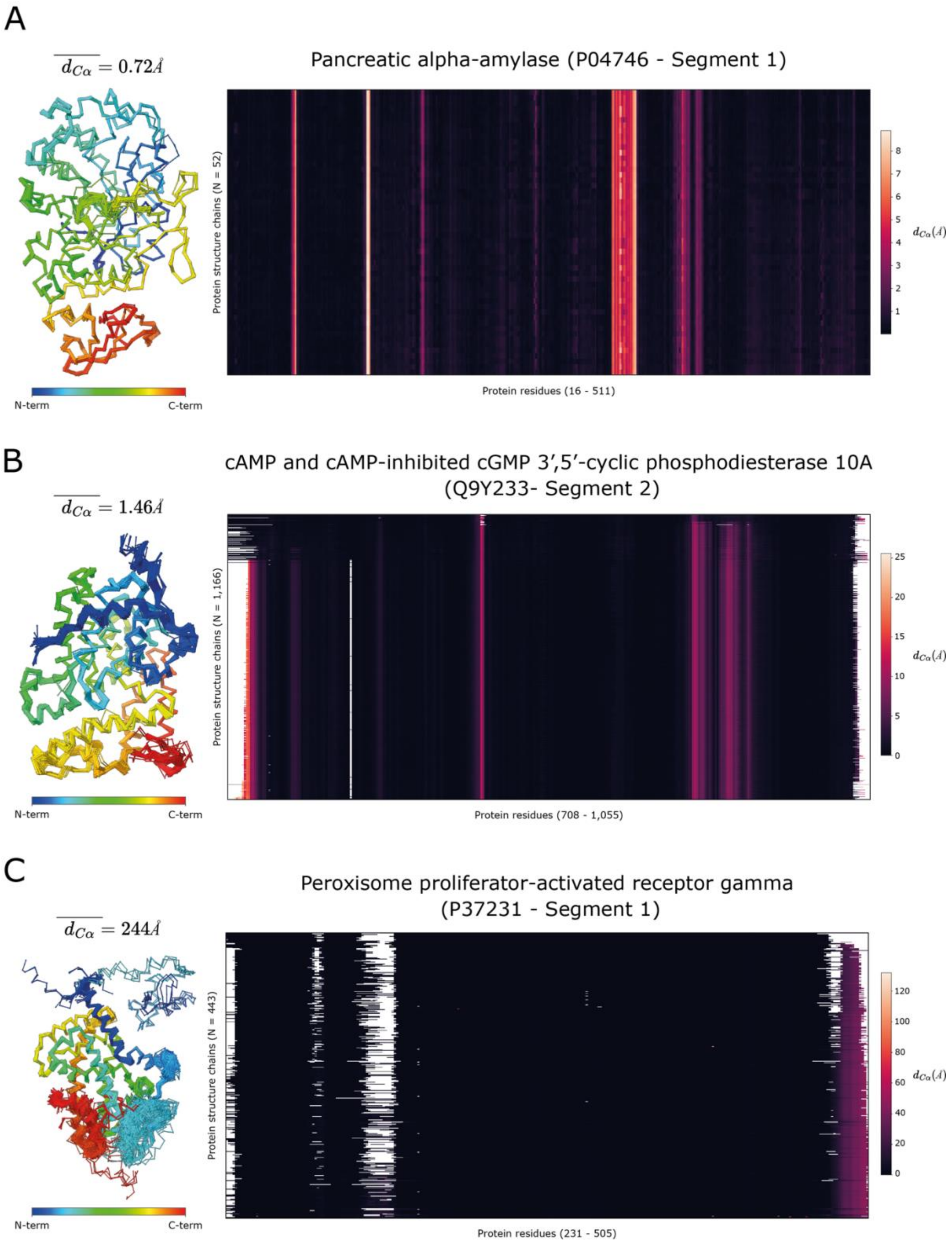

Supplementary Figure 12. Protein chains superposition. Transformation matrices obtained from the PDBe-KB were utilised to structurally align protein chains. Three examples are depicted here. For each one, superposed chain trace (Cα atoms) are shown in sticks and coloured using the rainbow scheme from N- to C-terminus and average distance across residues from the aligned chains to the PDBe-KB-defined representative chain is reported as $\overline{d_{C\alpha}}$ (Å) (left). On the right the superposition is visualised with a heatmap. Protein chain residues are on the X axis and aligned protein chains on the Y axis. Protein chains are sorted

by the average distance to the representative chain, so more dissimilar chains are on the bottom. Cells are coloured based on their $\overline{d_{C\alpha}}$ using the *rocket* colour scheme. White cells represent residues present in the representative chain but not the aligned one, i.e., discontinuities or chain breaks. Residues with very high > 20Å represent alternative locations that were not transformed correctly. **(A)** Pancreatic alpha-amylase with 52 superposed chains; **(B)** cAMP and cAMP-inhibited cGMP 3',5'-cyclic phosphodiesterase 10A with 1,166 chains; **(C)** Peroxisome proliferator-activated receptor gamma with 443 chains.

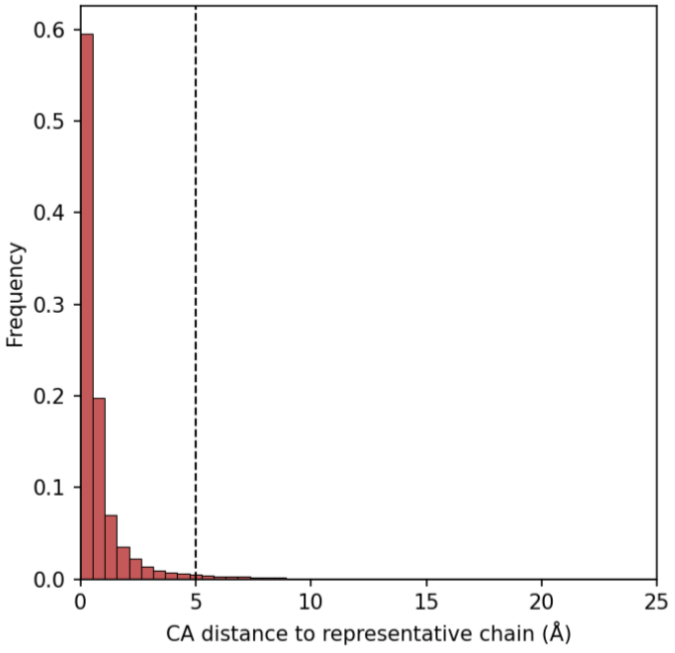

Supplementary Figure 13. Distance to representative chain for ligand binding residues. This histogram represents the distribution of the average Cα distance across transformed chains to the representative chain for 74,536 ligand binding residues across the 2,478 segments that present more than one chain. Black dash line indicates 5Å. 95% of ligand binding residues are within 5Å of the representative structure in average across chains. This demonstrates that the variation in the Cα trace for ligand binding residues across different structures of the same protein is very small.

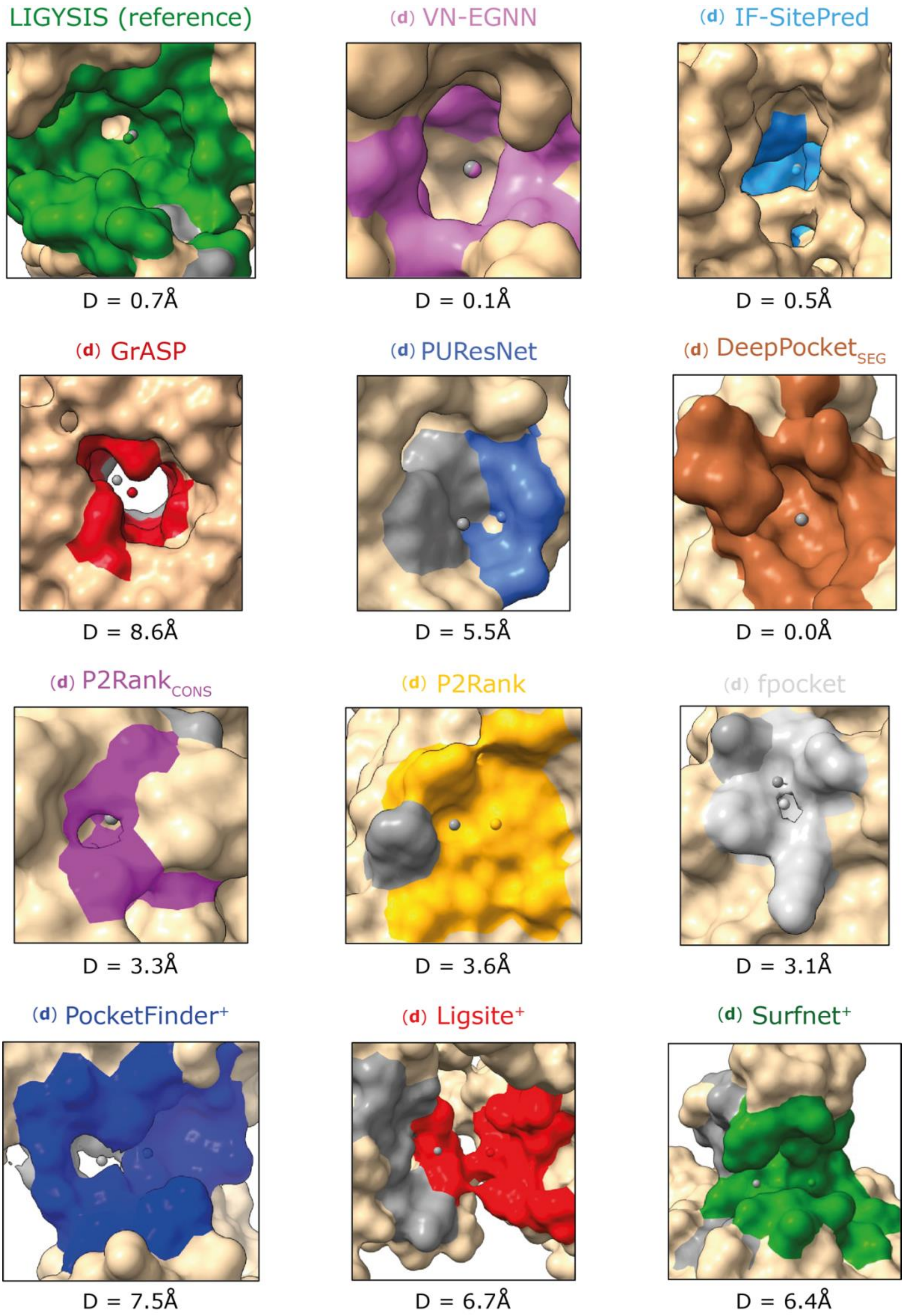

Supplementary Figure 14. Closest predicted pockets for each method. For each method, the two closest predicted pockets across all protein chains are shown. This is the pair of pockets with

the minimum Euclidean distance between their centroids. Protein surface is coloured in tan. The larger pocket (more residues) and centroid is coloured in the method colour, and the other in grey. A distance threshold of D = 5Å was selected to determine whether a pocket prediction was redundant. LIGYSIS, VN-EGNN, IF-SitePred and DeepPocket clearly differ from other methods presenting distances < 1Å. (**d**) next to the method names indicate these refer to default methods and not variants.

Supplementary Note 6: empirical determination of a DCC threshold

Most methods employ distance to any atom of the ligand (DCA) and a threshold of 4Å to consider a prediction as correct. Because of the way the LIGYSIS dataset has been curated, it is easier to use DCC, as our binding sites result of the clustering of multiple ligands, and not just a single ligand binding a protein. Despite DCC and DCA being different metrics, the same threshold of D = 4Å is used for both when benchmarking methods [2, 8, 10]. Supplementary Figure 15A shows the relation between DCC, and pocket residue overlap for the best pocket predictions, i.e., minimum inter-centroid Euclidean distance, for each observed pocket for each method. Across all methods, there are more than 15,000 predicted pockets with a DCC > 4Å and a residue overlap ≥ 50%. Setting the DCC threshold at 4Å would result in the *wrong* labelling of these predictions as "false positives". For this reason, we endeavoured into empirically establishing a more meaningful DCC threshold through the thorough visual inspection of predicted-observed pocket pairs. Supplementary Figure 15B suggests this threshold might be somewhere in between 10-15Å, where the proportion of pockets with I_{rel} ≥ 0.5 decreases until reaching 0.

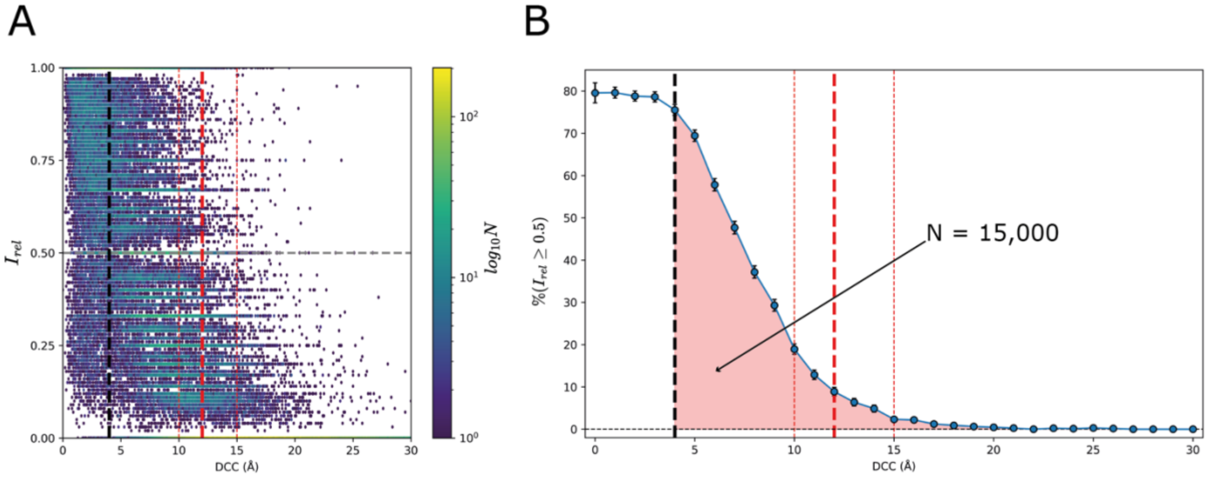

Supplementary Figure 15. I_{rel} vs DCC. (A) Hexagonal binned plot of I_{rel} (Y) vs DCC (X). Data points are grouped into hexagonal bins, and these are coloured by the number of data points within each bin using the *viridis* colour palette. Colour bar axis is in log scale. Black dashed lines indicate the literature consensus DCC = 4Å threshold and an arbitrary I_{rel} threshold of 0.5, i.e., coverage of half of the observed residues by the predicted pocket. Red lines delimit the likely location of a potentially more informative DCC thresholds; **(B)** Cumulative proportion of predicted pockets

with $I_{rel} \geq 0.5$ for each DCC 1Å interval. The commonly used threshold of DCC = 4Å would label >15,000 predictions with $I_{rel} \geq 0.5$ as false. Error bars indicate 95% CI of the proportion.

We set a hard threshold at D = 20Å and decided that based purely on distance, pockets with DCC > 20 would not be considered as correct predictions. For each DCC interval of 1Å, the pocket with the highest and lowest I_{rel} were inspected (Supplementary Figure 16). This initial visual inspection supported the hypothesis that a more meaningful DCC threshold is between 10-14Å. For the next step, only predicted-observed pocket pairs with minimal overlap ($I_{rel} < 0.25$) were considered. Starting at DCC = 10Å, and using unit intervals, the 100 farthest pockets were inspected for each interval, and the proportion of correct predictions was calculated as the number of pockets labelled as "correct" upon visual inspection divided by 100, i.e., %. For D = 10Å, 94% of pockets were correct (Supplementary Figure 17), 86% for D = 11Å (Supplementary Figure 18), 85% for D = 12Å (Supplementary Figure 19) and 66% for D = 13Å. Due to the considerable drop of correct pockets at D = 13Å, the final distance threshold was set at D = 12Å. Accordingly, predictions were considered as true positives if DCC ≤ 12Å (Supplementary Equation 1).

$$True\ Positive \iff (DCC \leq 12) \quad (1)$$

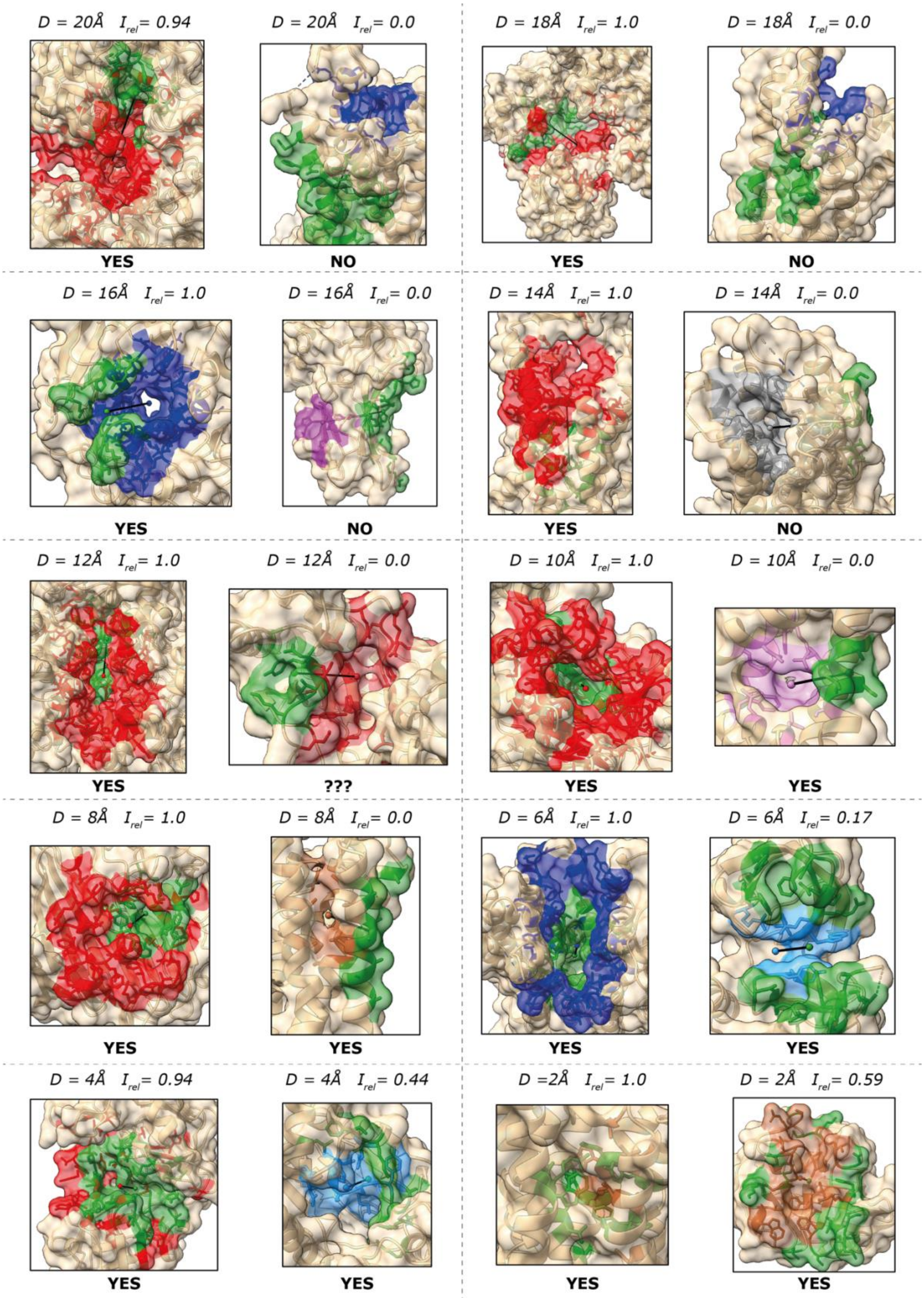

Supplementary Figure 16. Determination of DCC threshold. Highest and lowest-residue overlap predictions for each 2Å DCC unit interval. Observed LIGYSIS sites are coloured in green,

predicted pockets in other colours. D represents DCC and I_{rel} the relative intersection between predicted and observed pocket residues, i.e., proportion of observed site residues covered by predicted pocket residues. "**YES**" or "**NO**" labels indicate whether we considered a given prediction as correct upon visual inspection. **"???"** at DCC = 12Å illustrates the inflection point between 10-12Å, where it is not clear anymore whether predicted pockets within this DCC interval and $I_{rel} \approx 0$ agree with the observed pockets. To facilitate the visualisation of the observed pocket, this one is coloured after the predicted one. Otherwise, for cases where $I_{rel} = 1$ only the predicted pocket would be shown. Despite 1Å intervals were inspected, only representatives of 2Å intervals are shown here for simplicity.

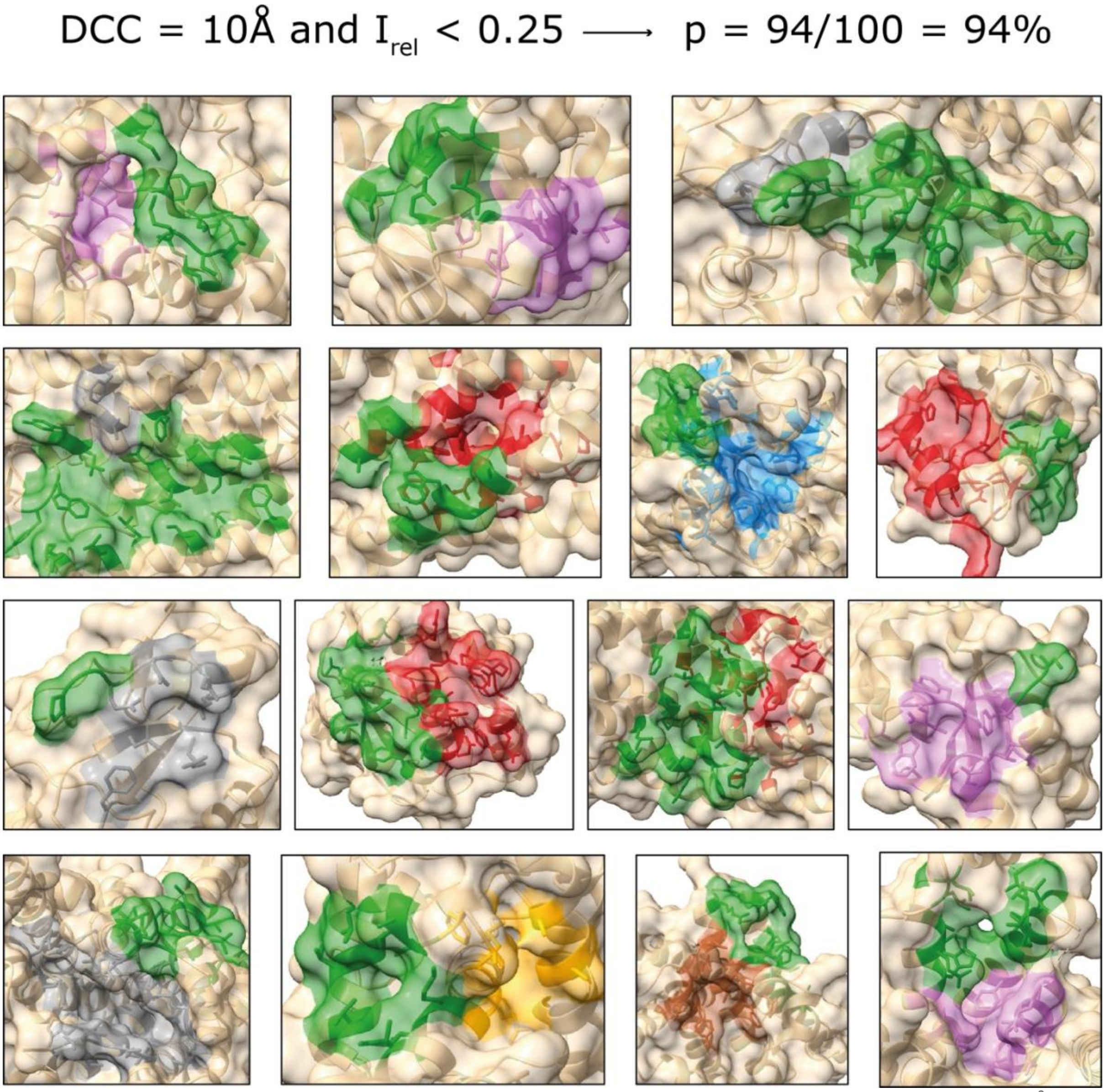

Supplementary Figure 17. Examples of predicted-observed pocket pairs at DCC = 10Å and I_{rel} < 0.25. Out of the 100 examples visually inspected, 94 were considered as correct predictions on the bases that the predicted and observed pockets are adjacent, i.e., their surface area is in contact, and it is therefore easy to imagine a ligand that would bind to this region. LIGYSIS observed sites are coloured in green, and predicted pockets in other colours.

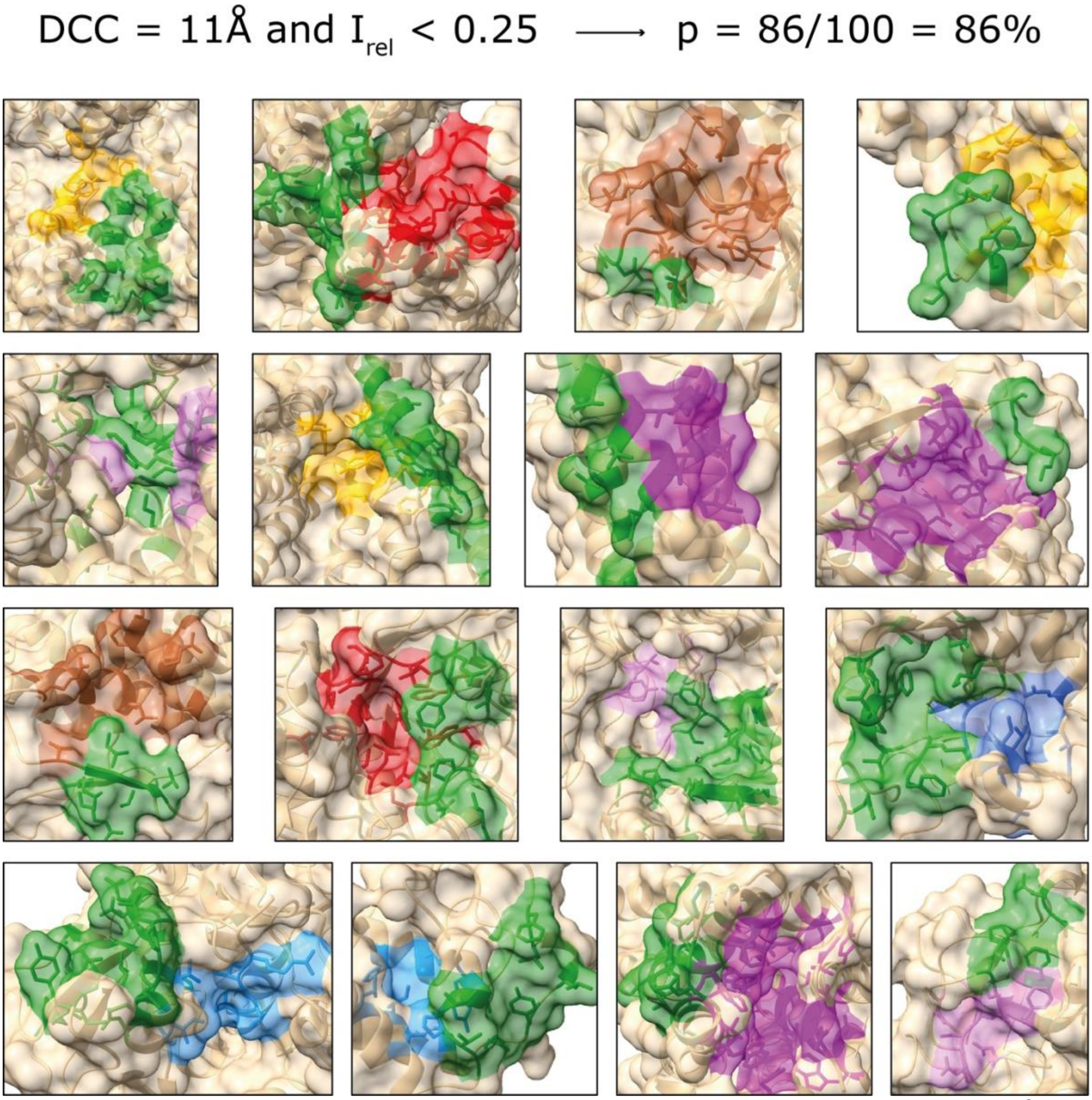

Supplementary Figure 18. Examples of predicted-observed pocket pairs at DCC = 11Å and I_{rel} < 0.25. Out of the 100 examples visually inspected, 86 were considered as correct predictions. LIGYSIS observed sites are coloured in green, and predicted pockets in other colours.

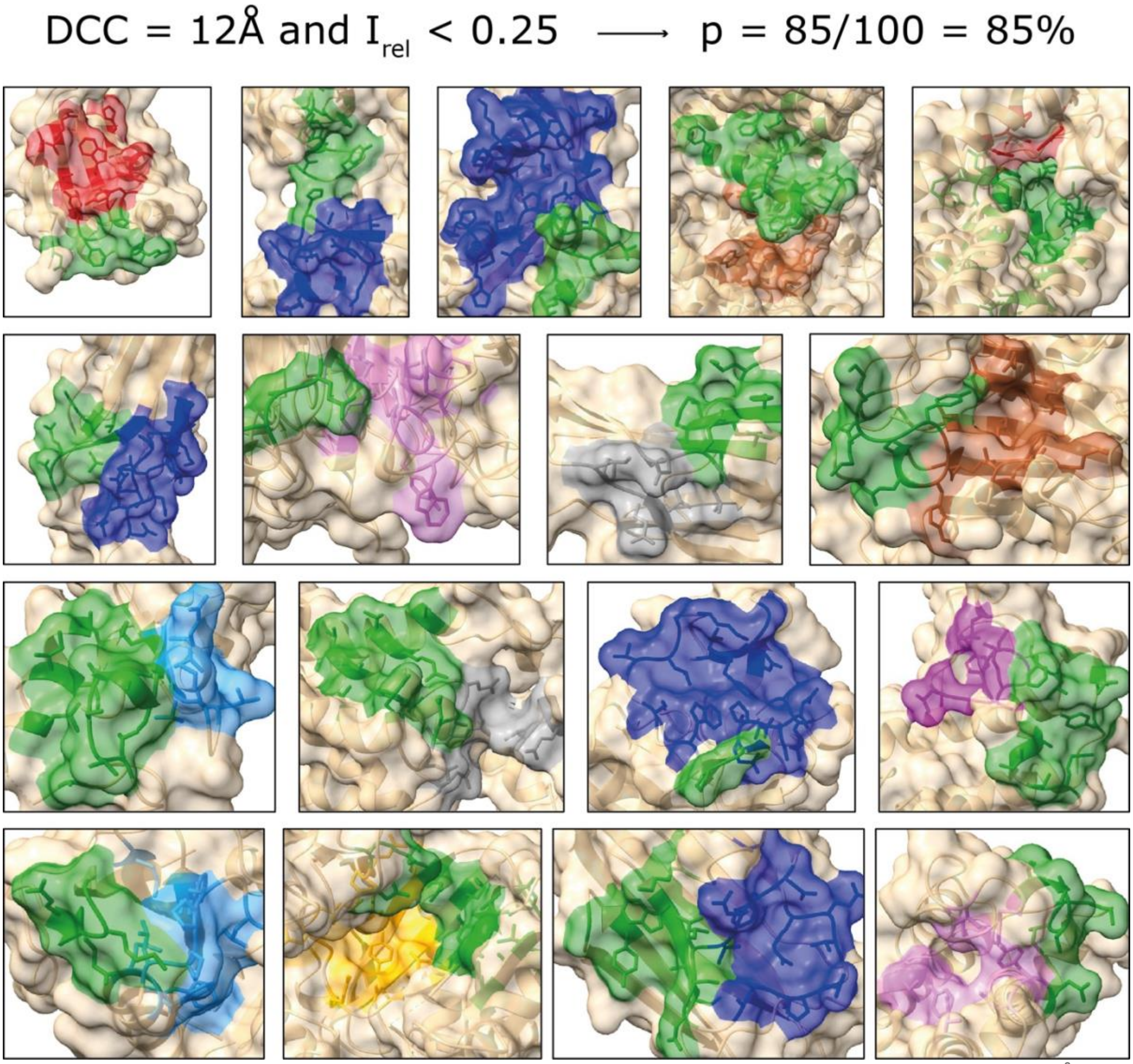

Supplementary Figure 19. Examples of predicted-observed pocket pairs at DCC = 12Å and I_{rel} < 0.25. Out of the 100 examples visually inspected, 85 were considered as correct predictions. LIGYSIS observed sites are coloured in green, and predicted pockets in other colours.

References

1. Carbery, A., et al., *Learnt representations of proteins can be used for accurate prediction of small molecule binding sites on experimentally determined and predicted protein structures.* J Cheminform, 2024. **16**(1): p. 32.
2. Aggarwal, R., et al., *DeepPocket: Ligand Binding Site Detection and Segmentation using 3D Convolutional Neural Networks.* J Chem Inf Model, 2022. **62**(21): p. 5069-5079.
3. Jendele, L., et al., *PrankWeb: a web server for ligand binding site prediction and visualization.* Nucleic Acids Res, 2019. **47**(W1): p. W345-W349.
4. Jakubec, D., et al., *PrankWeb 3: accelerated ligand-binding site predictions for experimental and modelled protein structures.* Nucleic Acids Res, 2022. **50**(W1): p. W593-W597.
5. Krivak, R. and D. Hoksza, *P2Rank: machine learning based tool for rapid and accurate prediction of ligand binding sites from protein structure.* J Cheminform, 2018. **10**(1): p. 39.
6. Le Guilloux, V., P. Schmidtke, and P. Tuffery, *Fpocket: an open source platform for ligand pocket detection.* BMC Bioinformatics, 2009. **10**: p. 168.
7. Schmidtke, P., et al., *fpocket: online tools for protein ensemble pocket detection and tracking.* Nucleic Acids Res, 2010. **38**(Web Server issue): p. W582-9.
8. Sestak, F., et al., *VN-EGNN: E(3)-Equivariant Graph Neural Networks with Virtual Nodes Enhance Protein Binding Site Identification.* arXiv [cs.LG], 2024.
9. Smith, Z., et al., *Graph Attention Site Prediction (GrASP): Identifying Druggable Binding Sites Using Graph Neural Networks with Attention.* J Chem Inf Model, 2024. **64**(7): p. 2637-2644.
10. Kandel, J., H. Tayara, and K.T. Chong, *PUResNet: prediction of protein-ligand binding sites using deep residual neural network.* J Cheminform, 2021. **13**(1): p. 65.
11. Jeevan, K., et al., *PUResNetV2.0: a deep learning model leveraging sparse representation for improved ligand binding site prediction.* Journal of Cheminformatics, 2024. **16**(1): p. 66.

12. An, J., M. Totrov, and R. Abagyan, *Pocketome via comprehensive identification and classification of ligand binding envelopes.* Mol Cell Proteomics, 2005. **4**(6): p. 752-61.

13. Hendlich, M., F. Rippmann, and G. Barnickel, *LIGSITE: automatic and efficient detection of potential small molecule-binding sites in proteins.* J Mol Graph Model, 1997. **15**(6): p. 359-63, 389.

14. Laskowski, R.A., *SURFNET: a program for visualizing molecular surfaces, cavities, and intermolecular interactions.* J Mol Graph, 1995. **13**(5): p. 323-30, 307-8.

15. Capra, J.A., et al., *Predicting protein ligand binding sites by combining evolutionary sequence conservation and 3D structure.* PLoS Comput Biol, 2009. **5**(12): p. e1000585.

16. Yang, J., A. Roy, and Y. Zhang, *BioLiP: a semi-manually curated database for biologically relevant ligand-protein interactions.* Nucleic Acids Res, 2013. **41**(Database issue): p. D1096-103.
